# Functional thermodynamics govern the ligand binding to human cytosolic transport proteins

**DOI:** 10.1002/pro.70637

**Published:** 2026-05-25

**Authors:** Sebastian Michler, Christian Schwieger, Florian Arndt Schöffmann, Dariush Hinderberger

**Affiliations:** ^1^ Martin Luther University Halle‐Wittenberg, Institute of Chemistry Physical Chemistry—Complex Self‐Organizing Systems Halle (Saale) Germany

**Keywords:** electron paramagnetic resonance (EPR) spectroscopy, fatty acid binding protein, protein–ligand binding, thermodynamics, transition states

## Abstract

Traditional binding studies on cellular, molecular transporters often simplify the thermodynamics of binding processes. We present a method that combines spin probing, electron paramagnetic resonance (EPR) spectroscopy and spectral simulations with binding studies and thermodynamics for an in‐depth molecular view into ligand binding and protein functionality. We prove this approach by studying the temperature‐dependent thermodynamics of fatty acids binding to a family of human cytoplasmic transport and signaling proteins, *fatty acid binding proteins* FABP3, FABP4 and FABP5. The proteins were loaded with the radical‐bearing ligands 5‐ and 16‐DOXYL stearic acid (5/16‐DSA) and continuous‐wave (CW) EPR spectroscopy was applied in a broad temperature range. In this methodology, originally developed by us to study ligand binding to synthetic functional polymers, spectral simulations provide the concentrations and equilibria of free, intermediately, and strongly bound ligands. The derivation of temperature‐dependent binding affinities and thermodynamic parameters enables the simultaneous analysis of multiple binding processes. We found and elucidate a thermodynamic preference for loose, entropy‐driven attachment at physiological conditions. The approach delivered complex, temperature‐dependent binding thermodynamics, revealing similarities and discrepancies between various FABP isoforms and concentration regimes. This corroborates the hypothesis that these transport proteins evolved functional thermodynamics with fine‐tuned thermodynamic binding profiles to fulfill respective physiological functionalities. The study delivers important information about protein–ligand interactions in general and establishes EPR‐based thermodynamic analyses as a platform to study and tune native or synthetic polymeric transport systems for advanced applications.

## 1 INTRODUCTION

Fatty acid binding proteins (FABPs) are water‐soluble β‐barrel proteins operating as molecular transporters in the cytoplasm of vertebrates and acting as modulators in the fatty acid signaling pathway (Amiri et al., 2018; Furuhashi & Hotamisligil, 2008). FABP3, FABP4 and FABP5 represent three human isoforms which are mainly found in heart cells, adipocytes and skin cells, respectively (D'Anneo et al., 2020; Makowski & Hotamisligil, 2005). Considering the highly conserved crystal structures of the different isoforms and their similar general function, one might conceive that they expose identical thermodynamic binding pathways. We will partially disprove this expectation in our work. FABP binding dynamics with fatty acid ligands were analyzed earlier by several groups and ourselves in experimental and simulation‐based binding studies (Balendiran et al., 2000; Hanhoff et al., 2002; Michler et al., 2024; Richieri et al., 1993, 1994, 1996a, 1998, 2000). We recently conducted the first comparative continuous‐wave (CW) electron paramagnetic resonance (EPR) spectroscopic experiments on spin‐probed human FABPs (Michler et al., 2024), going far beyond earlier EPR approaches with porcine FABP3 (Fournier et al., 1978; Fournier et al., 1983; Fournier & Rahim, 1985). The combined study demonstrated different binding affinities of FABP3, FABP4 and FABP5 toward the spin‐labeled fatty acids (FAs) 5‐ and 16‐DOXYL stearic acid (5/16‐DSA), with FABP3 showing the highest affinity to the model ligands. We verified the spectroscopic binding studies with results from microscale thermophoresis and docking simulations (Michler et al., 2024). It supported the existing hypothesis that FABPs carry a genetically encoded binding selectivity within their sequences (Agellon, 2024; Michler et al., 2024; Smathers & Petersen, 2011). In EPR simulations we identified two different binding states of FA with FABPs. In the first, intermediately bound state, the ligands are loosely attached to the protein, probably at its surface, whereas in the second binding state they are rather strongly immobilized within the β‐barrel (Michler et al., 2024). The binding appears to be strongly temperature‐dependent. The intermediately bound state is dominant at 35–45°C and was interpreted as the thermodynamically most active state, acting like a dynamic transition state enabling an easy uptake and release of the ligand. Therefore, the binding of FAs seems to be thermodynamically optimized for physiological temperature, referring to 37°C as the standard core temperature of healthy humans (Hymczak et al., 2021; Michler et al., 2024). We specify and differentiate this qualitative hypothesis within our current work and directly take up the previous findings to expand and quantify our analysis of FABP binding. We here illuminate the thermodynamics of binding states and their equilibria, which helps in drawing a complete picture of a functional macromolecular transporter in vitro. We analyze the differences in the thermodynamic binding profiles and set out to develop our analysis into a concept of “functional thermodynamics.”

**FIGURE 1:**
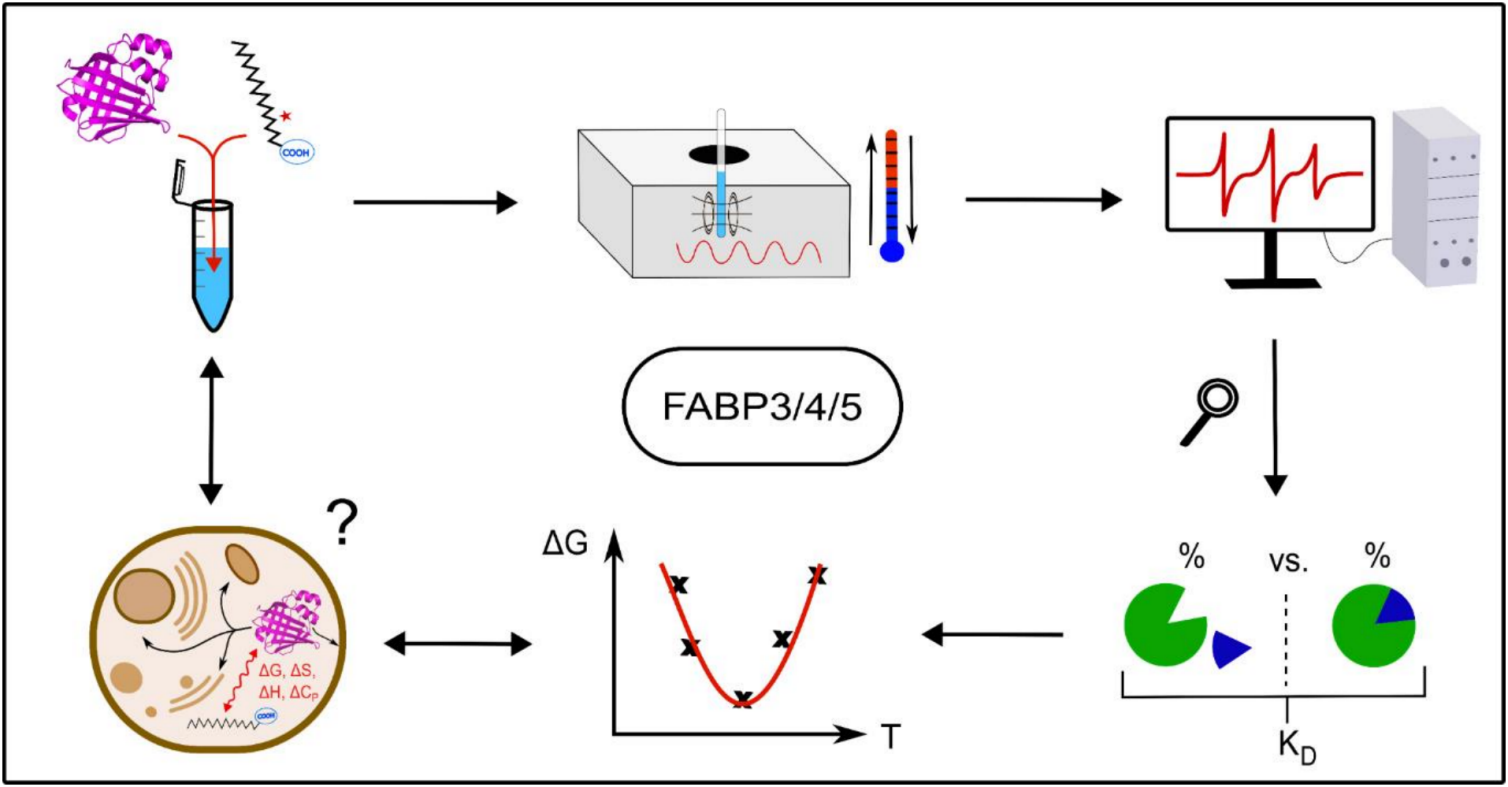
Graphical summary of the experimental procedure followed in this study. FABPs were mixed with the spin probes 5‐ or 16‐DSA in defined concentration ratios and CW EPR spectra of the spin probes were measured at different temperatures. The spectra were simulated, the spectral proportions were extracted and the concentrations of free and bound ligands calculated. *K*_D_ values were calculated for all temperatures and used to derive thermodynamic parameters (Δ*G*°_a_, Δ*H*°_a_, Δ*C*°_Pa_, Δ*S*°_a_). The temperature dependence of these values was interpreted in light of the functionality of FABPs in the cytoplasm of cells.

Prior studies on thermodynamics and temperature stabilities of FABPs focused mainly on conformational protein changes and the comparison of isoforms or mutants from a perspective of protein dynamics. Traditional binding thermodynamics of FABPs with different FAs were analyzed via isothermal titration calorimetry (ITC) and the Lipidex assay (Balendiran et al., 2000; Hanhoff et al., 2002). Additionally, the ADIFAB assay was developed by the group of Alan M. Kleinfeld and later applied in the first studies on binding affinities, thermodynamics and kinetics of FABP mutants with long‐chain FAs (Hanhoff et al., 2002; Richieri et al., 1992, 1993, 1994, 1996a, 1998, 2000). Conformational simulations lead to the hypothesis of FA pre‐adsorption to the portal region of FABPs prior to penetration (Friedman et al., 2006). To the best of our knowledge, highly resolved thermodynamic studies on different binding states and transitions between them, monitored in a large temperature range, were not an object of FABP research so far. Our experimental strategy of spectroscopic‐thermodynamic profiling is illustrated in Figure 1. The extraction of thermodynamics from spectroscopy is well‐established and can be found in the literature especially with fluorescence measurements and to a lower extent also via EPR spectroscopy (Reichenwallner et al., 2017, 2025). Combined spin probing and CW EPR spectroscopy for thermodynamic studies were successfully developed to analyze amphiphilic core‐shell polymers (Reichenwallner et al., 2017) and later human serum albumin (HSA) (Reichenwallner et al., 2025). These studies were the main sources of inspiration for our new study on FABPs. We here analyze a protein class with three isoforms as examples and we compare two ligands in different protein concentration regimes. In analogy to our previous work, FABPs were spin‐probed with the radical‐bearing FAs 5‐ and 16‐DSA. Temperature‐dependent CW EPR‐spectroscopic measurements of the FABP‐spin probe mixtures were complemented by multiple thermodynamic analyses to obtain profiles for all processes—binding and transformation between binding states. The experimental CW EPR spectra consist of overlaying components that quantitatively mirror the distinguishable spin probe species with distinguishable ligand dynamics and environments. These spectral components can be simulated and interpreted as reflecting the simultaneously existing binding states. The EPR spectral simulations represent a crucial step in the procedure. By quantifying the binding states and extracting their equilibrium concentrations from the simulations we were able to calculate binding affinities *K*_a_ and to derive Gibbs energies Δ*G*_a_, enthalpies Δ*H*_a_, entropies Δ*S*_a_ and heat capacities Δ*C*_Pa_ for each conceivable transition between all states. The affinity of the association process is directly observable in the sign and value of Δ*G*_a_ which states whether the association has an exergonic or endergonic character and reflects the stability of the formed FABP‐ligand complex (Du et al., 2016). The binding enthalpy Δ*H*_a_ is sensitive to energetic contributions from non‐covalent interactions such as the breaking or formation of hydrogen bonds, van‐der‐Waals (mainly dispersion) interactions etc. It also includes contributions of the restructuring solvent during ligand‐protein interactions (Cooper & Johnson, 1994). The binding entropy Δ*S*_a_ reflects the loss or gain of motional and conformational freedoms of ligand, protein and solvent molecules during the interaction process and the thermodynamic direction of the process on the macroscopic level. Enthalpy and entropy often compensate each other if one of them experiences a large change during an interaction event, resulting in only moderate changes of Δ*G*_a_ (enthalpy‐entropy compensation). (Du et al., 2016; Kurzbach et al., 2014). Finally, Δ*C*_P,a_ reveals further details about polar or non‐polar (de‐)hydration going along with intermolecular interactions (Prabhu & Sharp, 2005).

The simultaneous observation and thermodynamic analysis of binding processes in coupled equilibria highlights another difference to previous studies and is a key advantage of EPR spectroscopy. Therefore, we re‐define and generalize our experimental strategy as Spectroscopic‐Thermodynamic Analysis of Multiple (binding) Processes (for the sake of brevity: STAMP). EPR‐STAMP applied to FABPs combines EPR spectroscopy and spectral simulations that together allow a characterization of molecular motions and molecular environments. From these EPR data, thermodynamic relations and parameters are derived. This allows a direct, in‐depth view into the molecular binding processes of FABPs from a thermodynamic perspective *of the ligands* with high sensitivity. The approach can easily be transferred to other biological or synthetic polymers or supramolecular assemblies. Due to the exclusive focus on EPR data and the rare application of STAMP in the past, critical discussion of our results and comparison with available knowledge from other studies is mandatory for such a study and is performed in detail. In the course of that, we reevaluate previous findings and discuss our own thermodynamic results for human FABPs in light of functional and genetic considerations. The study unifies the thermodynamic and molecular description of protein–ligand binding and transportation and could deliver design principles, derived from functional thermodynamics. These may facilitate approaches to utilize, mimic and engineer macromolecular transporters in the laboratory.

## 2 RESULTS

### 2.1 Temperature‐dependent binding equilibria of FABPs and fatty acids

For analyzing the temperature‐dependent binding of FAs by FABPs we used the measurement procedure described in our previous work with physiological‐close concentrations and pH values (Michler et al., 2024). Different concentrations of FABP3‐5 (50, 100 and 200 μM) were incubated with constant concentrations (20 μM) of 5‐ or 16‐DSA in HEPES/NaCl buffer at pH 7.5 which is slightly above the average cytosolic pH value (7.2) (Casey et al., 2010). The concentration ratios represent 2.5‐fold, fivefold and tenfold excess of protein to ligand. For clarity we here call them L‐regime (low, 50 μM), M‐regime (medium, 100 μM) and H‐regime (high, 200 μM). Compared to native concentrations which may also differ locally (Zhang et al., 2020), our FABP concentration regimes are slightly below or equal to values found for the human cytoplasm (150–300 μM for FABPs [Richieri et al., 1994; Schwenk et al., 2010; Vork et al., 1993] and below 50 μM for FAs [Schwenk et al., 2010]). The protein‐FA interaction processes in solution are instantaneous and self‐driven. Interactions may include specific and non‐specific non‐covalent interactions of the spin probes with the amino acids of the FABPs. After thermal equilibration, CW EPR spectra were measured for each sample in a temperature range of 0–90°C. Earlier binding studies during temperature increases showed elevated release levels of the ligand in case of FABP3 and FABP4 while the ligand remained partially trapped within the binding pocket in FABP5 and highly concentrated FABP3 (Michler et al., 2024).

Our observed temperature range exceeds the range of former thermodynamic studies and gives the opportunity to also indirectly take denaturation effects or other phase transitions into account. The thermodynamic profiles with values beyond denaturation temperatures of the FABPs are supposed to not just deliver a view into the thermodynamics at physiological temperature but to rather show the physicochemical working mechanics of this protein class in a broader range and to better understand the thermodynamic engineering of these transporters by nature. FABPs show a relatively high thermal stability (Han et al., 2021). FABP3 has been reported to denature at 60–70°C in the apo‐ and 65–70°C in the holo‐form. For FABP4 an aggregation instead of an unfolding mechanism was suggested (Gericke et al., 1997), while for FABP5 no data existed so far. Dynamic light scattering and native gel PAGE in our first study showed that all three FABPs self‐aggregate at 37°C to varying amounts which was promoted by a temperature increase (Michler et al., 2024). We determined the denaturation temperatures of the 16‐DSA‐loaded FABPs via ATR‐IR measurements and principle component analyses applied on the amide I band, delivering values of 62.3°C for FABP3, 70.5°C for FABP4 and 66.1°C for FABP5 (see Figure S35). These temperatures are marked in all figures below.

From the temperature‐dependent series of CW EPR spectra, three spectral components were distinguished by simulations which we described as free (F), intermediately (I), and strongly (S) bound 5/16‐DSA. The components originate primarily from distinguishable rotational correlation times τ_c_ and ^14^N‐hyperfine coupling constants *a*_iso_. τ_c_ describes the rotational motion of a radical in EPR spectroscopy. At room temperature, it is proportional to the strength of interactions with a high molecular‐weight binding partner (Michler et al., 2024; Reichenwallner et al., 2025), leading to a reduction of the dynamics up to complete immobilization. Increasing τ_c_ correlates with decreasing rotational dynamics, which here originates from stronger interactions (Michler et al., 2024). *a*_iso_ acts as a sensor for the environmental polarity. We further defined the sum of (I) and (S) as the total fraction of interacting ligands (T). The relative weights of each component were allowed to vary for different temperatures to generate optimum accordance between simulated and measured spectra. Additionally, τ_c_ and *a*_iso_ were slightly adapted for increasing temperatures to simulate the higher dynamics of the ligands with higher kinetic energy and the destabilization of hydrogen bonds, leading to less polar environments at higher temperatures.

**FIGURE 2:**
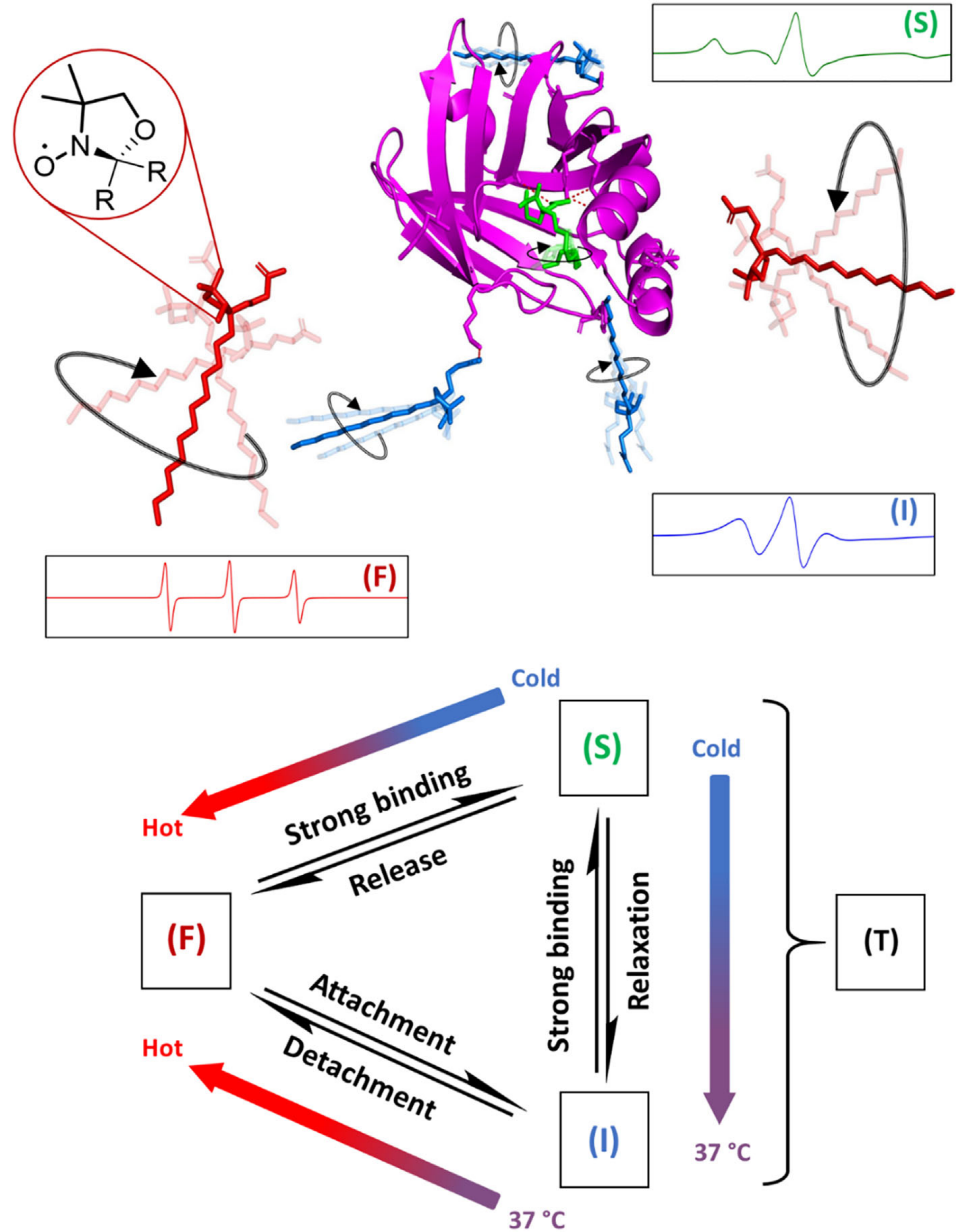
Illustration of free (F), protein‐attached (I) and strongly protein‐bound (S) 5‐DSA molecules with the crystal structure of FABP3. The curved arrows symbolize rotational motion of the ligands; stronger linewidth illustrates higher dynamics. The graphics shows three possible types of ligand attachment which could occur on the surface of the protein (blue): Ligand 1 interacts via headgroup and ligand 2 via alkyl chain with the *portal region*, illustrating hypothetic pre‐binding or ‐release, ligand 3 interacts loosely with unspecific amino acids of the FABP. The strongly bound component within the binding pocket was arranged in a conformation known for stearic acid bound to FABP3 (Matsuoka et al., 2015) (PDB: 4WBK). Below, the temperature‐dependence of equilibrium transitions between the spectral components is shown (F…Free, I…Intermediate, S…Strong, T…Total interacting) for FABP3 with 5‐DSA, but also valid for FABP3 with 16‐DSA.

As an example, the spectral components and their assumed possible interactions with the FABPs in solution are shown in the upper part of Figure 2 with FABP3 and 5‐DSA. The circular arrows illustrate rotational movement of the FA with the radical DOXYL group (red circle). While the 5‐DSA molecules in state (F) and (I) were depicted in linear conformations, the strongly bound ligand (S) is shown in the typical U‐conformation similar to stearic acid (SA), assuming that (S) mirrors the standard binding state that is related to salt bridges, hydrogen bonds and interactions with hydrophobic amino acid residues and known from crystallographic analyses (Matsuoka et al., 2015; Michler et al., 2024; Zanotti et al., 1992). Docking simulations corroborated that 5‐ and 16‐DSA can take conformations similar to those of SA within the FABP binding pockets and can indeed be used as valid model ligands (Michler et al., 2024). It should be noted that it is unknown which conformation or interactions (I) shows in reality. But due to the higher dynamics and—in most systems—higher environmental polarity we interpreted it as a loosely attached pre‐binding or pre‐release state (Michler et al., 2024). In Figure 2, we illustrated three conceivable interactions from a high number of possible configurations, namely dipolar interactions with the portal region or van‐der‐Waals‐type interactions with the alkyl chain, respectively, and an unspecific interaction with the surface at the backside of the FABP. Our attempts to simulate possible weak attachment sites on the protein surface failed due to the high sampling numbers. Recently, several possible binding pathways were found via MD simulation, some of them including transition states of the ligand when entering the binding pocket (Chen et al., 2023). During the binding the ligand was found to be stretched, in opposite to the bound state, which is in good accordance with Figure 2. The MD simulations of Friedman et al. could even show the pre‐adsorption at the surface and the guiding of the ligand toward the binding pocket for a FABP, further substantiating the reliability of our model (Friedman et al., 2006).

As derived from the observed binding states, we here propose the existence of four possible temperature‐dependent equilibrium transitions, each with a direction of association (a) and dissociation (d). For simplification, in this work, we analyze only the association processes and solely in the direction of increasing temperatures. The binding affinities can be calculated in general via the law of mass action (Equation 1) from the equilibrium concentrations [*X*]_e_ of the complex and the free binding partners.

(1) $$K_{a}=\frac{{\left[\mathrm{PL}\right]}_{e,\;X}}{{\left[P_{F}\right]}_{e}\cdot {\left[L_{F}\right]}_{e}}.$$

Within our simple binding model, we can now distinguish between the transitions free to intermediately bound (F‐I), free to strongly bound (F‐S), free to totally bound (F‐T), and intermediately to strongly bound (I‐S). The equilibrium scheme in Figure 2 suggests possible descriptions for the transitions. The I‐S transition is a process of dynamic binding interconversion, comparable to the one described for the FA binding to HSA (Reichenwallner et al., 2025). In general, it is uncertain whether all transitions are physically present or whether some are just imaginary equilibria that are mathematically correct but do not occur in reality.

### 2.2 From CW EPR simulations to thermodynamic parameters

**FIGURE 3:**
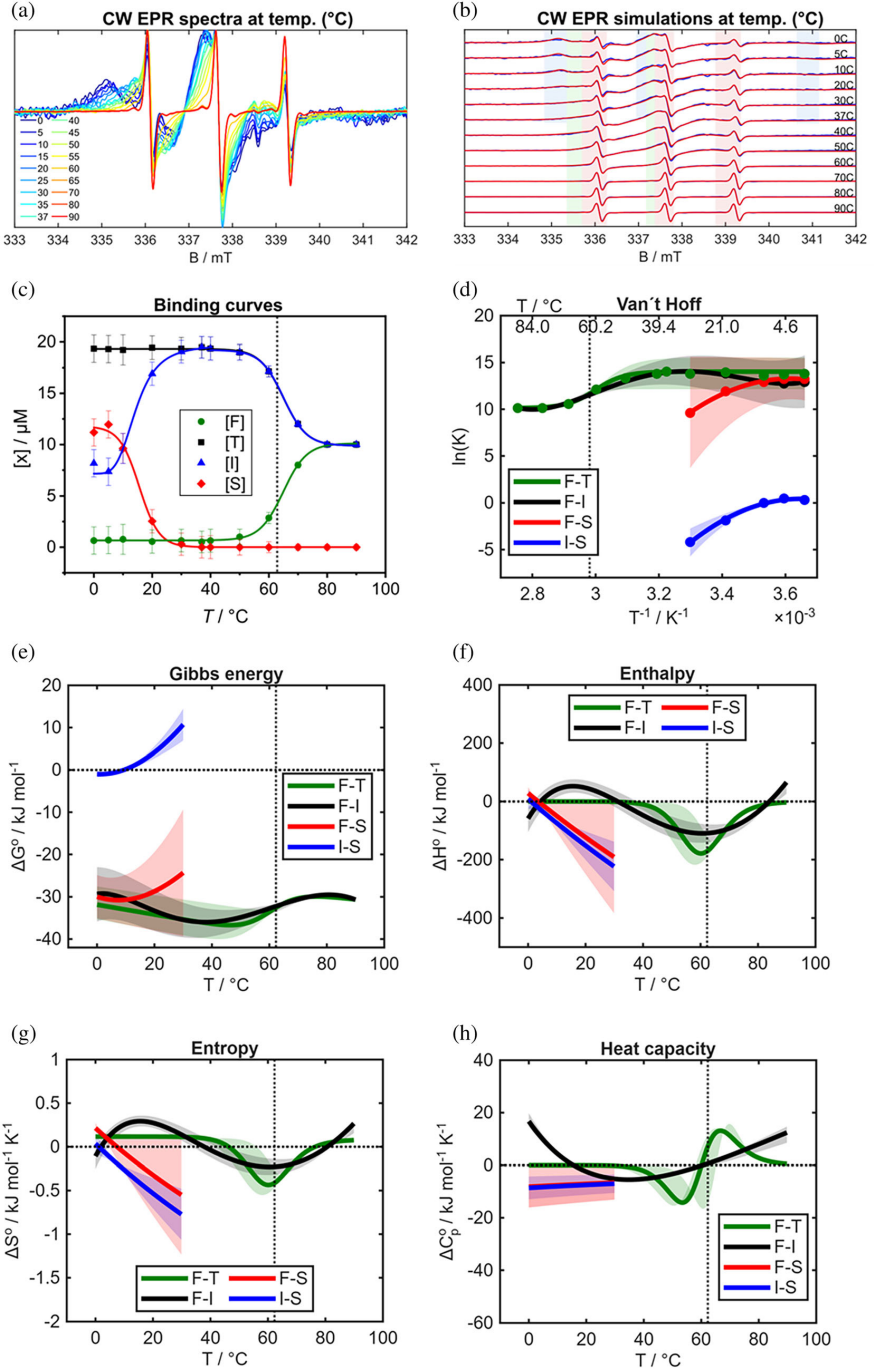
The analytical procedure from CW EPR spectra to thermodynamic profiles for the example FABP3 with 16‐DSA at 50/20 μM. The extraction of thermodynamic parameters from CW EPR spectra starts with the simulation of temperature series (a, b) followed by the plot of the simulation‐derived concentrations of all states against the temperatures in binding curves (c). Both were adapted from our previous work (Michler et al., 2024). From C, logarithmic equilibrium constants K were calculated and plotted in van't Hoff plots (d). Gibbs energies and other parameters (e–h) were directly derived from (d). Above 80°C the curves should be interpreted with more caution as discussed in the main section “Method assumptions, advantages and limitations in discussion.” The original spectra and simulations in a and b and the binding curves in c were similarly published in Michler et al. (2024). Dashed lines at 62.3°C mark the measured denaturation temperature of FABP3. Note that the errors are given as margins of error in (c) and as error bands derived from these initial margins of error as stated in the Materials and Methods Section.

The entire pathway from CW EPR simulations to thermodynamic parameters consists of multiple consecutive calculations as shown in Figure S1. We discuss the strategy applied on the system FABP3 with 16‐DSA in the concentration ratio 50 μM/20 μM (concentration regime L) as an example. The same strategy was applied identically for all FABPx/Y‐DSA systems. The variable definitions and the derivations of the following equations are given with detailed explanations in the Supporting Information. Firstly, the temperature‐dependent CW EPR spectra were simulated, giving the spectral components and component transitions that we briefly described before and in more detail in our previous publication (Michler et al., 2024). All simulated spectra can be found in Figures S2–S19 and the parameters in Tables S3–S15. The temperature‐dependent spectra and simulations of 5/16‐DSA with 100 μM FABPx including the ones shown in Figure 3a,b as well as the binding curves with FABP3 and FABP5 were adapted from (Michler et al., 2024) and re‐used for the new thermodynamic analysis. The temperature‐dependent EPR spectra and simulations with 50 and 200 μM FABPx as well as the binding curves for FABP4 were not published before.

The main, temperature‐dependent numbers that we use from the simulations are the weighting factors Φ_x_ of the respective components *X*. Following these spectral weights, from low to high temperature an exchange of strongly bound by intermediately bound component and an increase of free component is visible. Since the spectra were normalized on a constant total radical and ligand concentration of $\left[L_{t}\right]$ = 20 μM, the concentration [*X*] of each individual spectral component was calculated directly from the simulated component weight $\Phi_{x}$ via Equation (2).

(2) $$\left[X\right]=\frac{\Phi_{x}}{100}\cdot \left[L_{t}\right].$$

The plotting of each $\left[X\right]$ against temperature yields temperature‐dependent binding curves. Those are depicted in Figure 3c and in Figures S20–S22, directly presenting the temperature dependence of each interaction process. Comparable binding curves with simulated proportions were already discussed in (Michler et al., 2024). The binding curve fit functions serve solely as guides to the eye and have no physical meaning. The system in Figure 3c shows a symmetric concentration‐temperature relation of the four states. Starting at low temperature, (S) is replaced by (I) until physiological temperature, indicating a binding reorganization with a first release or relaxation. (I) increases first with rising temperature and reaches a maximum at 35–45°C before it decreases again. Hence, at higher temperatures the ligands are detached to a higher degree. (T) shows a decreasing sigmoidal curve which appears to be an inversion of (F), both meet again at high temperatures and seem to be correlated with the denaturation temperature.

In the next step, temperature‐dependent *K*_a_ values were calculated for all four transitions at equilibrium. For the total binding F‐T, *K*_a_ can be calculated via Equation (3).

(3) $$K_{F-T}=\frac{1}{\left[P_{t}\right]\left(\frac{\left[L_{t}\right]}{\left[T\right]}-1\right)-\left[F\right]}.$$

As already shown in (Reichenwallner et al., 2017), the interconversion process (I‐S) can be directly expressed via Equation (4) with the simulated weights Φ of both states.

(4) $$K_{I-S}=\frac{\Phi_{S}}{\Phi_{I}}.$$

For each individual transition of intermediate (F‐I) and strong binding (F‐S), we have to take the other binding state into account since bound states may co‐appear in coupled equilibria. Considering [I] for F‐S and [S] for F‐I in the calculation of the remaining free ligand concentration, this leads to complex expressions, with Equation (5) for F‐I as an example; the expression for F‐S is mathematically equivalent (see Supporting Information).

(5) $$K_{F-I}=\frac{1}{\left[P_{t}\right]\left(\frac{\left[L_{t}\right]}{\left[I\right]}-1-\frac{\left[S\right]}{\left[I\right]}\right)-\left[F\right]+\left[S\right]-\frac{\left[L_{t}\right]\left[S\right]}{\left[I\right]}+\frac{{\left[S\right]}^{2}}{\left[I\right]}}.$$

We assume that the total concentration of proteins loaded with both types of ligands (I) and (S) can be calculated by the concentration sum of both complexes via ${\left[P_{T}\right]}_{e}={\left[P_{I}\right]}_{e}+{\left[P_{S}\right]}_{e}$ and that (I) and (S) appear mostly on different protein molecules with ${\left[P_{I}\right]}_{e}\neq \text{or}={\left[P_{S}\right]}_{e}$. For simplicity, we further assume 1:1 binding ratio between protein and ligand for F‐I and F‐S. Other, less general binding scenarios would be conceivable as well but are less likely and can be found in the Supporting Information. For temperatures or systems in which the co‐appearing binding state is not populated, Equation (5) simplifies into an equivalent of Equation (3).

The calculated, logarithmic *K*_a_ values were plotted against inverse temperature in van't Hoff plots. This type of plot originates from the van't Hoff relationship (6) and connects binding affinities with the standard Gibbs energies, enthalpies, and entropies of the binding process.

(6) $$\ln K_{a}=-\frac{\Delta G{{}^{\circ}}_{a}}{\mathrm{RT}}=-\frac{\Delta H{{}^{\circ}}_{a}}{\mathrm{RT}}+\frac{\Delta S{{}^{\circ}}_{a}}{R}.$$

As displayed in Figure 3d and for all other systems in Figures S23–S28, the van't Hoff plots of FABPs with 5‐ or 16‐DSA mostly show a non‐linear behavior. Non‐linear van't Hoff plots were reported in the literature for several systems and also for FABPs (Lima et al., 2020; Reichenwallner et al., 2025; Richieri et al., 1998; Tanase et al., 2019). We started by fitting the raw data directly with linear and non‐linear expressions of the van't Hoff equation containing no or constant heat capacity changes (see Supporting Information equations and Figures S32–S34) as they were also used by Richieri et al. (1996b). While these classic, physical fit functions appeared to be sufficient for some of our datasets, they were rather inadequate for others, leading to unresolved transitions in the temperature dependencies (Figure S36 in the Supporting Information). This might be a result of the complexity of the coupled interactions and the high diversity of different temperature dependencies in the processes. As an alternative, we fitted non‐physical, parametrized model functions to all van't Hoff data consistently, a strategy that has been successfully applied before (Reichenwallner et al., 2017, 2025). For these mathematical descriptions of the van't Hoff curves, suitable fitting functions were evaluated by *trial and error*. Polynomial functions of different orders such as for F‐I, F‐S and I‐S in Figure 3d or sigmoidal functions like the Boltzmann function for F‐T in Figure 3d and in one case, an exponential function was used. These functions (Table S1) solely serve the objective to recreate the behavior of the curves; there is no physical meaning behind choosing the functions. Yet, they are crucial for the entire procedure since they serve as the basis for all calculations of the following thermodynamic parameters. We used the non‐linear van't Hoff fits as references to check the general performance of these model fits. Equation (7), derived from Equation (6), enabled the calculation of Gibbs energies for all transitions, while enthalpies were calculated with the differential of Equation (6) according to Equation (8).

(7) $$\Delta G{{}^{\circ}}_{a}=-R\cdot T\cdot \ln K_{a},$$

(8) $$\Delta H{{}^{\circ}}_{a}=-R\cdot \frac{d\left(\ln K_{a}\right)}{d\left(T^{-1}\right)}.$$

From Equations (7) and (8) we could further calculate the entropies via Equation (9), while the derivative of Equation (8) with respect to the temperature gives the heat capacities in Equation (10).

(9) $$\Delta S{{}^{\circ}}_{a}=\frac{\Delta H{{}^{\circ}}_{a}-\Delta G{{}^{\circ}}_{a}}{T},$$

(10) $$\Delta C{{}^{\circ}}_{p,a}=\frac{\mathrm{d\Delta}H{{}^{\circ}}_{a}}{dT}.$$

Together, Gibbs energies, enthalpies, entropies and heat capacities shape the thermodynamic interaction profile from 0 to 90°C in Figure 3e–h. Parameters for transitions F‐S and I‐S are only accessible in the range 0–30°C, since [S] goes down to 0 μM above 30°C. Total, intermediate and strong binding are exergonic in the observed temperature ranges. F‐T and F‐I have global Δ*G* minima at 46 and 38°C, respectively, which are close to physiological temperature and confirm the thermodynamic prevalence of both processes compared to F‐S. In the literature, typical values of −33 to −38 kJ/mol were found for FAs binding to FABPs, being in good accordance with our values (Richieri et al., 1995). With lower and higher temperatures, F‐I and F‐T become more endergonic. F‐S seems to be a less preferred binding process at 37°C with higher Δ*G* values, although the errors of this transition are relatively large. The I‐S interconversion is located at the endergonic/exergonic border, marking Δ*G°* = 0 kJ/mol, and becomes even more endergonic when reaching 30°C. Hence, during a cooldown from physiological temperature, the inverse S‐I interconversion would become more endergonic.

The binding enthalpies, entropies and heat capacities in Figure 3f–h reveal further information. F‐S and I‐S are mostly exothermic and show a linear decrease of Δ*H*° with rising temperature. F‐S might be entropically favored at low temperature, but becomes soon dominantly unfavored with $\Delta S{{}^{\circ}}_{F-S}$ decreasing similarly as $\Delta H{{}^{\circ}}_{F-S}$. I‐S is entropically unfavored at all temperatures but shows the same temperature evolution. Both transitions have almost constant negative heat capacity changes. These results can be confirmed by classic van't Hoff fits (see Supporting Information, Table S16). The negative entropy differences of I‐S outweigh its negative enthalpy differences except at very low temperatures. On the opposite, the less negative entropy differences of F‐S are over‐compensated by the negative enthalpy differences. This explains the very different Δ*G*° curves of F‐S and I‐S. F‐I shows three crossings of zero enthalpy with the most interesting one from endo‐ to exothermic nature at around 32°C. Endo‐ and exothermic areas at very low and high temperatures should be treated with caution due to the larger fitting errors at the dataset borders. The heat capacity difference of F‐I reaches a negative global minimum at 35°C, indicating maximum dehydration of non‐polar groups (Prabhu & Sharp, 2005; Reichenwallner et al., 2017). This is in good accordance with a conceivable hydrophobic effect from the ligand's perspective and the removal of water molecules from FA and FABP surfaces and especially between their interface during intermediate binding (Friedman et al., 2006). F‐T is non‐enthalpic and entropy‐driven until transitions occur at 35–40°C. Above that temperature the total binding is exothermic and negatively entropic with a global minimum at 60–65°C before it approaches 0 kJ/mol again when the expected denaturation proceeds. This minimum is co‐localized with the local enthalpy and entropy minima of F‐I, with changes of Δ*C*_p_° from negative to positive signs, and with the expected denaturation temperature of FABP3. The overlap of enthalpy and entropy minima is an example for the enthalpy‐entropy compensation phenomenon which was explained in the introduction and reported in many other studies before, for other systems and for FABP binding, too (Du et al., 2016; Richieri et al., 1998). Entropy and enthalpy curves of F‐I seem to be similar, but a closer look reveals zero‐crossing shifts which might be crucial for the binding mechanism. F‐I becomes entropically unfavored slightly above physiological temperature while its enthalpy becomes negative slightly before (31°C). This suggests a change from entropy‐to enthalpy‐driven binding in the range 31–38°C where the Gibbs energy reaches its exergonic minimum. The slight shift of entropy to higher temperatures should explain the exergonic plateau at physiological temperature. Both, hydrophobic effect and electrostatic interactions might govern the intermediate binding. Interestingly, all earlier thermodynamic studies on FABPs found dominating enthalpic contributions in the binding profile at 30–37°C (Balendiran et al., 2000; Richieri et al., 1995, 1998). This temperature is very close to the enthalpy‐entropy transition temperature found in here. However, neither ITC studies nor the ADIFAB assay are able to observe entropy‐driven unspecific interactions which may not necessarily go along with a protein conformational change. Our results illuminate new aspects of the binding mechanism and indicate that only parts of the thermodynamic picture were discovered before. Enthalpy and entropy seem to be well‐balanced to generate a minimum in Gibbs energy and a maximum in affinity for intermediate binding. F‐T instead might be more robust, with a broader, slightly shifted plateau of Δ*G°*. However, this shift compared to F‐I might also stem from the different fitting functions.

In summary, the thermodynamic profile in Figure 3 draws a complex thermodynamic picture of enthalpy‐entropy compensation, well‐balanced thermodynamic parameters, Gibbs energies of binding with minima at 38–46°C and phase transitions at 31–38°C and 60–67°C. The transitions cover large changes in enthalpy differences from −223 kJ/mol for I‐S at 39°C to +62 kJ/mol for F‐I at 90°C while values around −12 kJ/mol and −25 to −60 kJ/mol were found for different FAs and FABPs in the literature (Balendiran et al., 2000; Lalonde et al., 1994; Richieri et al., 1995). Indeed, our F‐I transition has an enthalpy difference of −31 kJ/mol and F‐T lies at −5 kJ/mol at 37°C. Both are located at least in a comparable range of energies. Given the use of completely different methods and additional components (ITC, ADIFAB) in their systems, as well as our different, more complex transitions, a comparison with our energetic values may appear difficult. Yet, it seems that the previously used methods reflect parts of the thermodynamic properties of the overall system that we study here.

Thermodynamic profiles were also determined from 5‐DSA based interaction studies to check the influence of the label position on the binding thermodynamics. While we found label‐independent, general thermodynamic trends for the FABPs, shifts and other changes could also be found between 16‐ and 5‐DSA (see Figures 3 and S24). Transition temperatures and energetics are slightly dependent on the position of the DOXYL group during the interaction processes. This occurs probably since the degree of hydration, the local polarity, and even the affinity of the entire ligand might change on the molecular level for a different label position. Therefore, the choice of the ligand in our STAMP approach is important and should be consistent when comparing different systems. In light of this, we refrain from a more detailed analysis of the thermodynamic differences derived between 5‐ and 16‐DSA, respectively, and refer to the profiles in the Supporting Information for readers who are more interested in this specific topic.

### 2.3 Binding thermodynamics depend on the FABP concentration and the isoform

One fundamental question that appeared after this first analysis of a FABP‐ligand system treated the choice of the FABP concentration regime and the isoform which might affect the thermodynamics. After analyzing the above defined L‐regime with 50 μM FABP3 concentration we analyzed whether otherwise identical samples in the M‐ and H‐regime of protein concentration (Figure S23) show differences in their profiles. At both higher concentrations, the transitions F‐T and F‐I are only accessible in the range from 30 to 90°C. They show identical thermodynamics in the M‐regime and are entropy‐driven at physiological temperature. They further share a Gibbs energy minimum of −38 kJ/mol at 40°C, and a slightly lower enthalpy/entropy minimum at 55°C. The enthalpies approach 0 kJ/mol at 40 and 90°C, the entropies are negative between 40 and 72°C. All transitions remain exothermic in the entire temperature ranges in the M‐ and H‐regime. In the L‐ and M‐regime, F‐I and F‐T exhibit relatively similar Gibbs energy, enthalpy and entropy curve shapes, but in the H‐regime they exhibit a flatter $\Delta G{{}^{\circ}}_{a}$ curve with no clear minimum. All three transitions show noticeably lower Gibbs energies in the H‐regime. F‐T and F‐I become more endergonic at physiological temperatures but more exergonic at higher temperatures when switching to the H‐regime. Another difference between the regimes can be found in the exergonic‐endergonic phase transition of I‐S which is shifted to a higher temperature of 13°C from L‐ to M‐regime, and further to 25°C from M‐ to H‐regime, showing also a larger plateau at 0 kJ/mol. Obviously, the interconversion from the intermediately to the strongly bound state seems to be more robust and more exergonic with rising protein concentration while its enthalpic and entropic properties also change significantly from the L‐ to H‐regime (see also in the Supporting Information).

**FIGURE 4:**
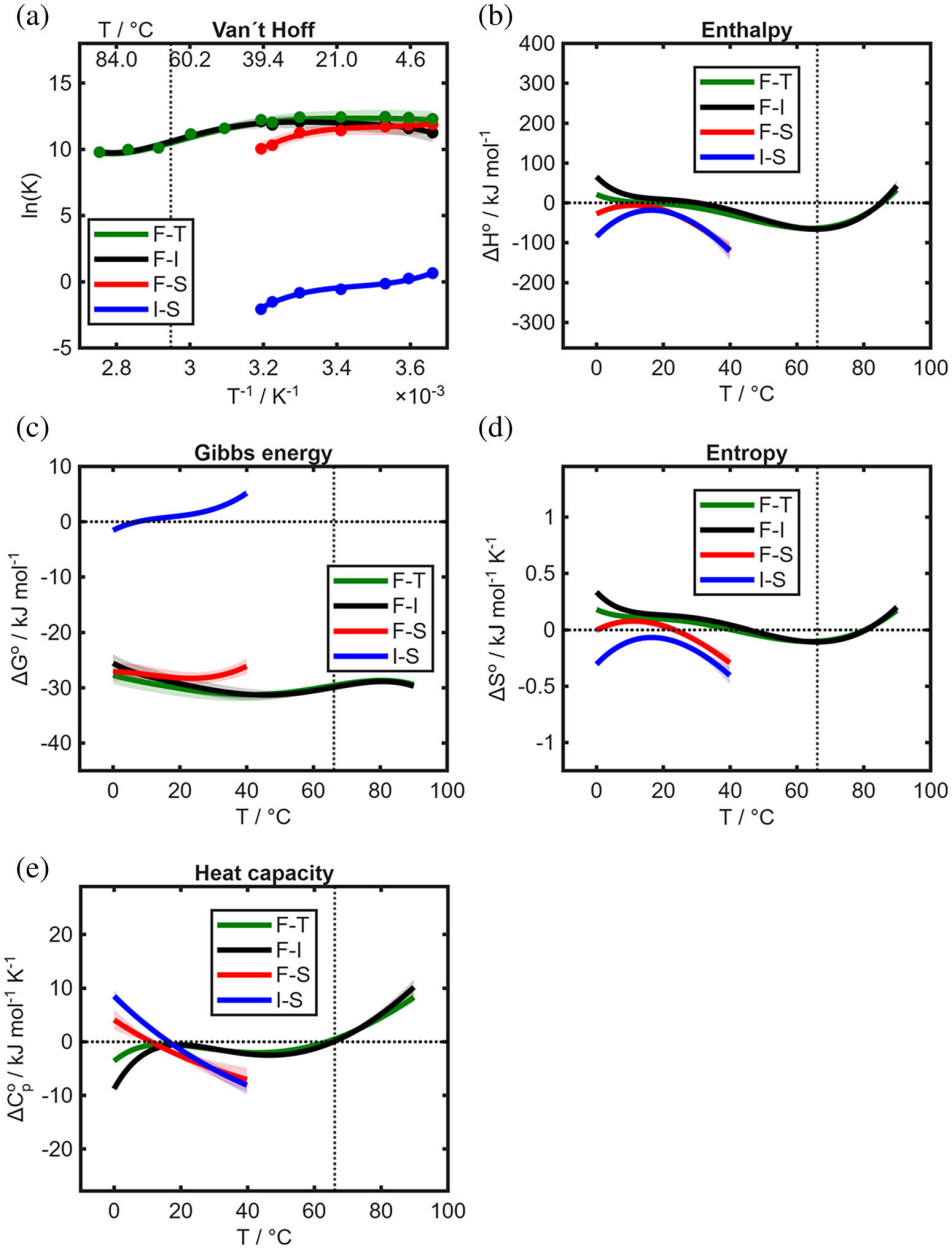
Thermodynamic binding profile of 20 μM 16‐DSA with 50 μM FABP5 (L‐regime of concentration). Transitions F‐T and F‐I were accessibly in the temperature range 0–90°C, while I‐S and F‐S were only accessible from 0 to 40°C since [S] does not occur above 40°C for this system. (a) Van't Hoff plots for all transitions, (b) temperature‐dependent Gibbs energy changes, (c) enthalpy changes, (d) entropy changes, and (e) heat capacity changes. Margins of error of all thermodynamic plots are shown as error bands as stated in the Materials and Methods Section.

As a next step, we change the isoform and analyze the thermodynamic profile of FABP5 with 16‐DSA in the L‐regime (Figure 4). In the direct comparison of Figures 3 and 4, differences and similarities occur between the thermodynamic profiles. The overall profile of FABP5 seems to be fairly similar to that of FABP3, especially regarding van't Hoff‐ and Δ*G*°_x_‐curves, although the error bands are much thinner. But the van't Hoff curves exhibit different curvatures, and the transitions F‐I, F‐S, and F‐T are more endergonic. Especially F‐S and I‐S show stronger differences to FABP3.

Besides the clear difference in the Gibbs energy values, small deviations between the isoforms occur in enthalpy, entropy, and heat capacity, especially below and above physiological temperatures (see Supporting Information for a detailed analysis). This observation suggests fine‐tuned differences in the molecular interactions and the temperature‐dependent binding mechanism between the protein isoforms, while the overall thermodynamic binding profile shows very similar general trends. The interactions leading to I‐bound states are conceived to be unspecific for intermediate binding (see above, mainly entropic/hydrophobic effect) with only slightly negative enthalpic profiles, and a bit higher exothermic binding in case of total binding. Direct strong binding is much more exothermic but decreases entropy instead due to the reduction of the degrees of freedom and the anchoring of the ligand inside the binding pocket through hydrogen bonds or salt bridges. Such interactions often go along with negative enthalpy changes (Du et al., 2016). Hence, this process of direct strong binding is enthalpy‐driven. The same appears to hold true for the interconversion from intermediate to strong binding. The negative heat capacity differences indicate non‐polar dehydration, appearing because all processes are either dominated by removal of the hydrophobic ligand from the water phase or its interaction with the hydrophobic binding pocket of the FABPs. This effect seems to be maximized for the movement into the binding pocket (F‐S and I‐S).

If we switch from the L‐ to the M‐regime for FABP5/16‐DSA (Figure S27), the specific thermodynamic picture changes again, showing concentration dependence as well. The van't Hoff curves reveal simpler polynomial shapes, again leading to curve convexities and enthalpy/entropy curves different from those in the previous systems. The Gibbs energies for F‐I form a plateau at 50–70°C in this case, but F‐T exhibits a long plateau with a minimum at 40°C, similar to FABP3. Compared to the FABP5‐system in the L‐regime, F‐S and I‐S at M‐concentration show strongly altered enthalpy curves, but I‐S has a similar exergonic‐endergonic transition at 7°C. They also show no or even positive Δ*C°*_p_ values. Their entropy differences remain constantly negative (F‐S) or even increase (I‐S) now, in opposite to the FABP‐16‐DSA systems at lower concentrations. I‐S even has a negative–positive entropy transition at 39°C. The higher protein concentration could lead to different protein surface interactions and environments which could reduce the entropy loss during strong binding from the intermediate state.

As a final example, FABP5 with 5‐DSA shows concentration‐dependent thermodynamics, too (Figure S28). It can be estimated that Δ*G*° deviates stronger for all transitions with higher temperatures, but into different directions, proving stronger concentration dependence of FABP5 binding thermodynamics. Enthalpies and entropies remain relatively constant at 40°C, but especially at 60°C, they also show strong divergence. In summary, a change from L‐ to M‐regime and further to H‐regime can induce only small or strong changes in enthalpy, entropy, and Gibbs energy, depending on the system. The Gibbs energies of F‐T and F‐I change especially in the H‐regime. Along with the energetics, also the transition temperatures change. Most systems adhere to this picture of concentration dependence, but in different manifestations, and the results show the importance of isoform and concentration‐dependent analyses and interpretations.

### 2.4 The functional thermodynamics of FABP3, FABP4 and FABP5 in comparison

**FIGURE 5:**
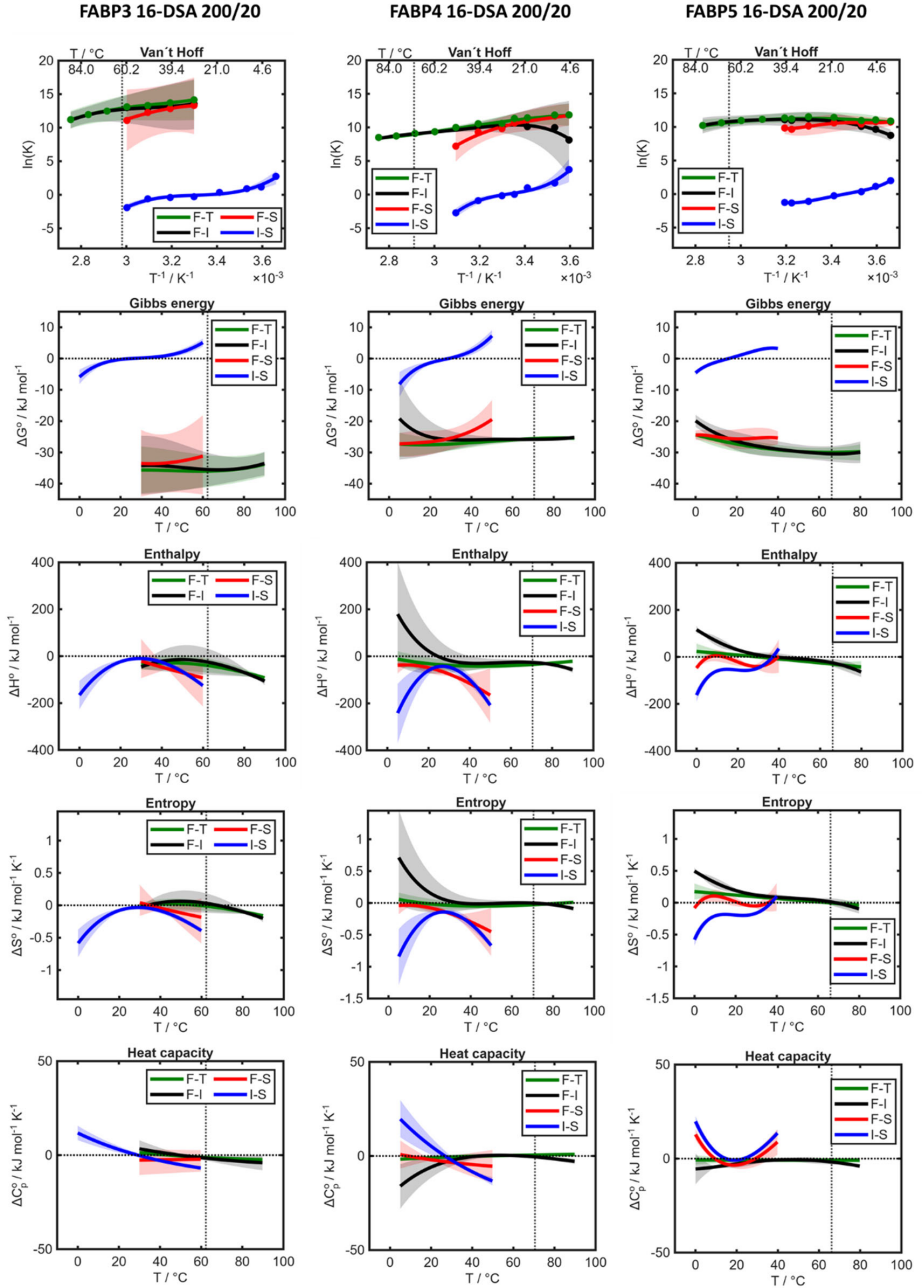
Overview of thermodynamic binding profiles of 20 μM 16‐DSA with 200 μM FABP3, FABP4 and FABP5. Each column represents the profile for 200 μM of a FABPx in combination with 20 μM of 16‐DSA. Phase areas with different signs of thermodynamic parameters are separated by dashed zero‐lines. Margins of error of all thermodynamic plots are shown as error bands as stated in the Materials and Methods Section.

After assessing the thermodynamic changes that ensue from different concentration regimes and the transition from FABP3 to FABP5, we now compare in more detail whether all three protein isoforms have similar or completely different thermodynamic profiles. For a final, comprehensive view on the three FABPs we focus on the H‐regime and keep 16‐DSA as the consistent ligand. The three profiles of interest with 16‐DSA and the H‐regime of FABP concentration are shown in Figure 5 while all other systems can be compared in the Supporting Information. Some previously found trends can now be generalized for the FABPs:

(1) Intermediate, strong and total binding remain all exergonic in the entire available temperature ranges, while the interconversion from intermediate to strong binding always changes from exergonic to endergonic behavior below physiological temperatures. (2) A characteristic trend that can be found in many of the systems observed is that intermediate binding is entropy‐driven below and at physiological temperatures. It is further always enthalpy‐driven at higher temperature while total binding is enthalpy‐driven in some cases (FABP4), but fully entropy‐driven in others. (3) Direct strong binding often has a slightly higher entropy but also a higher enthalpy, although both remain negative at most temperatures. This indicates direct strong binding as an enthalpy‐driven process, except for FABP5 from 2 to 21°C and again above 40°C where it is entropically favored. F‐S becomes more endergonic with rising temperature. (4) I‐S seems to be mostly enthalpy‐driven below or close to physiological temperature. If we take a simple molecular perspective, intermediate binding might be driven mostly by the hydrophobic effect, which is based on increasing entropy due to the release of water molecules and the association of non‐polar molecule domains. This effect appears to be even stronger for intermediate binding than for total binding, because total binding includes also the re‐formation of bonds inside of the binding pocket which are enthalpy‐driven but can be less, zero‐ or even negatively entropic. The heat capacity changes are different for FABP5, remaining positive instead of turning negative like for FABP3 and FABP4. (5) Total binding is slightly more exergonic than intermediate and strong binding are. (6) Intermediate and total binding show differences in enthalpy and entropy depending on the FABP. In case of FABP3, enthalpies are negative for both, for FABP4 they are positive until 25°C for F‐I and for FABP5 they are positive for both until 30–37°C. This indicates a variable, FABP‐dependent nature of the intermediate binding. The weighting of the enthalpies and entropies of F‐I, I‐S, and (possibly also) F‐S in their interplay close to physiological temperature might be the key to the functional thermodynamics and can explain how the binding mechanism of FABPs has been adapted to their working conditions. The strongest differences between the FABPs can be found in the heat capacities which can change their signs multiple times. Further thermodynamic similarities and differences between the FABP isoforms can be concisely summarized and are given in the Supporting Information. For further analysis of the complex characteristics and anomalies we refer to the profiles in Figures S23–S28. We will refrain from interpreting features and transition temperature changes in detail, since some differences might stem from the fitting process, especially in the heat capacity curves.

## 3 DISCUSSION

### 3.1 Interpretation of functional thermodynamics of the FABPs in their native environments

We start the discussion from the obvious question: can the amino acid sequence of FABP isoforms be directly correlated with their thermodynamic binding profiles? The binding thermodynamics under identical conditions are compared pairwise between FABP3/4, FABP3/5 and FABP4/5. To this end, Δ*G*_a_°, Δ*H*_a_° and Δ*S*_a_° for all three pairs were analyzed for two concentrations of each protein (FABPx/16‐DSA 50/20 (L‐regime) and 200/20 (H‐regime)) as examples (see Figures 5 and S29). It is obvious that Gibbs energies exhibit only small changes between the isoforms, with FABP4 and FABP5 showing the smallest differences. At physiological temperature they have almost identical Gibbs energies, enthalpies and entropies. Interestingly, these two isoforms share sequence identities of only 55%, while FABP3 and FABP4 share the highest sequence identity (65%) but show a stronger difference in Δ*G*° of total and intermediate binding. This picture changes slightly when comparing enthalpies and entropies. We find fewer differences for FABP3 and FABP4 in the transitions F‐S and I‐S than the other pairs which exhibit strong deviations in their enthalpy and entropy changes for F‐S and I‐S, especially at 20–30°C. This observation might indicate that the strong binding reflects the energetically most conserved process. FABP3 and FABP5 share the lowest identity (51%) and similarly high Δ*G*_a_° variations for F‐T and F‐I as FABP3/FABP4. Obviously, it is not trivial to correlate sequence identities directly with thermodynamic similarities. A high similarity in Δ*G*° and thereby in the binding affinity does not solely correlate with a higher sequence identity! The enthalpic and entropic temperature evolution has to be considered as well.

One may now draw possible conclusions for the FABP‐ligand binding in native environments as indicated as the final step in the procedure of Figure 1. FABP4 and FABP5 are co‐localized in some tissues and were conceived to operate synergistically. (Furuhashi & Hotamisligil, 2008; Haunerland & Spener, 2004; Yu et al., 2016) We already found unspecific binding similarities between the two isoforms in our first study and could now quantify and substantiate the thermodynamic similarity in total and intermediate binding. The similarity analysis of all three FABP sequences (see Figure S30) reveals pairs or triplets of conserved, identical amino acids clustered over the sequence. Especially, the first α‐helix (α1) exhibits high sequence similarity. For a detailed comparison of the physicochemical properties, only the non‐identical residues of all three FABPs were analyzed by different domains and separated into solvent‐exposed and binding pocket‐related ones. The second helix (α2) and the β‐sheet‐turn (TT) regions β2‐TT, β4‐TT, β6‐TT, β8‐TT, and β9‐TT‐β10 (see Figure S31) show significant sequence deviations. The total FABP5 sequence is on average more hydrophilic with a grand average of hydropathies (GRAVY) of −0.458, compared to FABP3 and FABP4 which show very similar hydropathies (GRAVY of −0.265 and −0.249, Table S2). They also share temperature‐dependent similarities in the F‐S and I‐S transitions; especially, F‐S is almost identical.

Many of the varying amino acid residues are located as clusters on the protein surface and exposed to the surrounding bulk solution. Such differences on the surfaces of FABP isoforms might govern intermediate binding via enthalpically and entropically dominating interactions and might lead to the observed variety in intermediate binding thermodynamics for some conditions. The non‐identical surface‐exposed amino acids of FABP5 are slightly more negatively charged and more hydrophilic. FABP3 has the most hydrophobic surface; the surface GRAVYs of FABP3 and FABP4 are closer to each other than to that of FABP5. All surfaces are projected to be more hydrophilic than the total sequence, which can be expected for water solubility. α2 and β9‐TT‐β10 are significantly more hydrophobic than the total sequences for all three isoforms, arguing for a conserved thermodynamic function of these hydrophobic areas, for example, in the loose attachment of hydrophobic FA chains. The pre‐adsorption to the portal‐connected residues was simulated by Friedmann et al. and explained with higher hydrophobicity and dynamics of this protein domain (Friedman et al., 2006). The α2 residues are mostly oriented to the bulk solution and are on average more hydrophobic for FABP5 (Figure S31). This could result in stronger non‐polar interactions and increased levels of entropy‐driven intermediate interactions with FA chains. However, the Gibbs energy of F‐I is lower for FABP5 since the entropy is compensated by positive enthalpic differences. Friedmann et al. suggested that both electrostatic headgroup and Lennard‐Jones chain interactions govern the adsorption (Friedman et al., 2006). β4‐TT and β6‐TT are most hydrophilic for FABP5 (Table S2), arguing for facilitated polar interactions of FA headgroups with these regions or adapted thermodynamics to more hydrophilic environments. These domains are more similar and more hydrophobic for FABP3/FABP4. Some residues of β6‐TT, which are located on the opposite to the portal region, are correlated to water exchange between bulk solution and the interior binding pocket and were identified as possible adsorption sites by Friedman et al. (2006). Furthermore, β6‐TT, β8‐TT, and β9‐TT‐β10 are most hydrophobic for FABP3. FABP4 has the most hydrophilic β8‐TT domain and the most hydrophobic β2‐TT domain. β8‐TT is much more positively charged for FABP4/FABP5, which might also affect electrostatic surface interactions. The largest deviating domain of FABP3/FABP4 is β9‐TT‐β10, which is similar for FABP4/FABP5 and located close to the portal region. It could be important for intermediate binding and substantiate the high thermodynamic similarities of FABP4/FABP5 regarding total and intermediate binding. Their average Δ*G*° values are closer; their Δ*H*° and Δ*S*° curves show more similar shapes compared to FABP3. Intermediate and total binding to FABP3/FABP4 have low Δ*G*° similarity but higher Δ*H*° and Δ*S*° similarity at low temperatures, which is reversed at higher temperatures. The binding pockets of all FABPs are more hydrophobic than the total sequences. They are highly conserved for FABP3/FABP4 (see Michler et al., 2024), which might be reflected by the thermodynamic similarities in strong binding and interconversion that we observed. FABP4 has the most hydrophobic and FABP5 the most hydrophilic binding pocket environment considering only residues close to the strongly bound ligand. This could directly affect the thermodynamics of strong and total binding. In the case of FABP4 and FABP5, especially the enthalpies and entropies of strong binding and interconversion differ significantly. The binding pocket GRAVY values are further apart for this pair than for other pairs. Finally, FABP3/FABP5 show the strongest sequence deviations, especially in the domains α2, β6, and β9‐TT‐β10, a similar F‐I and F‐T relation like FABP3/FABP4, and stronger deviations in F‐S and I‐S compared to both other pairs.

An important factor of FABP specialization could be the water content of different tissues. Adipose tissue has a water content of 5%–20%, and skin tissue contains 60%–76% water (Altman & Dittmar, 1972; Pethig & Kell, 1987). We already stated that this could affect binding adaptations of tissue‐dominating FABP isoforms, in this case FABP4 and FABP5 which are often co‐appearing (Furuhashi & Hotamisligil, 2008; Yu et al., 2016). The thermodynamic similarities of FABP4 and FABP5 might be rather connected to a functional adaptation than to conserved sequence identities. The hydration of the FAs, the FABP surfaces, and the binding pockets might play an important role in the binding mechanism (Antúnez‐Argüelles & Robles‐Gómez, 2020; Friedman et al., 2006) leading to some of the varying thermodynamic features we observed. Since the transport and release of ligands by FABPs are important for signaling and metabolic processes, up‐ and down‐regulations of FABP expression and compensation effects between different FABP isoforms could play important roles for the origin and development of various diseases (Furuhashi & Hotamisligil, 2008). This may go along with varying cytosolic concentrations, disturbed metabolic mediation, and signaling in cells and could be directly correlated with disturbed functional thermodynamics. As we saw earlier, lower or higher FABP concentration can lead to changes in the thermodynamic binding pathway. Local concentration differences might also appear between cytoplasm and membranes and could also mediate different thermodynamics for the promoted binding or release of ligands at the different cell destinations of the FABPs, suggesting a transport mechanism controlled by protein concentration. The development of individual functional thermodynamics for each FABP isoform might be crucial for the functionality and the specialization of the different FABPs. In summary, our here established thermodynamic picture of cellular molecular transporters highlights the intermediate binding as a dominating, mostly entropy‐driven transition process which mediates strong binding. It appears to be an efficient mechanism, highly plausible to also appear within real cells. Further connections to tissue‐specific specializations and disease relations are still of speculative nature but we can safely state correlations of the biochemical transport mechanism of FABPs with thermodynamics that were previously not detectable.

### 3.2 Method assumptions, advantages and limitations in discussion

It is necessary to critically discuss and evaluate the results of this study with respect to the method in general. Since the experimental and analytical procedure consists of multiple subsequent steps, error propagation has a strong influence on the final results and their interpretation as well. The simulations appear to be a critical step and should be checked rigorously. This was done by multiple refinements and comparisons with test simulations. In our calculations of component concentrations, we assume that intermediate and strong binding follow a simple binding model of a 1:1 ligand/protein ratio and without mutual cooperation effects. But we included the concept of coupled equilibria and the co‐existence of both states. One question that we did not raise explicitly is the reversibility and direction of the interaction equilibria. EPR measurements confirmed well the reversibility of association and dissociation at moderate temperatures, partially reversible processes even after reaching very high temperatures, but also irreversible changes. These are probably connected to denaturation, protein aggregation, or possibly gelation effects. Due to increasing irreversibility, the underlying binding models might have reduced accuracy at these extreme temperature regions. By assuming a consistent radical and protein concentration in our samples we can extract the proportions of free and bound radical in our solutions with high sensitivity, selectivity, and resolution. Further assuming accurate thermodynamic calculations and a correct general concept of thermodynamics that we underlay, the derived parameters should be well reliable and the method should be usable as a standalone technique for thermodynamic analyses.

The application of mathematical model functions instead of the classic van't Hoff equation for the fitting procedure appeared to be a valid strategy to reproduce also complex temperature dependencies of the thermodynamic parameters. However, the profiles should be discussed in a robust way and with respect to the error bands. An analytical pitfall of this method is over‐ or under‐parametrized fitting of the van't Hoff datapoints. Secondly, continuity gaps might occur in the fitting functions. Both can evolve from small deviations into large artifacts such as surplus extrema or zero crossings when the fitting curves are numerically differentiated to obtain enthalpies and heat capacities. Especially at the lower and upper limits of the datasets this can lead to unrealistic enthalpy and entropy extrema; hence, these regions should always be treated with caution and results should be analyzed in a robust way. The extension of the datasets with more datapoints would further enhance the reliability of the fitting functions, but also the analytical effort. Therefore, time and benefit balancing are required. Evaluating the reliability of experimentally determined enthalpies and entropies in general as well as detailing certain effects like the enthalpy‐entropy compensation or transduction (Fenley et al., 2012; Kurzbach et al., 2014) is a different question that would exceed the scope of this work.

Beyond some assumptions and error sources, the method also provides superior possibilities. Thermodynamics of different binding states can be analyzed independently and simultaneously in the same sample without the interferences of other enthalpy‐changing processes such as in ITC. Instead, additional information about system dynamics and polarity can be assessed, utilizing the high sensitivity and information content of CW EPR spectroscopy. Due to their smaller size, DOXYL spin labels may also show less interference with the binding process than bulkier and/or more hydrophobic fluorescence labels. The approach is versatile and easily transferrable to other systems, with variable perspectives. The application of EPR‐STAMP only requires the spin probing or labeling of one interaction partner and a detectable change or difference in rotational dynamics or in another EPR parameter such as hyperfine couplings that are sensitive to the environmental polarity or spin exchange broadening (through high local concentrations) to distinguish between different system (binding) states.

### 3.3 Comparative retrospective to the transport protein serum albumin

Previously, we analyzed the temperature‐dependent thermodynamics of the larger and predominantly α‐helix‐containing transport protein human serum albumin (HSA) with a similar approach (Reichenwallner et al., 2025). Similar to FABPs, two bound states of 16‐DSA appeared in EPR spectra, but the more dynamic state at room temperature was interpreted as a ligand in lower‐affinity binding pockets opposing high affinity sites with strong immobilization. Our interpretation for FABPs supposes loose attachment on the portal region or on a certain location at the protein surface instead, due to the limited space within the β‐barrel and due to the hyperfine couplings that indicate aqueous environment. Albumin changes its structure at higher temperatures since rotational decoupling of albumin domains was observed, going along with an increase in ligand dynamics and finally resulting in gel formation (Reichenwallner et al., 2025). Therefore, changes in protein dynamics were indirectly detected through the ligand dynamics. We did not observe similar effects here, potentially due to the more compact structure of FABPs. However, we would also not exclude gel formation of FABPs, possibly above the highest concentration regime we analyzed.

## 4 CONCLUSIONS

The most important conclusions from this study can be concisely summarized:

1. We established, applied and evaluated temperature‐dependent CW EPR spectroscopy as a unique, stand‐alone technique for spectroscopically analyzing the thermodynamics of multiple simultaneous interaction processes between transport proteins and ligands (STAMP approach).
2. We found strong indications that binding thermodynamics and genetic isoform diversification, that is, differences in primary structures of transport proteins are functionally coupled in nature, suggesting bio‐functional thermodynamics beyond simple amphiphilic effects leading to ligand association.
3. FABPs show differences in their temperature‐dependent, thermodynamic binding profiles depending on their amino acid sequence, the interacting ligand, and the protein concentration. Recurring trends and features were also observed, such as enthalpy–entropy compensation, exergonic minima of intermediate and total binding, balancing of enthalpic and entropic binding transitions at physiological temperatures, and multiple varying transition temperatures.
4. At physiological temperature, cytosolic concentrations and pH value, the general thermodynamic binding and release pathway seems to process via entropy‐driven attachment and subsequent enthalpy‐driven interconversion or direct strong binding. The resulting total binding can be enthalpy‐ or entropy‐driven depending on the system, that is, the concentrations and temperatures. Below and above physiological temperature the profiles show stronger differences especially regarding the enthalpy and entropy of the processes.
5. The concentration dependence of the binding thermodynamics indicates that from a thermodynamic point of view optimum working concentrations exist for water‐soluble transport proteins to fulfill their regular functions. This could be a possible connection to harmful over‐ or under‐expression of FABPs co‐appearing with various diseases (Furuhashi & Hotamisligil, 2008; Haunerland & Spener, 2004) and suggests a new thermodynamic basis and perspective for the emergence of FABP‐related diseases.
6. High sequence identities and hydropathies of FABP3/FABP4 might be correlated to their observed higher similarities in strong binding and interconversion. On the other hand, more similar and positively charged domains of FABP4/FABP5, as well as a possible functional adaptation, might be correlated to higher similarities in intermediate and total binding of this pair.

### 4.1 Which open questions regarding the FABP thermodynamics remain?

The nature or even the exact location(s) of the intermediately bound components, the changes during the active transport of ligands, possible interactions with membranes, and the connection of ligand binding states to protein conformational changes observed in other studies (Antúnez‐Argüelles & Robles‐Gómez, 2020; Matsuoka et al., 2015) remain partially unknown and necessitate further studies. By including pulsed EPR spectroscopic measurements into our EPR‐STAMP approach, more details about the binding states and transitions with respect to structural features could be generated in future. We already attempted a preliminary 4‐pulse double electron–electron resonance (DEER) measurement with FABP4/16‐DSA at Q‐Band frequencies. Although slight modulation of the DEER trace seemed to appear, the modulation/noise ratio was too low to extract any reliable distance distribution from the data. The weak modulation could arise from any combination of dipolar‐coupled radicals; it was not possible to distinguish between intra‐ and intermolecular distances. If we consider that the intermediately bound state is very dynamic, it seems to be rather unlikely that a reliable distance distribution could be obtained for this specific system.

The relatively high analytical effort and time consumption of the approach can be diminished by process automatization. So far, the manual EPR simulations of the entire temperature spectra series are the most time‐consuming and error‐prone part of the analytical procedure. An automatization of this step, for example, by automatic recognition of different components via neural networking, automatic fitting software, or by including principle component analysis (PCA) as we use it for the analysis of IR spectra (Arabi et al., 2018), would open the way to quick and automatized thermodynamic analyses and is planned for future studies.

Our STAMP approach has been initially created for core‐shell polymers, adapted for HSA and now optimized and extended for FABPs. The study opens the way to a successful transfer to manifold applications with other native or synthetic molecular transport systems. Whenever interaction processes are detectable via EPR spectroscopy it is in principle possible to apply the STAMP approach. Beyond fundamental biophysical basic research, this would be very interesting, especially for polymer science, biotechnology and biomimetics. Knowing the effective thermodynamics of binding and release of ligands at different temperatures might be crucial for the design, fine‐tuning and control of switchable molecular transporters, biosensors and molecular machines based on polymers or supramolecular assemblies. Optimizing enthalpic or entropic contributions of different interactions could be the key for advanced biotechnical applications such as switchable drug transport and bio‐functionalized polymers.

## 5 MATERIALS AND METHODS

### 5.1 Sample preparation

Human recombinant FABP3, FABP4 and FABP5 (Sigma Aldrich, purity ≥ 98%) were reconstituted in ultrapure water (Milli‐Q Advantage A10, Millipore SAS, Merck) and diluted into buffer solutions of 20 mM N‐(2‐Hydroxyethyl)piperazine‐N′‐(2‐ethanesulfonic acid) (HEPES) and 150 mM sodium chloride (Sigma Aldrich, purity ≥ 99.5%) at pH 7.5. 5‐DSA ammonium salt (Avanti Polar Lipids, Merck) was dissolved in HEPES/NaCl solution at pH 12, treated by ultrasonic for 10 min and then diluted into HEPES/NaCl solution at pH 7.5. 16‐DSA (free fatty acid, Sigma Aldrich) was dissolved in 0.1 M potassium hydroxide solution (Carl Roth GmbH & Co. KG, purity ≥85%) and diluted similarly. Samples for EPR measurements were prepared identically as described in detail in our previous work (Michler et al., 2024). All substances were used without further purification.

### 5.2 CW EPR spectroscopic measurements, analyses and simulations

CW EPR spectroscopic experiments were conducted on a benchtop spectrometer Magnettech MS‐5000 (Freiberg Instruments GmbH, now Bruker Biospin) at X‐band frequency (~9.4 GHz). Temperature sequences were programmed to measure field sweeps from 332.5 to 342.5 mT with a modulation amplitude of 0.1 mT and a modulation frequency of 100 kHz from −30 to 90°C in 10 or 5 K steps. For all temperatures, 10 individual scans with 60 s of scanning time were measured and summed up. The sum CW EPR spectra were plotted and analyzed in Matlab R2021b with the Easyspin package (easyspin‐6.0.0‐dev.43) (Stoll & Schweiger, 2006). EPR spectral simulations were done with the Easyspin function *Chili* for spectra in the slow‐motional regime as developed by Schneider and Freed (Schneider & Freed, 1989; Stoll & Schweiger, 2006). Three spectral components (1), (2) and (3) were simulated with varying proportions in the spectra. The error bars for simulated component proportions (Φ) and derived concentrations were estimated by using the root‐mean‐square deviations (RMSD) of the simulations as absolute errors (Φ ± RMSD %). The simulated parameters for all systems at 5, 40 and 80°C can be found in Tables S3‐S15.

### 5.3 ATR‐IR spectroscopy

Attenuated total reflection (ATR) Fourier transform infrared spectroscopy (FTIR) was conducted to determine the denaturation temperature of the FABPs by observing changes in the secondary structure which were mainly detected in the amide‐I band. 30 μL of FABPx/16‐DSA solution (200/20 μM) was placed onto the Si‐crystal plate. Temperature was controlled by a circulation water bath (HAAKE C25P thermostat). Temperature spectra series from 30 to 90°C with 2 K steps and 180 s equilibration time were measured in the range of 5000–800 cm^−1^ with 64 scans and 4 cm^−1^ resolution, using a Bruker Tensor 27 FT‐IR spectrometer equipped with a BioATRCell II and an LN‐MCT photovoltaic detector and the OPUS Data Collection Program (Bruker, Ettlingen, Germany). The empty ATR cell was used as a reference and H_2_O spectra were subtracted from each spectrum at the same temperature. Principal component analyses were done with home‐written Matlab scripts.

### 5.4 Determination of thermodynamic parameters

The thermodynamic analyses were conducted with OriginPro2019 and Matlab R2021b. The entire procedure with all relevant equations is shown in Figure S1. Simulated component proportions for all temperatures were transformed into components concentrations. From these, ln*K*_x_ values were calculated, plotted against *T* ^−1^ and suitable fitting curves were tested to find mathematical models for the van't Hoff curves. Optimum fit functions were found via *χ* ^2^‐comparison and artifact minimization in a trial‐and‐error procedure. The fitting curve expressions were inserted into home‐written Matlab scripts for numeric calculation and plotting of $\Delta G{{}^{\circ}}_{a}$, $\Delta H{{}^{\circ}}_{a}$, $\Delta S{{}^{\circ}}_{a}$, $\Delta G{{}^{\circ}}_{a}$ and $\Delta C{{}^{\circ}}_{p,a}$ as functions of the temperatures. Error bands of the thermodynamic plots were estimated from additional fit functions for the upper and lower errors in the van't Hoff plots which were calculated by Gaussian error propagation from the component concentration errors. Since systematic errors can be expected from the simulation process, this strategy is valid for our systems. Linear and non‐linear van't Hoff fitting was conducted with home‐written Matlab scripts.

### 5.5 Sequence alignment and similarity analysis

The amino acid sequences of FABP3, FABP4 and FABP5 were aligned with the software Clustal Omega (Multiple Sequence Alignment) (Madeira et al., 2024) and visualized with ESPript 3.0 (Robert & Gouet, 2014). Crystal structures were taken from (Matsuoka et al., 2015) (PDB: 4WBK) and (Armstrong et al., 2014) (PDB: 4LKP) and visualized with PyMol version 4.6.0. Non‐identical residues were detected in the triple alignment, partial sequences were extracted and their GRAVY values and charges were calculated via Protparam using the scale of Kyte and Doolittle (Gasteiger et al., 2003; Kyte & Doolittle, 1982).

*Supporting Information*: This article contains 1 PDF file (“Supporting Information_FABP_Thermodynamics_Protein Science”) as Supporting Information. It includes: supplementary text with thermodynamic equations and additional but not discussion‐relevant binding curve analyses, Figures S1–S36 with a scheme of the analytical strategy, additional CW EPR spectra and simulation series, additional binding curves, thermodynamic profiles, the illustration of the FABP sequence alignment, non‐linear van't Hoff fitting and IR‐spectroscopic data as well as Tables S1–S16 with the EPR simulation parameters, the results of bioinformatical FABP sequence analyses and fitting results.

## AUTHOR CONTRIBUTIONS

**Sebastian Michler:** Conceptualization; validation; visualization; investigation; data curation; writing – original draft. **Christian Schwieger:** Conceptualization; methodology; validation; writing – review and editing. **Florian Arndt Schöffmann:** Conceptualization; writing – review and editing. **Dariush Hinderberger:** Conceptualization; investigation; methodology; validation; visualization; writing – review and editing; software; project administration; supervision; resources; funding acquisition.

## CONFLICT OF INTEREST STATEMENT

The authors declare no conflicts of interest.

## Supporting information


**Figure S1.** Strategy of spectroscopic‐thermodynamic profiling (STAMP analysis). Blue arrows refer to the process from simulations to binding affinities, orange arrows refer to the derivation of thermodynamic parameters from the binding affinities. All depicted equations are separately given in the main text.
**Figure S2.** CW EPR spectra (blue) and simulations (red) of 20 μM 16‐DSA with 50 μM FABP3 at 0–90°C.
**Figure S3.** CW EPR spectra (blue) and simulations (red) of 20 μM 16‐DSA with 100 μM FABP3 at 0–90°C.
**Figure S4.** CW EPR spectra (blue) and simulations (red) of 20 μM 16‐DSA with 200 μM FABP3 at 0–90°C.
**Figure S5.** CW EPR spectra (blue) and simulations (red) of 20 μM 5‐DSA with 50 μM FABP3 at 0–90°C.
**Figure S6.** CW EPR spectra (blue) and simulations (red) of 20 μM 5‐DSA with 100 μM FABP3 at 0–90°C.
**Figure S7.** CW EPR spectra (blue) and simulations (red) of 20 μM 5‐DSA with 200 μM FABP3 at 0–60°C.
**Figure S8.** CW EPR spectra (blue) and simulations (red) of 20 μM 16‐DSA with 50 μM FABP4 at 5–90°C.
**Figure S9.** CW EPR spectra (blue) and simulations (red) of 20 μM 16‐DSA with 100 μM FABP4 at 5–90°C.
**Figure S10.** CW EPR spectra (blue) and simulations (red) of 20 μM 16‐DSA with 200 μM FABP4 at 5–90°C.
**Figure S11.** CW EPR spectra (blue) and simulations (red) of 20 μM 5‐DSA with 50 μM FABP4 at 0–90°C.
**Figure S12.** CW EPR spectra (blue) and simulations (red) of 20 μM 5‐DSA with 200 μM FABP4 at 0–90°C.
**Figure S13.** CW EPR spectra (blue) and simulations (red) of 20 μM 5‐DSA with 100 μM FABP4 at 0–90°C.
**Figure S14.** CW EPR spectra (blue) and simulations (red) of 20 μM 16‐DSA with 50 μM FABP5 at 0–90°C.
**Figure S15.** CW EPR spectra (blue) and simulations (red) of 20 μM 16‐DSA with 100 μM FABP5 at 0–90°C.
**Figure S16.** CW EPR spectra (blue) and simulations (red) of 20 μM 16‐DSA with 200 μM FABP5 at 0–80°C.
**Figure S17.** CW EPR spectra (blue) and simulations (red) of 20 μM 5‐DSA with 50 μM FABP5 at 0–80°C.
**Figure S18.** CW EPR spectra (blue) and simulations (red) of 20 μM 5‐DSA with 100 μM FABP5 at 0–80°C.
**Figure S19.** CW EPR spectra (blue) and simulations (red) of 20 μM 5‐DSA with 200 μM FABP5 at 0–90°C.
**Figure S20.** Temperature binding curves of 5‐DSA with 200 (A), 100 (C), 50 (E) μM FABP3 and 16‐DSA with 200 (B), 100 (D), 50 (F) μM FABP3. The guiding curves for 200 μM 16‐DSA, 100 μM 5‐DSA and 50 μM 5‐DSA were slightly changed compared to reference (Michler et al., 2024) for visual reasons.
**Figure S21.** Temperature binding curves of 5‐DSA with 200 (A), 100 (C), 50 (E) μM FABP4 and 16‐DSA with 200 (B), 100 (D), 50 (F) μM FABP4.
**Figure S22.** Temperature binding curves of 5‐DSA with 200 (A), 100 (C), 50 (E) μM FABP5 and 16‐DSA with 200 (B), 100 (D), 50 (F) μM FABP5. The guiding curves for 50 μM 5‐DSA were slightly changed compared to reference (Michler et al., 2024) for visual reasons.
**Figure S23.** FABP3 with 16‐DSA in the concentration regimes 50/20 μM, 100/20 μM and 200/20 μM. No curve is available for F‐S in the regime 100/20 μM since always either [F] or [S] were equal to zero in the measured temperature range.
**Figure S24.** FABP3 with 5‐DSA in the concentration regimes 50/20 μM, 100/20 μM and 200/20 μM. No curves are available for F‐I, F‐S and F‐T of the system 200/20 μM since [F] was equal to zero in the entire temperature range.
**Figure S25.** FABP4 with 16‐DSA in the concentration regimes 50/20 μM, 100/20 μM and 200/20 μM.
**Figure S26.** FABP4 with 5‐DSA in the concentration regimes 50/20 μM, 100/20 μM and 200/20 μM. No curves are available for F‐S and I‐S in the system 50/20 μM since [S] is equal to 0 μM in the entire temperature range.
**Figure S27.** FABP5 with 16‐DSA in the concentration regimes 50/20 μM, 100/20 μM and 200/20 μM.
**Figure S28.** FABP5 with 5‐DSA in the concentration regimes 50/20 μM, 100/20 μM and 200/20 μM.
**Figure S29.** FABP3/4/5 50 μM with 16‐DSA 20 μM.
**Figure S30.** Amino acid sequence alignment and sequence similarities of FABP5, FABP3 and FABP4. Top: Alignment of all three FABPs, colored by similarity with: white font in red fields = identical amino acids, black font in yellow fields = similar properties of amino acids, black font in white fields = no similarity. Bottom: Crystal structure of FABP5 (Armstrong et al., 2014) with identical (red) and divergent (white) amino acids from the alignment of all three FABPs. The crystal structure is shown from two different orientations, without and with amino acid residues as sticks.
**Figure S31.** FABP5 crystal structure from two perspectives with colored domains of highest diversity among the three FABPs. (Armstrong et al., 2014) α2 (red) is most hydrophobic for FABP5 (red), β2‐TT (magenta) is most hydrophobic for FABP4, β4‐TT (blue) and β6‐TT (green) are most hydrophilic for FABP5, β8‐TT (yellow) and β9‐TT‐β10 (orange) are in the medium range for FABP5.
**Figure S32.** Non‐linear van't Hoff fitting of the data from FABP3 with 5/16‐DSA. Non‐linear fitting curves are drawn as solid lines, classic (linear) ones as dashed lines.
**Figure S33.** Non‐linear van't Hoff fitting of the data from FABP4 with 5/16‐DSA. Non‐linear fitting curves are drawn as solid lines, classic (linear) ones as dashed lines.
**Figure S34.** Non‐linear van't Hoff fitting of the data from FABP5 with 5/16‐DSA. Non‐linear fitting curves are drawn as solid lines, classic (linear) ones as dashed lines.
**Figure S35.** Results of the principal component analysis applied on the amide I band of ATR‐IR spectra from FABP3/4/5 with 16‐DSA. (A) FABP3. (B) FABP4. (C) FABP5.
**Figure S36.** Example profile for FABP3/16‐DSA 50/20 μM generated from the non‐linearly fitted van't Hoff curves. The profile generated by our model fits is shown on the right side for comparison. Dashed fits in the enthalpy profile were calculated via differentiation.
**Table S1.** Van't Hoff curve fitting functions for all systems and transitions. PolyX stands for polynomial fitting function of the Xth order.
**Table S2.** Comparison of hydropathicity and charge for non‐identical residues of selected structural FABP domains after triple alignment. Structural domains with the strongest sequence deviations and the lowest similarities were analyzed via Protparam. The net charges and GRAVY values were extracted, highest GRAVY values are marked in red, lowest in blue for each structure domain. In the bottom lines, the values for the complete sequences of each FABP are given for comparison. ^#^GRAVY = grand average of hydropathy − sum of hydropathy values divided by sequence length; increasing positive score = greater hydrophobicity. *The selected FABP shows stronger sequence deviation in this domain than the other two FABPs.
**Table S3.** Simulation parameters of free 5/16‐DSA.
**Table S4.** Simulation parameters of intermediately bound FABP3/16‐DSA.
**Table S5.** Simulation parameters of intermediately bound FABP3/5‐DSA.
**Table S6.** Simulation parameters of strongly bound FABP3/16‐DSA.
**Table S7.** Simulation parameters of strongly bound FABP3/5‐DSA.
**Table S8.** Simulation parameters of intermediately bound FABP4/16‐DSA.
**Table S9.** Simulation parameters of intermediately bound FABP4/5‐DSA.
**Table S10.** Simulation parameters of strongly bound FABP4/16‐DSA.
**Table S11.** Simulation parameters of strongly bound FABP4/5‐DSA.
**Table S12.** Simulation parameters of intermediately bound FABP5/16‐DSA.
**Table S13.** Simulation parameters of intermediately bound FABP5/5‐DSA.
**Table S14.** Simulation parameters of strongly bound FABP5/16‐DSA.
**Table S15.** Simulation parameters of strongly bound FABP5/5‐DSA.
**Table S16.** Results of linear (L) and non‐linear (NL) van't Hoff fits for selected samples.

## Data Availability

The data that support the findings of this study are available from the corresponding author upon reasonable request.
