# Supplementary material for: Functional thermodynamics govern the ligand binding to human cytosolic transport proteins
Source: Protein Sci. 2026 May 25;35(6):e70637. doi: 10.1002/pro.70637 (PMC13239702; doi:10.1002/pro.70637)
Supplement: Supplementary file 1 — Figure S1. Strategy of spectroscopic‐thermodynamic profiling (STAMP analysis). Blue arrows refer to the process from simulations to binding affinities, orange arrows refer to the derivation of thermodynamic parameters from the binding affinities. All depicted equations are separately given in the main text. Figure S2. CW EPR spectra (blue) and simulations (red) of 20 μM 16‐DSA with 50 μM FABP3 at 0–90°C. Figure S3. CW EPR spectra (blue) and simulations (red) of 20 μM 16‐DSA with 100 μM FABP3 at 0–90°C. Figure S4. CW EPR spectra (blue) and simulations (red) of 20 μM 16‐DSA with 200 μM FABP3 at 0–90°C. Figure S5. CW EPR spectra (blue) and simulations (red) of 20 μM 5‐DSA with 50 μM FABP3 at 0–90°C. Figure S6. CW EPR spectra (blue) and simulations (red) of 20 μM 5‐DSA with 100 μM FABP3 at 0–90°C. Figure S7. CW EPR spectra (blue) and simulations (red) of 20 μM 5‐DSA with 200 μM FABP3 at 0–60°C. Figure S8. CW EPR spectra (blue) and simulations (red) of 20 μM 16‐DSA with 50 μM FABP4 at 5–90°C. Figure S9. CW EPR spectra (blue) and simulations (red) of 20 μM 16‐DSA with 100 μM FABP4 at 5–90°C. Figure S10. CW EPR spectra (blue) and simulations (red) of 20 μM 16‐DSA with 200 μM FABP4 at 5–90°C. Figure S11. CW EPR spectra (blue) and simulations (red) of 20 μM 5‐DSA with 50 μM FABP4 at 0–90°C. Figure S12. CW EPR spectra (blue) and simulations (red) of 20 μM 5‐DSA with 200 μM FABP4 at 0–90°C. Figure S13. CW EPR spectra (blue) and simulations (red) of 20 μM 5‐DSA with 100 μM FABP4 at 0–90°C. Figure S14. CW EPR spectra (blue) and simulations (red) of 20 μM 16‐DSA with 50 μM FABP5 at 0–90°C. Figure S15. CW EPR spectra (blue) and simulations (red) of 20 μM 16‐DSA with 100 μM FABP5 at 0–90°C. Figure S16. CW EPR spectra (blue) and simulations (red) of 20 μM 16‐DSA with 200 μM FABP5 at 0–80°C. Figure S17. CW EPR spectra (blue) and simulations (red) of 20 μM 5‐DSA with 50 μM FABP5 at 0–80°C. Figure S18. CW EPR spectra (blue) and simulations (red) of 20 μM 5‐DSA with 100 μM FABP5 at 0–80 [file PRO-35-e70637-s001.pdf]

Supplementary materials

Functional thermodynamics govern the ligand binding to human cytosolic transport proteins

Sebastian Michler, [1] Christian Schwieger, [1] Florian Arndt Schöffmann, [1] Dariush Hinderberger, [1*]

[1]Martin Luther University Halle-Wittenberg, Institute of Chemistry, Physical Chemistry – Complex Self-Organizing Systems, Von-Danckelmann-Platz 4, 06120, Halle (Saale), Germany

*Dariush Hinderberger, dariush.hinderberger@chemie.uni-halle.de

This PDF file includes:

1. Supplementary Text

2. Figures S1 to S36

    2.1 Overview of the method strategy

    2.2 Temperature series of CW EPR spectra with simulations

    2.3 Temperature-dependent binding curves

    2.4 Thermodynamic profiles

    2.5 Additional comparison of functional thermodynamics between FABPs

    2.6 Sequence similarities of FABP3, FABP4 and FABP5

    2.7 Linear and non-linear van't Hoff fitting

    2.8 ATR-IR spectroscopy on FABPs to determine their denaturation temperature

    2.9 Application of the classic non-linear van't Hoff fitting on an example system

3. Tables S1 to S16

    3.1 Model fit functions for van't Hoff plots

    3.2 Amino acid sequence parameters

    3.3 Parameters of the EPR simulations

    3.4 Results of linear/non-linear van't Hoff fitting for selected cases

1. Supplementary Text

Definitions for binding equations

$[PL]_{e,X}$ = Concentration of protein-ligand complexes in equilibrium, with $X \in (I, S, T)$

$[P_F]_e$ = Concentration of free proteins in equilibrium

$[P_I]_e$ = Concentration of proteins with intermediately bound ligands in equilibrium

$[P_S]_e$ = Concentration of proteins with strongly bound ligands in equilibrium

$[P_T]_e$ = Concentration of proteins with all bound ligands in equilibrium

$[L_F]_e$ = Concentration of free ligands in equilibrium

Φ_{X} = simulated proportion of a component, with $X \in (I, S, T, F)$

$[X]$ = simulated ligand concentration of a component in equilibrium, with $X \in (I, S, T, F)$

$[P_t]$ = total protein concentration, with $[P_t] \in (50, 100, 200\ \mu\mathrm{M})$

$[L_t]$ = total ligand concentration, with $[L_t] = 20\ \mu\mathrm{M}$

General equations

$$K_A = \frac{[PL]_{e,X}}{[P_F]_e \cdot [L_F]_e} \quad (1)$$

$$[X] = \frac{\Phi_{\mathrm{X}}}{100} \cdot [L_t] \quad (2)$$

Equations for transitions F-I and F-S

There are two cases distinguishable for simultaneous intermediate and strong binding of ligands in a coupled equilibrium:

Case 1) $[P_I]_e \neq [P_S]_e$ If the concentrations of proteins with intermediately bound and strongly bound ligands can be different, this means that the two binding states may appear at different protein molecules or together, but each bind to the FABPs always in the molar ratio 1:1,

then: $$[P_T]_e = [P_I]_e + [P_S]_e, \quad (3)$$

and: $[P_t] = [P_T]_e + [P_F]_e.$ (4)

Case 2) $[P_I]_e = [P_S]_e$ If the concentrations of proteins with intermediately and strongly bound ligands are the same and equal to the total concentration of proteins with bound ligands, this means that intermediately and strongly bound ligands appear only together on the same protein molecules and bind always in the molar ratio 1:1,

then: $[P_T]_e = [P_I]_e = [P_S]_e,$ (5)

and $[P_t]$ is calculated via eq. (4) as in case 1).

In both cases the free ligand concentration in equilibrium has to be calculated concerning both binding states:

$$[L_F]_e = [L_t] - [T] = [L_t] - [I] - [S] \quad (6)$$

In summary, for the free equilibrium concentrations of protein and ligand we can define either:

$[P_F]_{e,1} = [P_t] - [P_I]_e - [P_S]_e$ (case 1) or, (7)

$[P_F]_{e,2} = [P_t] - [P_{I=S}]_e$ (case 2), (8)

while eq. (6) is always applicable for the ligand concentration. Concerning 1:1 binding of ligands and proteins, we can further define for the complex in equilibrium:

$[PL]_{e,X} = [X]$, with $X \in (I, S, T)$ (9)

We can now insert eqs. (6), (7) **or** (8) and (9) into the equilibrium eq. (1). We will show **case 1** for simultaneous binding transitions F-I and F-S at first, starting with the focus on F-I.

$$K_{F-I} = \frac{[PL]_{e,I}}{[P_F]_{e,1} \cdot [L_F]_e} = \frac{[I]}{[P_F]_{e,1} \cdot [L_F]_e} = \frac{[I]}{([P_t] - [P_I]_e - [P_S]_e) \cdot ([L_t] - [I] - [S])}$$

$$K_{F-I} = \frac{[I]}{([P_t] - [I] - [S]) \cdot ([L_t] - [I] - [S])}$$
$$= \frac{[I]}{([P_t][L_t]) - ([P_t][I]) - ([P_t][S]) - ([L_t][I]) + [I]^2 + ([I][S]) - ([L_t][S]) + ([I][S]) + [S]^2}$$

$$K_{F-I} = \frac{[I]}{([P_t][L_t]) - ([P_t][I]) - ([P_t][S]) - ([L_t][I]) + [I]^2 + (2\,[I][S]) - ([L_t][S]) + [S]^2}$$

Each part of the ratio can now be divided by $[I]$ to simplify this expression.

$$K_{F-I} = \frac{1}{\left(\frac{[P_t][L_t]}{[I]} - [P_t] - \frac{[P_t][S]}{[I]}\right) - [L_t] + [I] + 2\,[S] - \left(\frac{[L_t][S]}{[I]}\right) + \left(\frac{[S]^2}{[I]}\right)}$$

$$K_{F-I} = \frac{1}{[P_t]\left(\frac{[L_t]}{[I]} - 1 - \frac{[S]}{[I]}\right) - ([L_t] - [I] - [S]) + \ [S] - \left(\frac{[L_t][S]}{[I]}\right) + \left(\frac{[S]^2}{[I]}\right)}$$

By replacing $[L_t] - [I] - [S]$ with $[F]$ this leads to the following final equation:

$$K_{F-I} = \frac{1}{[P_t]\left(\frac{[L_t]}{[I]} - 1 - \frac{[S]}{[I]}\right) - [F] + [S] - \frac{[L_t][S]}{[I]} + \frac{[S]^2}{[I]}} \qquad (10)$$

For cases in which the additional bound component becomes S = 0, the expression simplifies to:

$$K_{F-I} = \frac{1}{[P_t]\left(\frac{[L_t]}{[I]} - 1\right) - [F]} \qquad (11)$$

If we focus on the transition F-S, the equations stay similar except for $[I]$ and $[S]$ exchanging positions:

$$K_{F-S} = \frac{1}{[P_t]\left(\frac{[L_t]}{[S]} - 1 - \frac{[I]}{[S]}\right) - [F] + \ [I] - \frac{[L_t][I]}{[S]} + \frac{[I]^2}{[S]}} \qquad (12)$$

And for I = 0 as before,

$$K_{F-S} = \frac{1}{[P_t]\left(\frac{[L_t]}{[S]} - 1\right) - [F]} \qquad (13)$$

For **case 2** we have to use eq. (8) instead of (7) and repeat the derivation, again with transition F-I as example:

$$K_{F-I} = \frac{[I]}{[P_F]_{e,1} \ \cdot \ [L_F]_e} = \frac{[I]}{([P_t] - [P_I]_e) \ \cdot \ ([L_t] - [I] - [S])}$$
$$= \frac{[I]}{([P_t] - [I]) \ \cdot ([L_t] - [I] - [S])}$$

$$K_{F-I} = \frac{[I]}{([P_t][L_t]) - ([P_t][I]) - ([P_t][S]) - ([L_t][I]) + [I]^2 + ([I][S])}$$

$$K_{F-I} = \frac{1}{\left(\frac{[P_t][L_t]}{[I]} - [P_t] - \frac{[P_t][S]}{[I]}\right) - [L_t] + [I] + [S]}$$

$$K_{F-I} = \frac{1}{\left(\frac{[P_t][L_t]}{[I]} - [P_t] - \frac{[P_t][S]}{[I]}\right) - ([L_t] - [I] - [S])}$$

This leads to the following final equation for case 2:

$$K_{F-I} = \frac{1}{[P_t]\left(\frac{[L_t]}{[I]} - 1 - \frac{[S]}{[I]}\right) - [F]} \quad (14)$$

And for the transition F-S similar as before:

$$K_{F-S} = \frac{1}{[P_t]\left(\frac{[L_t]}{[S]} - 1 - \frac{[I]}{[S]}\right) - [F]} \quad (15)$$

If the second bound component becomes 0, then eqs. (14) and (15) simplify to (11) and (13) like for case 1.

Equations for transition F-T

We can also consider the case of total binding with the transition F-T:

$$K_{F-T} = \frac{[T]}{[P_F]_{e,1} \cdot [L_F]_e} \quad (16)$$

Since there is only one bound component appearing, eq. (8) simplifies to $[P_F]_{e,2} = [P_t] - [T]$ and (6) to $[L_F]_e = [L_t] - [T]$. With the same procedure as before we obtain an equivalent of eq. (13):

$$K_{F-T} = \frac{1}{[P_t]\left(\frac{[L_t]}{[T]} - 1\right) - [F]} \quad (17)$$

Equations for transition I-S

Finally, the transition I-S which can be described as a dynamic interconversion can be also considered:

$$K_{I-S} = \frac{[S]}{[I]} \quad (18)$$

The expression becomes simple, since the component proportions can be directly inserted in this case:

$$K_{I-S} = \frac{\Phi_S}{\Phi_I} \quad (19)$$

From eqs. 10, 12, 14, 15 it can be derived directly that K_{F-I} and K_{F-S} approach infinite values or become negative when no free ligands were detected via EPR and thereby [F] = 0 µM. The corresponding lnK_a values are either extremely high or not defined, in both cases they are excluded from the graph, leading to simplification of the thermodynamic profiles. This case appears drastically e.g. for the systems FABP3/16-DSA 100/20, 200/20 and FABP3/5-DSA 200/20.

Supplementary, not discussed analysis to the main chapter '*From CW EPR simulations to thermodynamic parameters*'

F-T shows no heat capacity difference until 46 °C. $\Delta C°_{\mathrm{p,\,F-T}}$ above 46 °C is negative with a minimum of at 53 °C, but it becomes positive again above 60 °C with a maximum at 67 °C.

Supplementary, not discussed analysis to the main chapter '*Binding thermodynamics depend on the FABP concentration regime*'

While the zero transition of the entropy differences remains almost the same for F-I when going from M- to H-regime in the system FABP3/16-DSA, it changes much stronger for F-T, while it is relatively similar between L and M. Furthermore, the enthalpy differences in the H-regime decrease when approaching the lowest and highest temperatures and do not approach 0 kJ/mol anymore. The enthalpy and entropy differences of I-S do not decrease linearly anymore in the M-regime, but more steeply, rather xth-order polynomially or even exponentially. In the H-regime they initially increase until 29 °C and then decrease again. Enthalpy and entropy of I-S decrease at low temperatures and increase at high temperatures for rising FABP concentration.

A FABP5 concentration in the H-regime leads to less exergonic F-T and F-I transitions, a more exergonic I-S transition and a less exergonic F-S transition until 50 °C but a more exergonic transition above this temperature (see **Figure S27**).

Supplementary, not discussed analysis to the main chapter '*The functional thermodynamics of FABP3, FABP4 and FABP5 in comparison*'

Intermediate binding of 16-DSA to FABP5 in the L-regime has a $\Delta G°_{\mathrm{F-I}}$ minimum of -31 kJ/mol at 46 °C. It is endergonic until 30 °C and exergonic thereafter, with an enthalpy minimum at 66 °C. At the same temperature it changes from negative to positive heat capacity differences. F-I becomes entropically non-favored at 46 °C, much later than in the case of FABP3. At 81-85 °C, F-I becomes endergonic but entropically favored again. Like for FABP3, total binding is

slightly more exergonic than F-I at lower temperatures and has a more negative enthalpy difference. F-T has its $\Delta G°_{F-T}$ minimum of -31 kJ/mol at 41 °C, it becomes exothermic already at 15 °C, has enthalpy/entropy minima identical to F-I, but it stays exothermic in the entire range. It is entropically favored until 41 °C and becomes unfavored thereafter, the heat capacity differences are almost identical to those in F-I. The transition F-S is exergonic, but $\Delta G°_{F-S}$ increases less than in the case of FABP3. It is completely exothermic here, with a $\Delta H°_{F-S}$ maximum at 12 °C and strongly decreasing enthalpy differences until 40 °C. F-S is entropically favored until 23 °C and unfavored above, and it changes from positive to negative heat capacity difference at 11 °C. I-S becomes endergonic at 7 °C but rises only up to 5 kJ/mol and becomes not as endergonic as for FABP3. It has a lower conversion enthalpy than F-S, but only at low temperature. It is exothermic, with a maximum at 17 °C, entropically unfavored and changes from positive to negative heat capacity difference at 16 °C.

Total binding of 16-DSA to FABP5 in the M-regime is endothermic until 19 °C, above which it remains exothermic until 90 °C. Intermediate binding is endothermic below 32 °C and then exothermic until 90 °C, too. Both transitions show negative $\Delta C°_p$ from 0-90 °C, although total binding reaches $\Delta C°_p = 0$ at 84 °C. Furthermore, both show decaying entropies with positive-negative-transitions at 54 °C and 45 °C for F-I and F-T, respectively. F-S and I-S are exergonic in the low temperature area and become endergonic at 40 °C.

In some systems $\Delta G°_{I-S}$ has a second maximum which is not located at the highest temperature and only FABP5/5-DSA has two $\Delta G°_{I-S}$ zero crossings in the H-regime. The I-S transition from exergonic to endergonic character is shifted to higher temperatures with rising protein concentration for FABP3 with 5/16-DSA, but only partially for the FABP4 and FABP5 systems. FABP3 and also partially FABP4 show a $\Delta G°_{F-I}$ maximum at around 40 °C, other FABP4 and FABP5-based systems (concentration dependent) show this maximum rather at around 70 °C. At 70 °C, frequently entropy zero crossings occur for F-I and sometimes also for F-T. As an example, one enthalpy zero crossing of F-I occurs at 30-40 °C as discussed before for the first example system. Enthalpy, entropy and heat capacity zero transitions of F-I, F-S and I-S often co-appear at the same temperature. In case of FABP5, especially with 5-DSA in the H-regime, zero crossings of different transitions often match together at similar temperatures showing effective connections between different transitions and compensations of enthalpy and entropy. For FABP3 enthalpy and entropy minima mostly appear at 50-60 °C.

2. Supplementary Figures

2.1 Overview of the method strategy

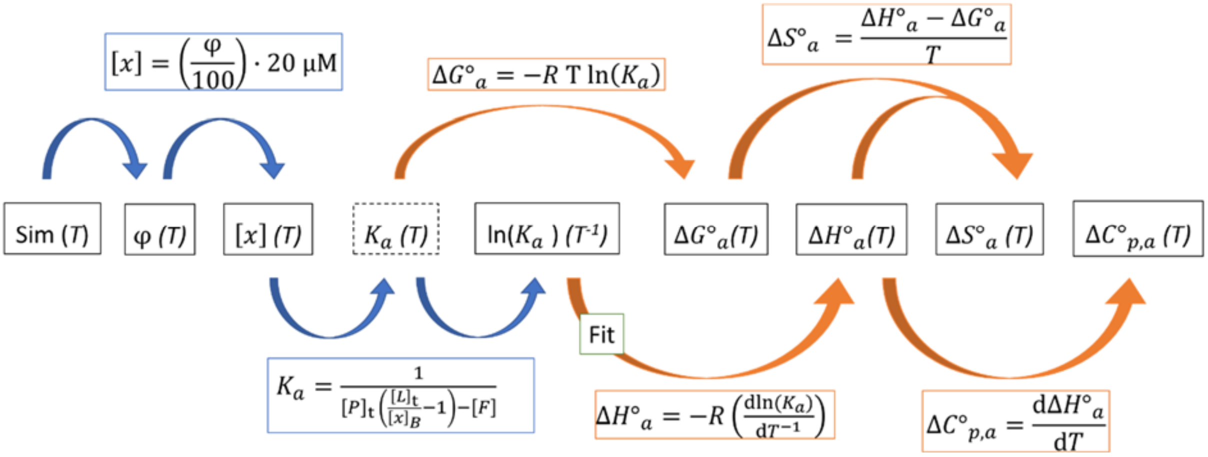

Figure S1. Strategy of spectroscopic-thermodynamic profiling (STAMP analysis). Blue arrows refer to the process from simulations to binding affinities, orange arrows refer to the derivation of thermodynamic parameters from the binding affinities. All depicted equations are separately given in the main text.

2.2 Temperature series of CW EPR spectra with simulations

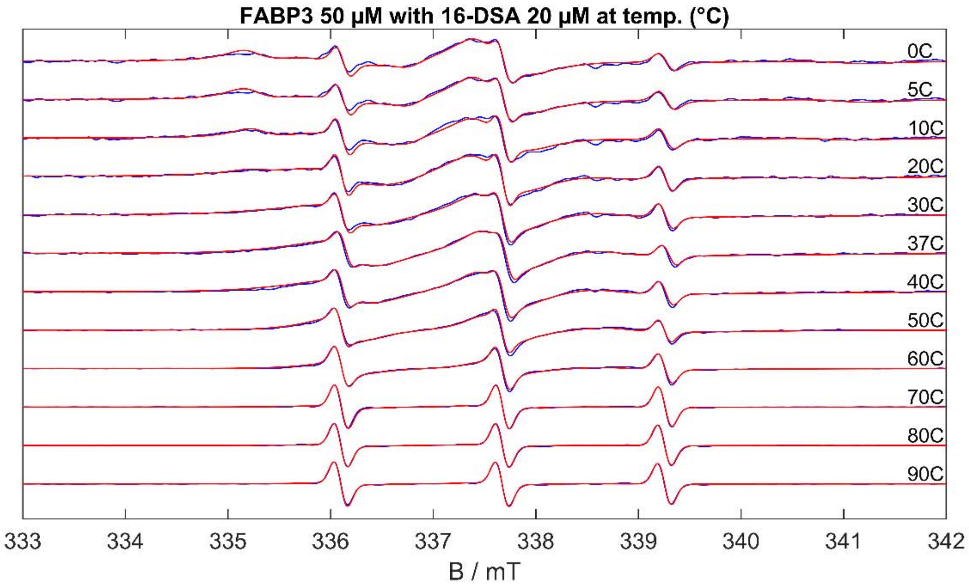

Figure S2. CW EPR spectra (blue) and simulations (red) of 20 μM 16-DSA with 50 μM FABP3 at 0-90 °C.

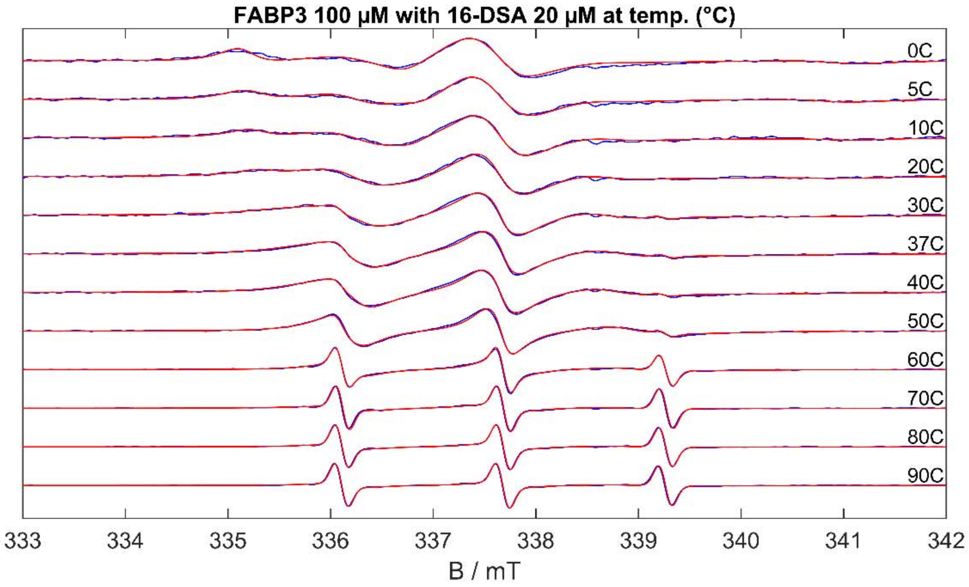

Figure S3. CW EPR spectra (blue) and simulations (red) of 20 µM 16-DSA with 100 µM FABP3 at 0-90 °C.

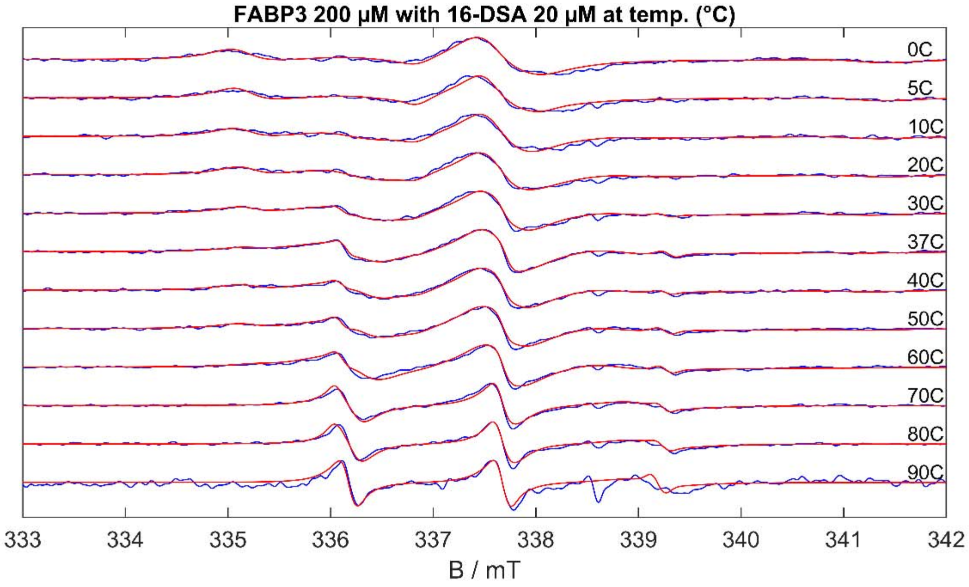

Figure S4. CW EPR spectra (blue) and simulations (red) of 20 µM 16-DSA with 200 µM FABP3 at 0-90 °C.

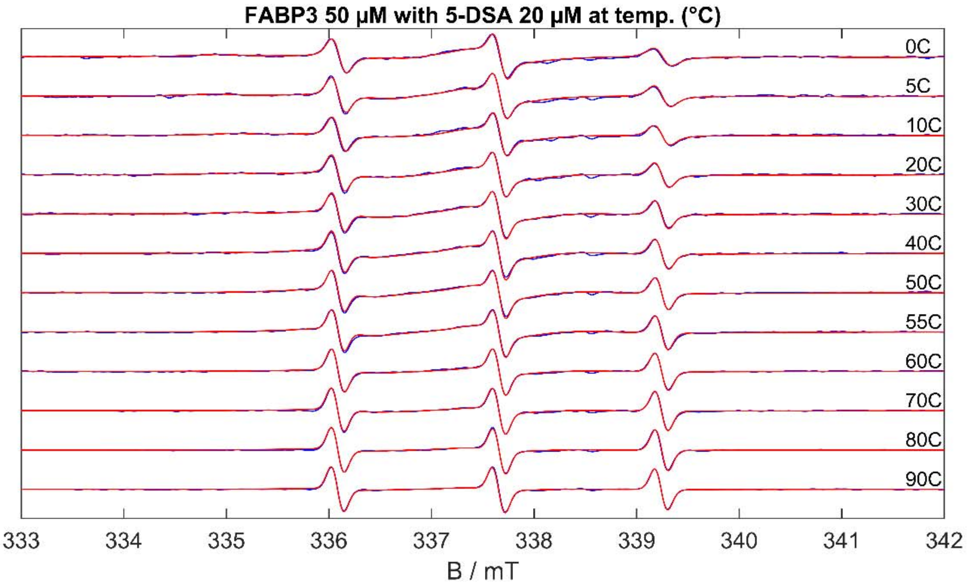

Figure S5. CW EPR spectra (blue) and simulations (red) of 20 µM 5-DSA with 50 µM FABP3 at 0-90 °C.

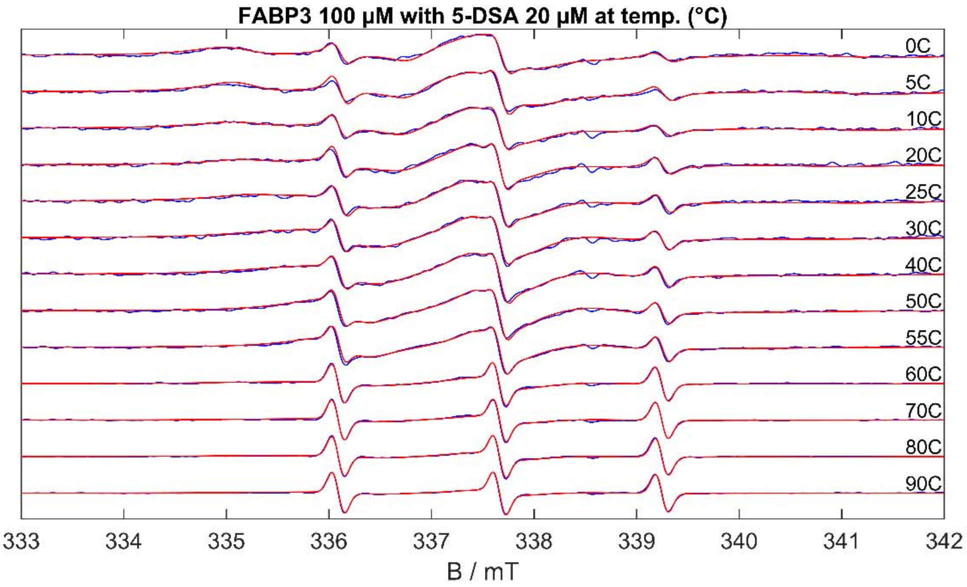

Figure S6. CW EPR spectra (blue) and simulations (red) of 20 µM 5-DSA with 100 µM FABP3 at 0-90 °C.

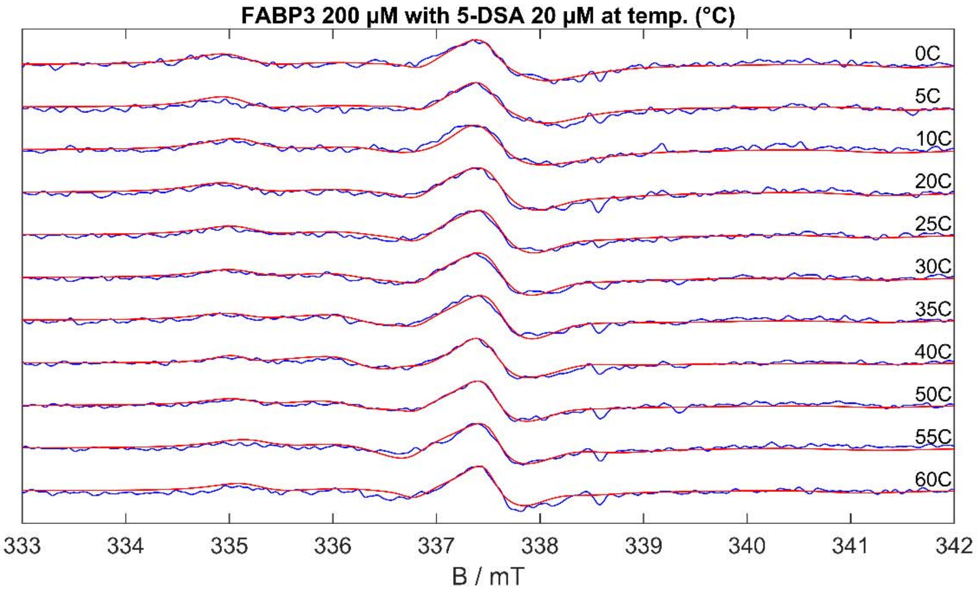

Figure S7. CW EPR spectra (blue) and simulations (red) of 20 μM 5-DSA with 200 μM FABP3 at 0-60 °C.

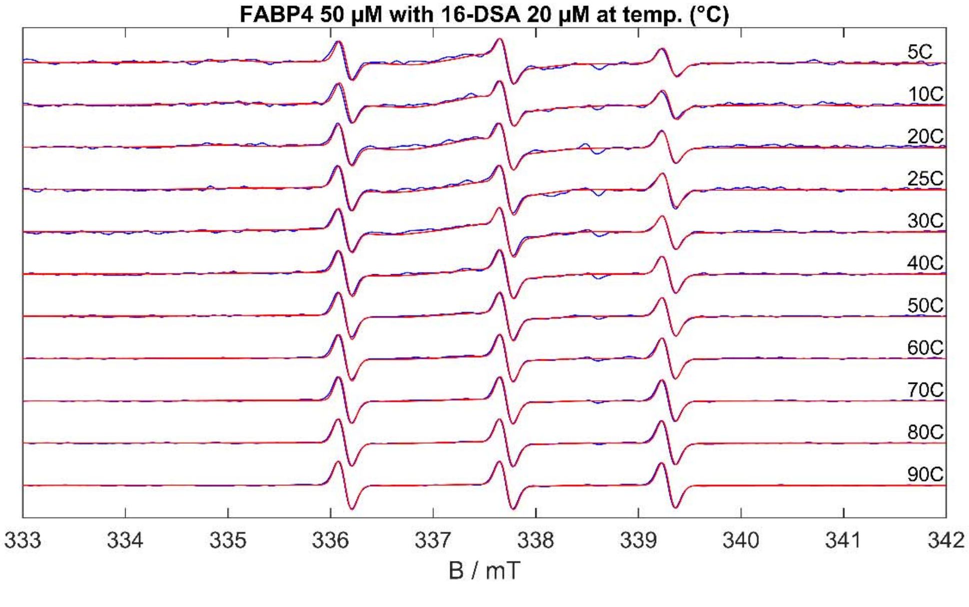

Figure S8. CW EPR spectra (blue) and simulations (red) of 20 μM 16-DSA with 50 μM FABP4 at 5-90 °C.

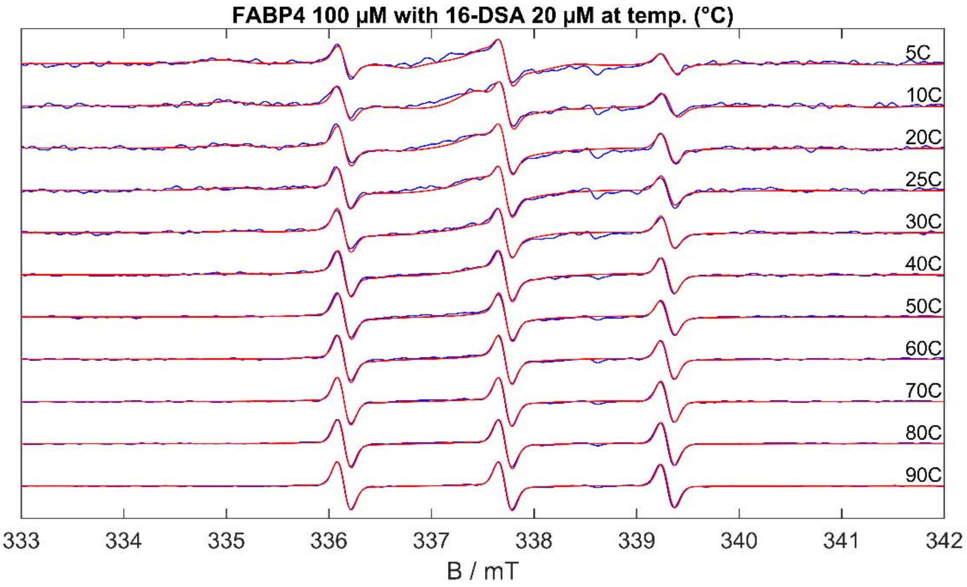

Figure S9: CW EPR spectra (blue) and simulations (red) of 20 µM 16-DSA with 100 µM FABP4 at 5-90 °C.

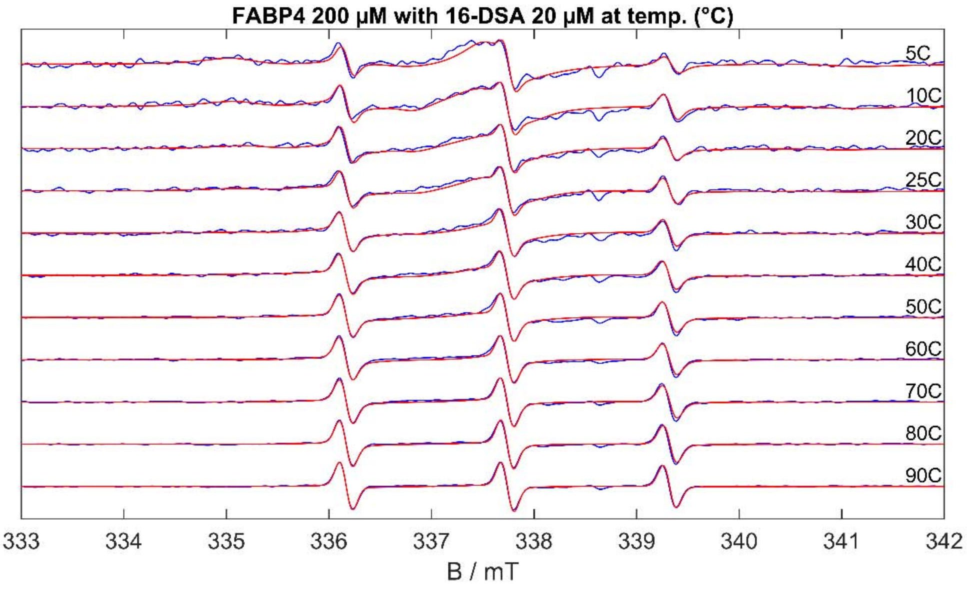

Figure S10. CW EPR spectra (blue) and simulations (red) of 20 µM 16-DSA with 200 µM FABP4 at 5-90 °C.

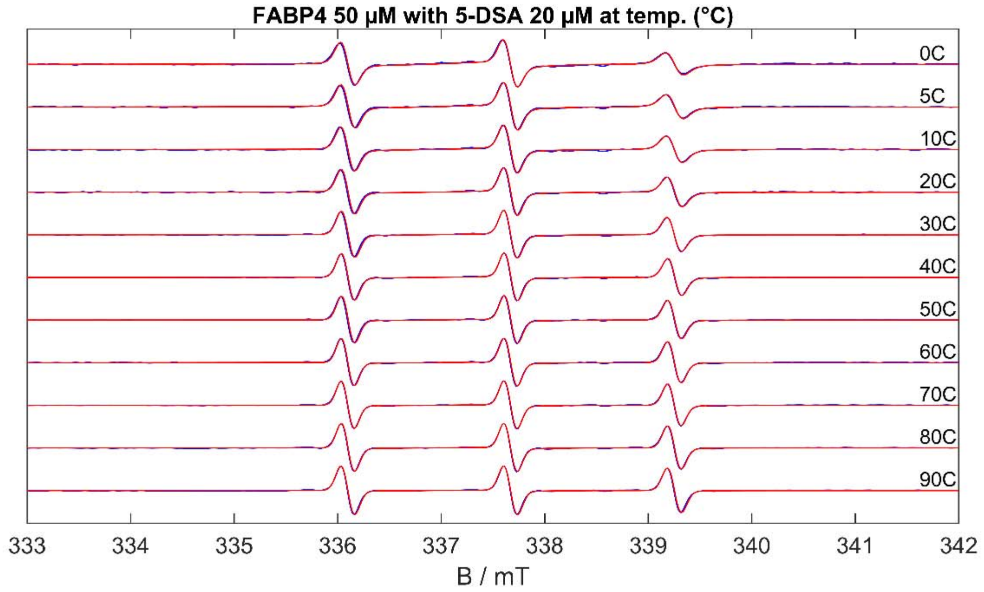

Figure S11. CW EPR spectra (blue) and simulations (red) of 20 µM 5-DSA with 50 µM FABP4 at 0-90 °C.

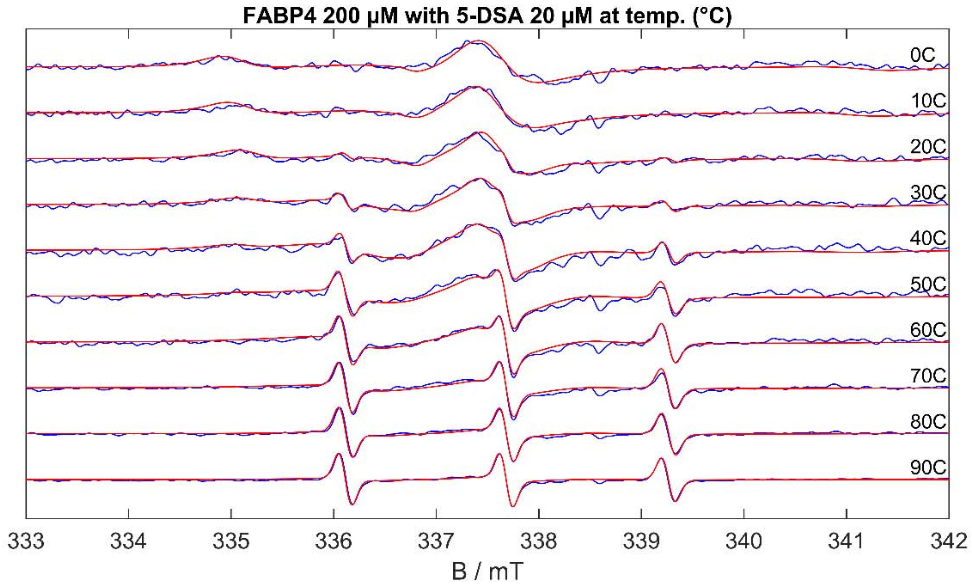

Figure S12. CW EPR spectra (blue) and simulations (red) of 20 µM 5-DSA with 200 µM FABP4 at 0-90 °C.

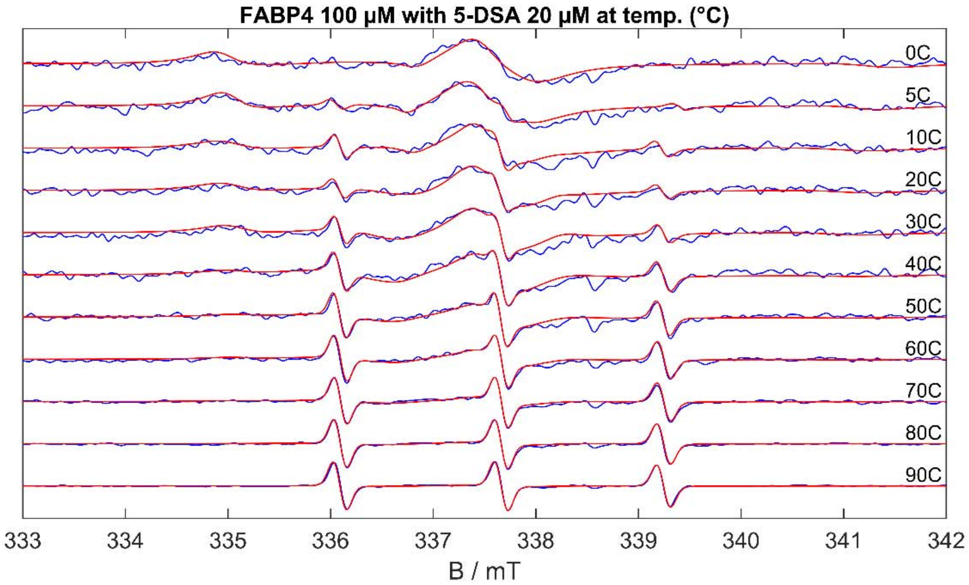

Figure S13. CW EPR spectra (blue) and simulations (red) of 20 µM 5-DSA with 100 µM FABP4 at 0-90 °C.

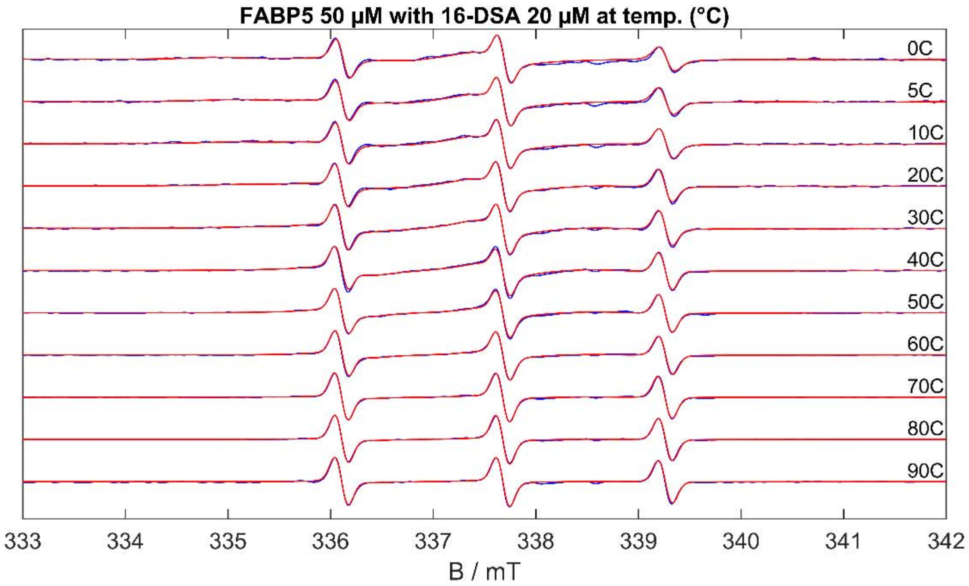

Figure S14. CW EPR spectra (blue) and simulations (red) of 20 µM 16-DSA with 50 µM FABP5 at 0-90 °C.

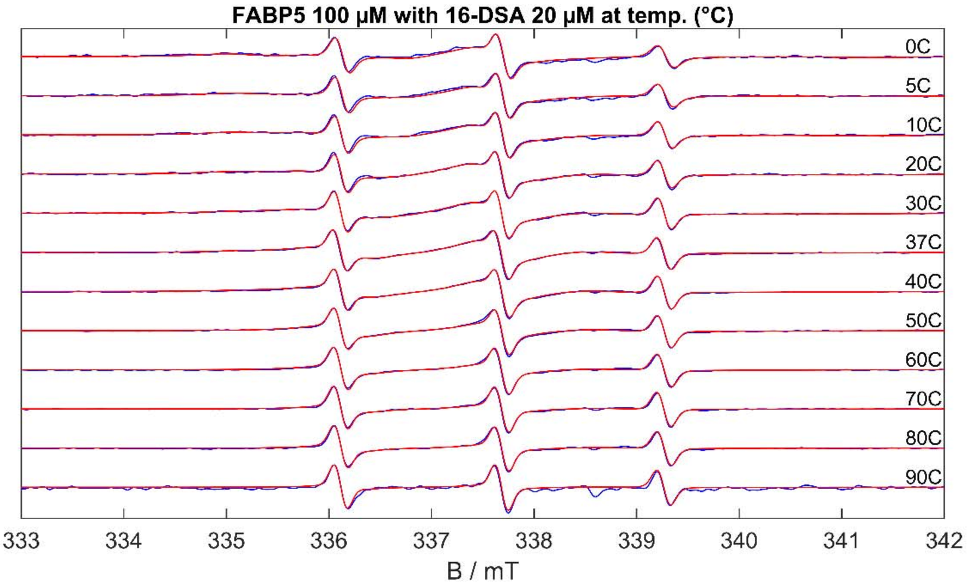

Figure S15. CW EPR spectra (blue) and simulations (red) of 20 µM 16-DSA with 100 µM FABP5 at 0-90 °C.

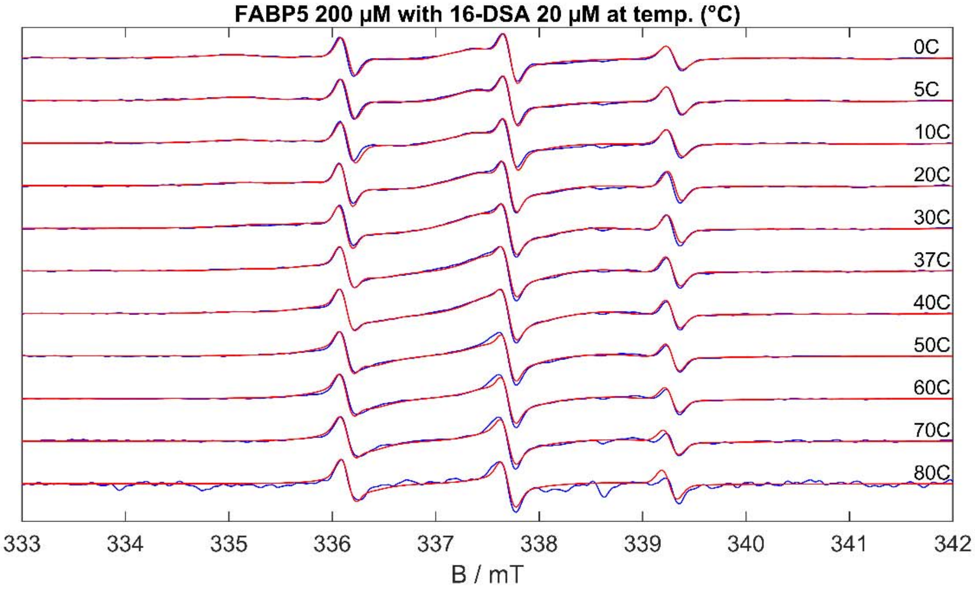

Figure S16. CW EPR spectra (blue) and simulations (red) of 20 µM 16-DSA with 200 µM FABP5 at 0-80 °C.

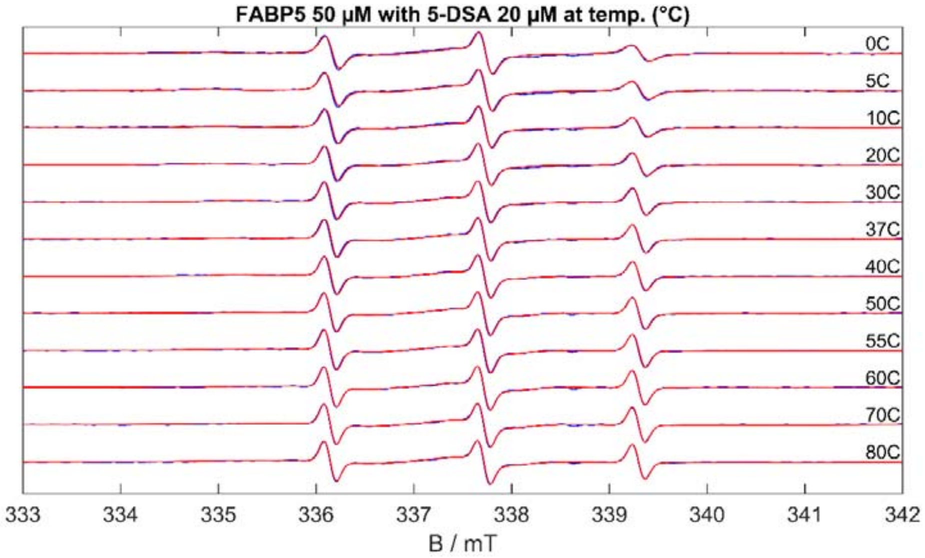

Figure S17. CW EPR spectra (blue) and simulations (red) of 20 μM 5-DSA with 50 μM FABP5 at 0-80 °C.

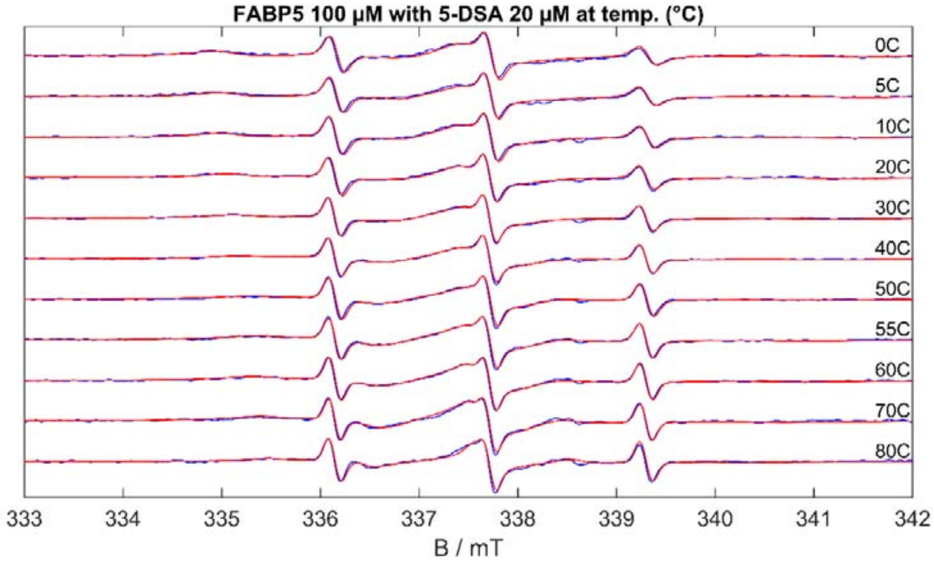

Figure S18. CW EPR spectra (blue) and simulations (red) of 20 μM 5-DSA with 100 μM FABP5 at 0-80 °C.

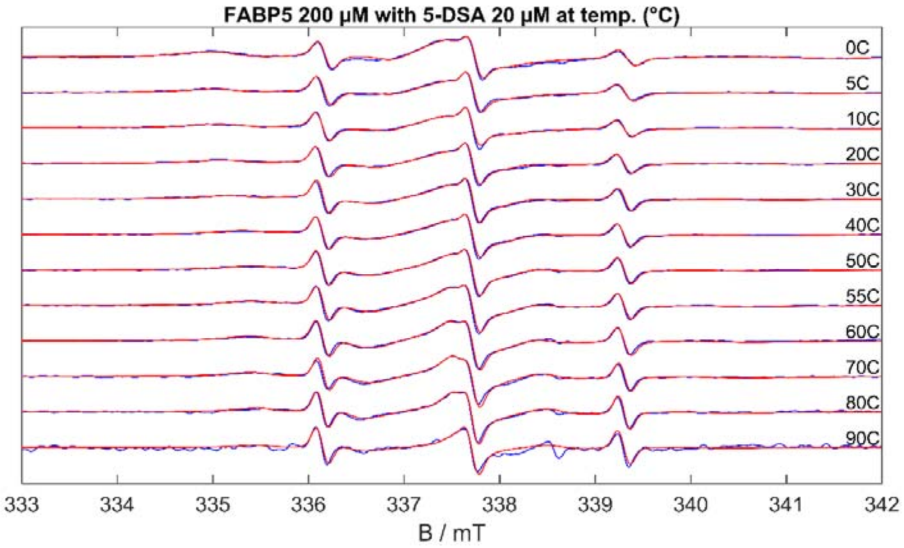

Figure S19. CW EPR spectra (blue) and simulations (red) of 20 µM 5-DSA with 200 µM FABP5 at 0-90 °C.

2.3 Temperature-dependent binding curves

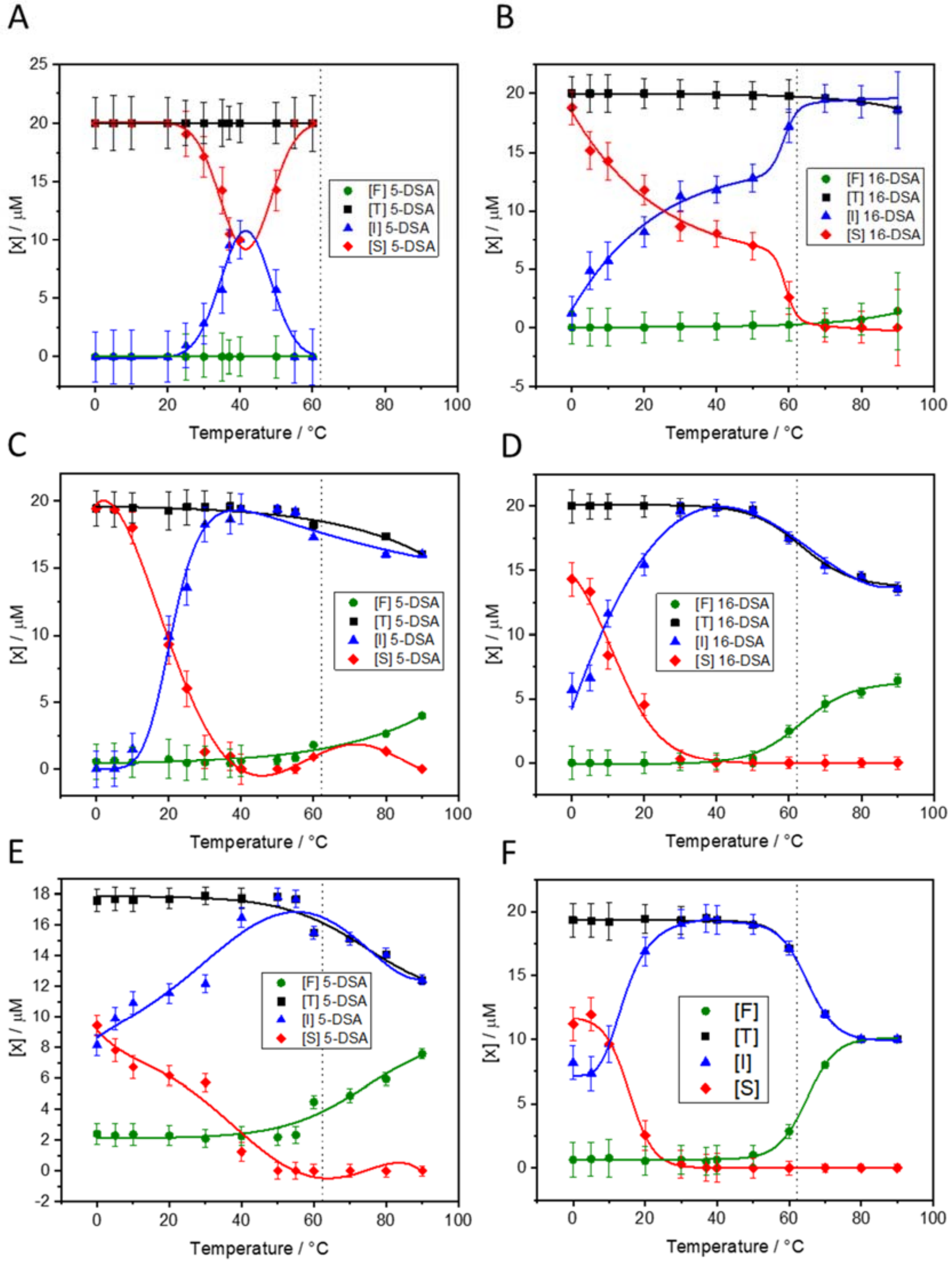

Figure S20. Temperature binding curves of 5-DSA with 200 (A), 100 (C), 50 (E) µM FABP3 and 16-DSA with 200 (B), 100 (D), 50 (F) µM FABP3. The guiding curves for 200 µM 16-DSA, 100 µM 5-DSA and 50 µM 5-DSA were slightly changed compared to (Michler et al., 2024) for visual reasons.

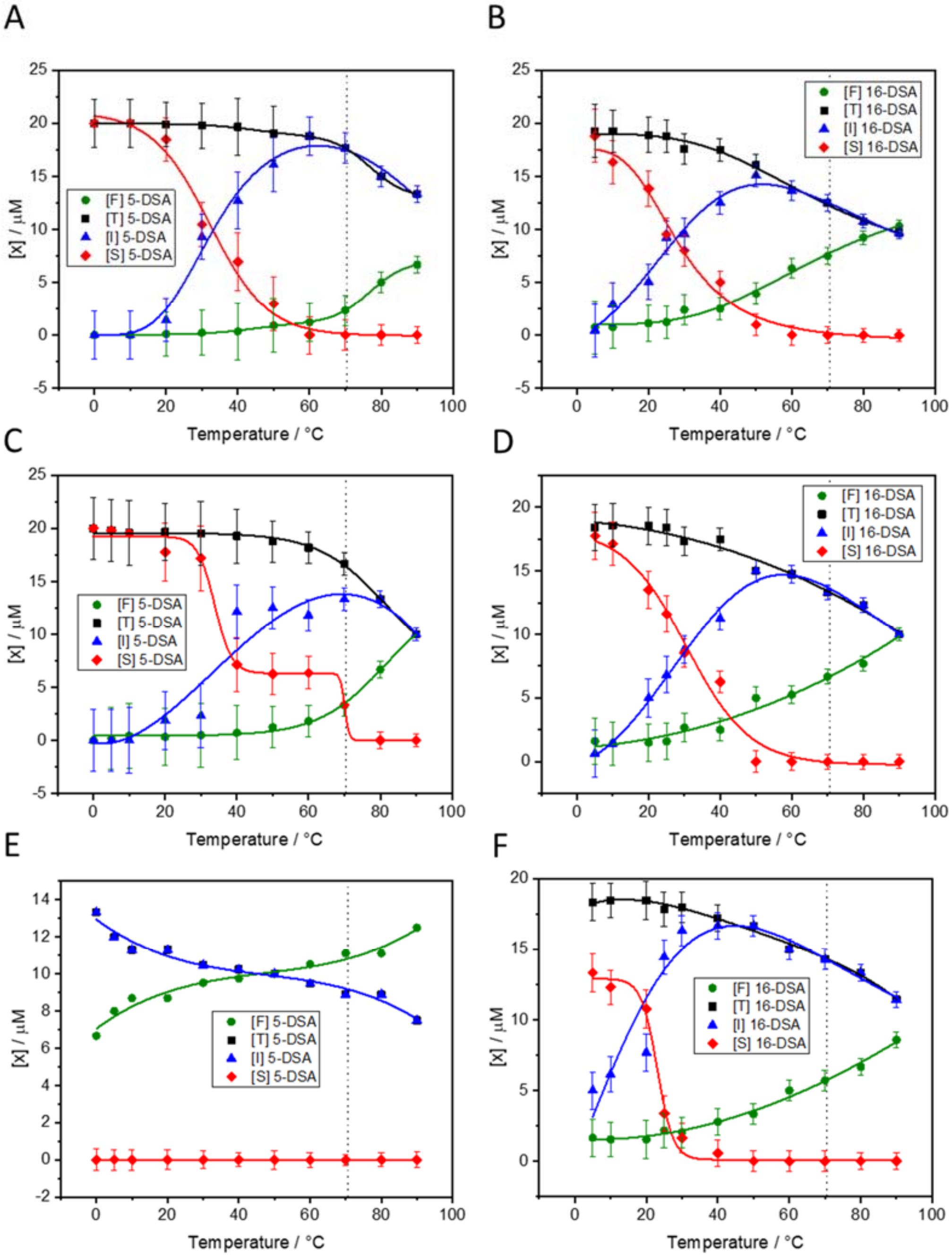

Figure S21. Temperature binding curves of 5-DSA with 200 (A), 100 (C), 50 (E) µM FABP4 and 16-DSA with 200 (B), 100 (D), 50 (F) µM FABP4.

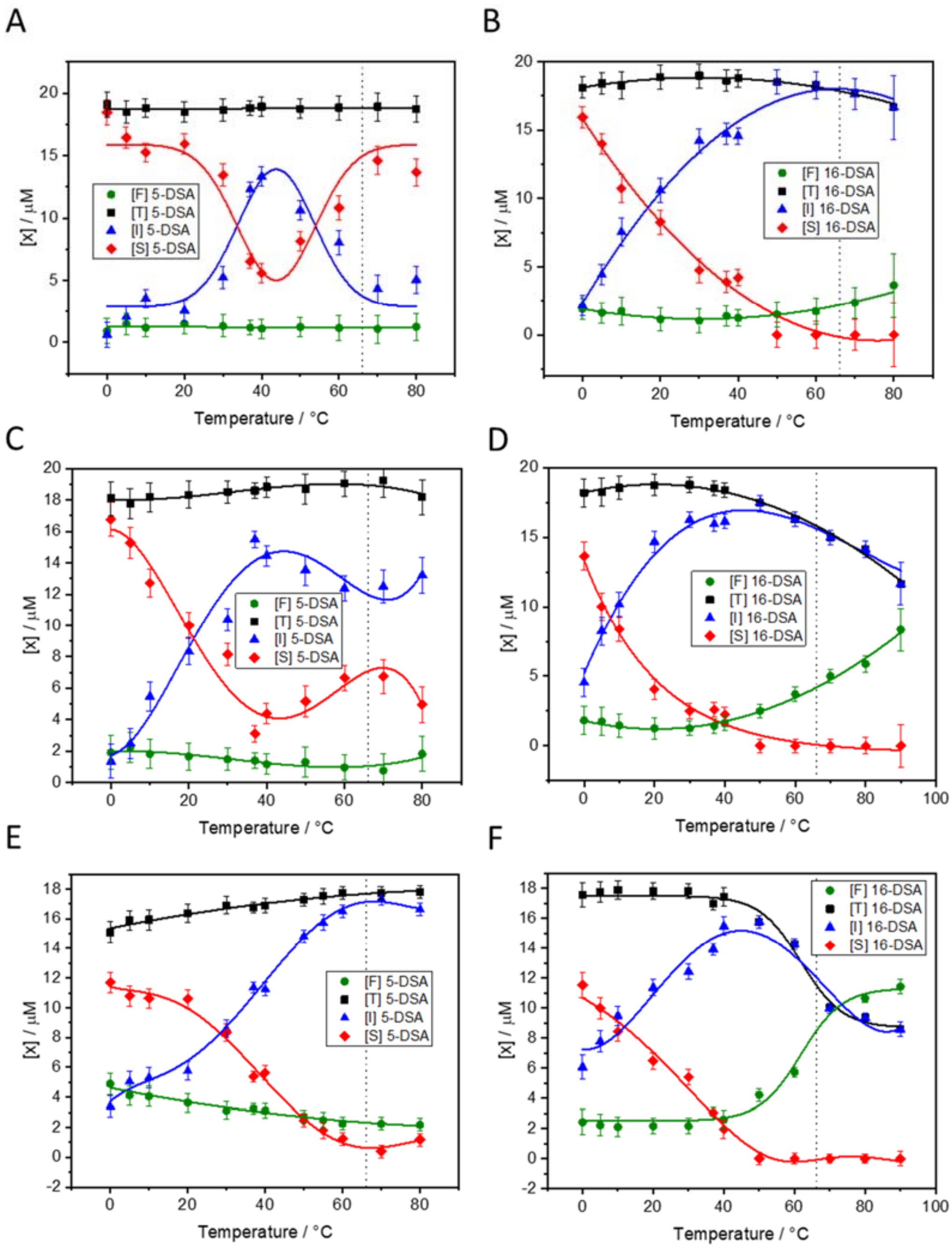

Figure S22. Temperature binding curves of 5-DSA with 200 (A), 100 (C), 50 (E) µM FABP5 and 16-DSA with 200 (B), 100 (D), 50 (F) µM FABP5. The guiding curves for 50 µM 5-DSA were slightly changed compared to (Michler et al., 2024) for visual reasons.

2.4 Thermodynamic profiles

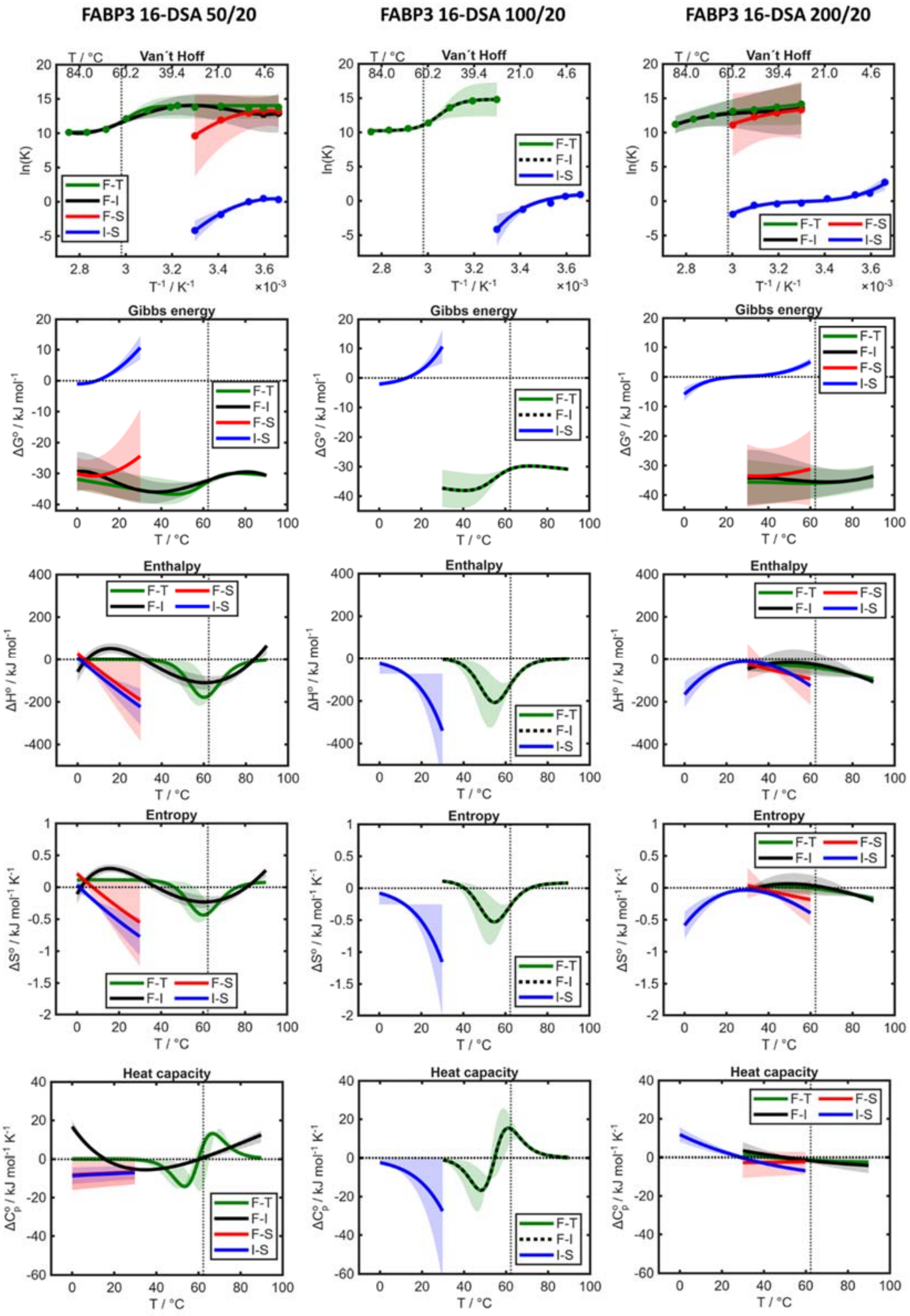

Figure S23. FABP3 with 16-DSA in the concentration regimes 50/20 µM, 100/20 µM and 200/20 µM. No curve is available for F-S in the regime 100/20 µM since always either [F] or [S] were equal to zero in the measured temperature range.

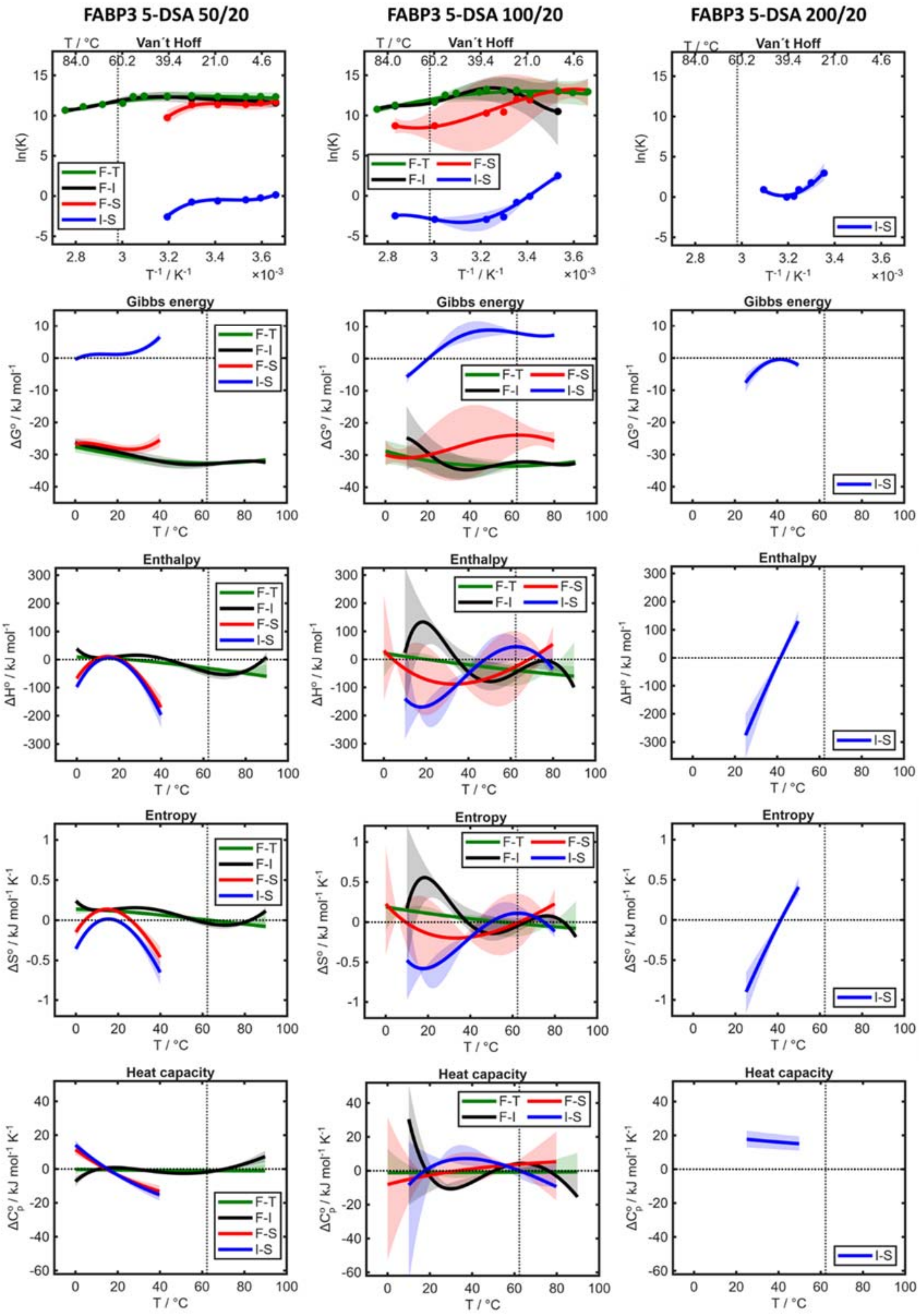

Figure S24. FABP3 with 5-DSA in the concentration regimes 50/20 µM, 100/20 µM and 200/20 µM. No curves are available for F-I, F-S and F-T of the system 200/20 µM since [F] was equal to zero in the entire temperature range.

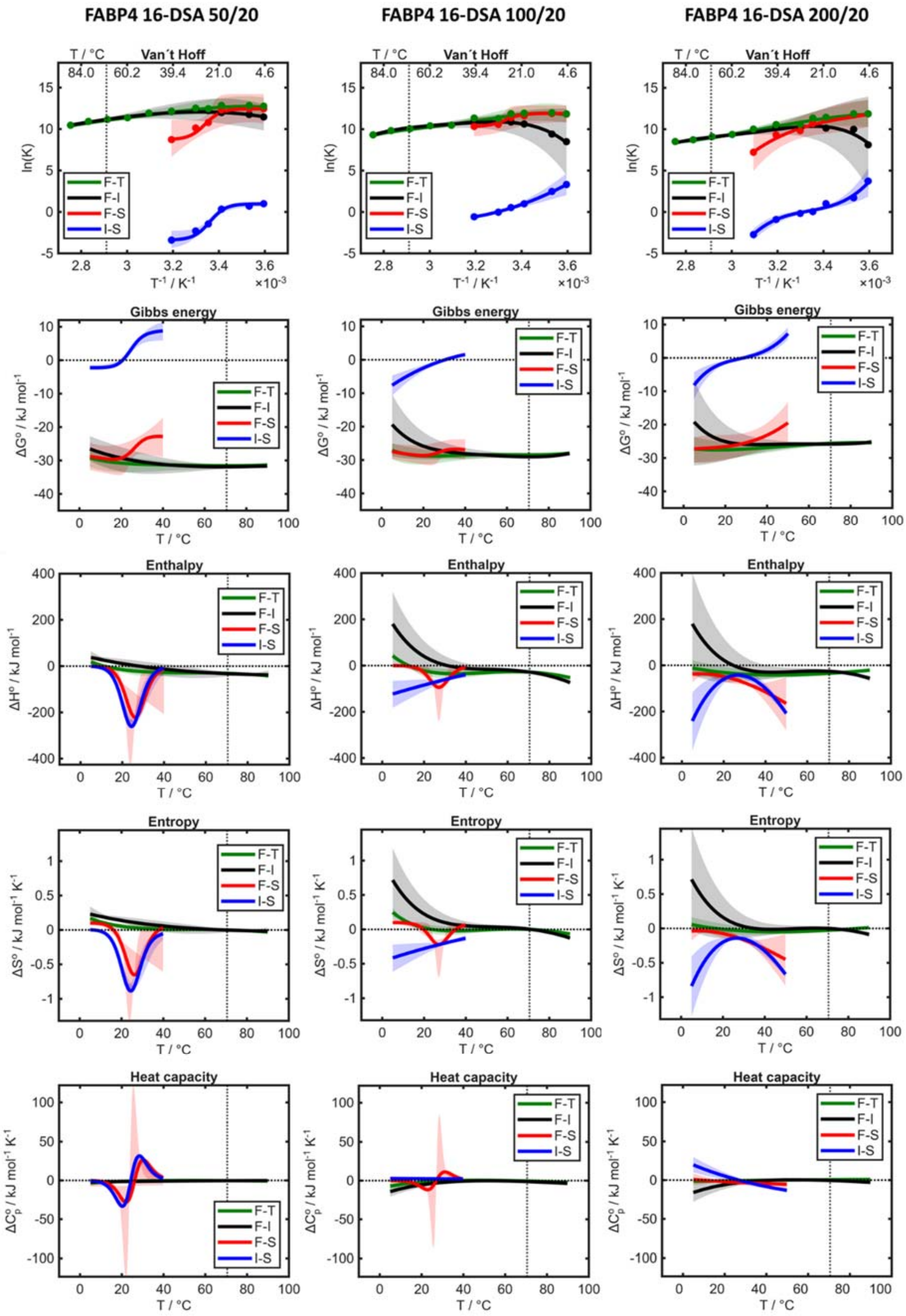

Figure S25. FABP4 with 16-DSA in the concentration regimes 50/20 μM, 100/20 μM and 200/20 μM.

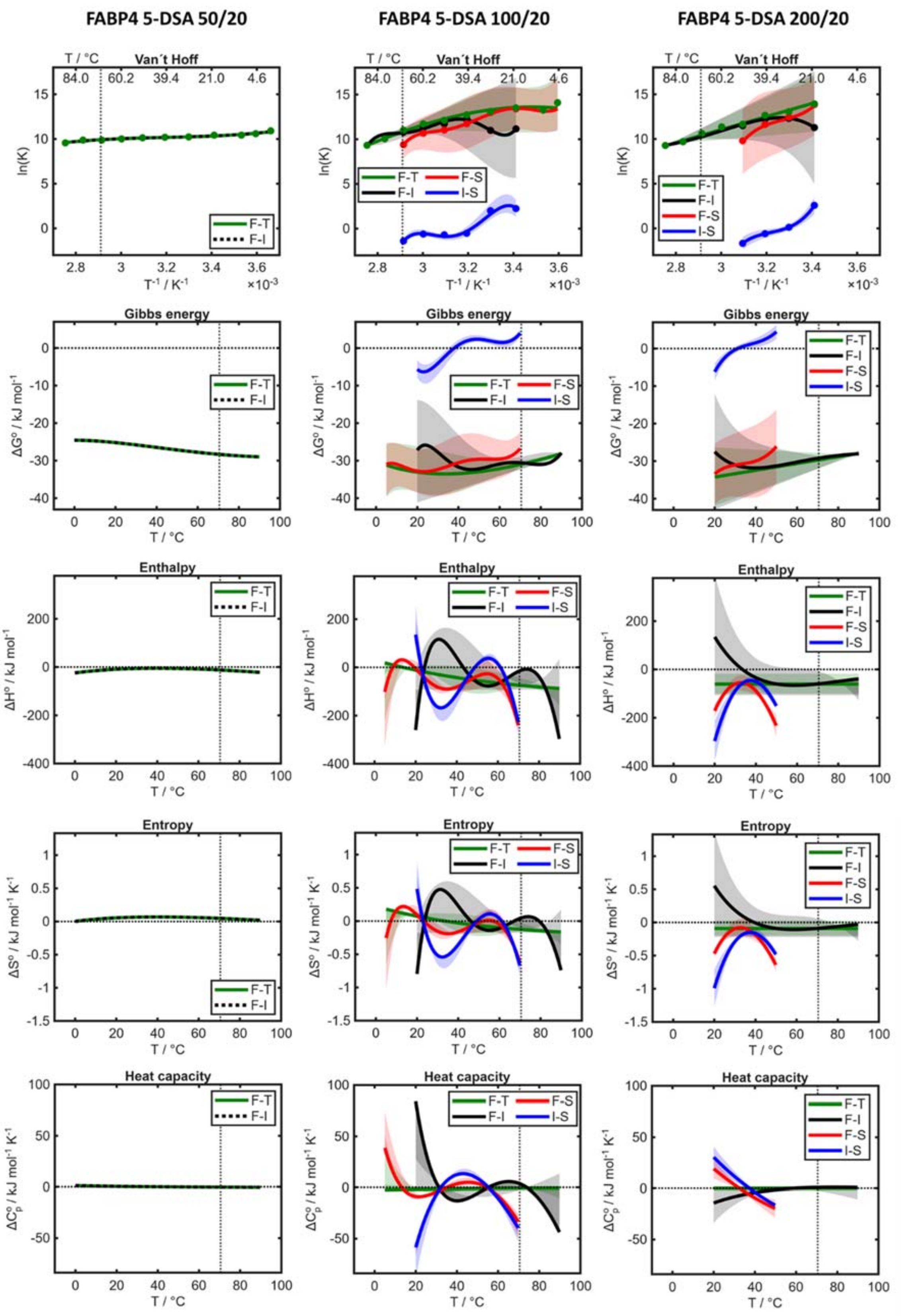

Figure S26. FABP4 with 5-DSA in the concentration regimes 50/20 μM, 100/20 μM and 200/20 μM. No curves are available for F-S and I-S in the system 50/20 μM since [S] is equal to 0 μM in the entire temperature range.

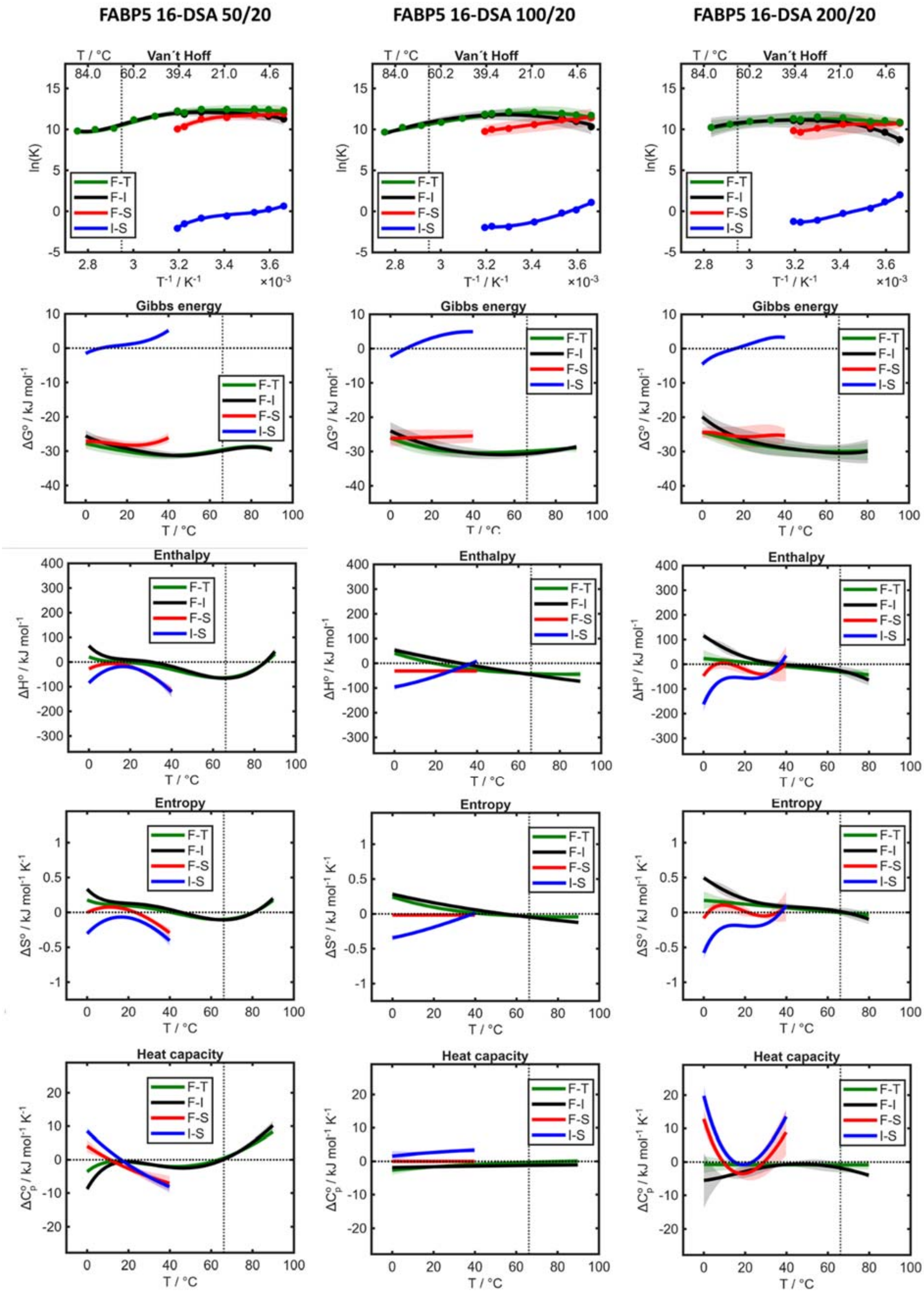

Figure S27. FABP5 with 16-DSA in the concentration regimes 50/20 µM, 100/20 µM and 200/20 µM.

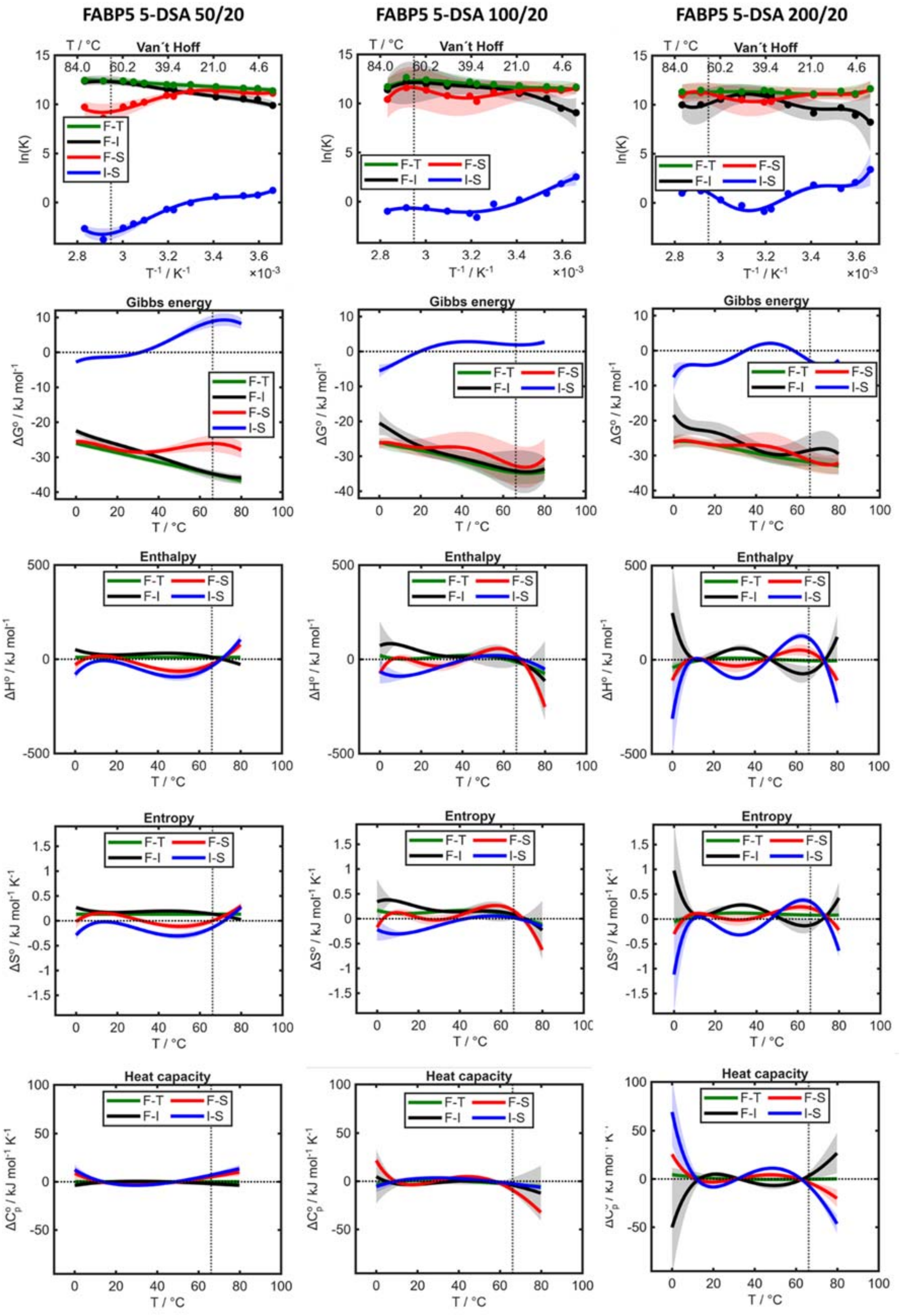

Figure S28. FABP5 with 5-DSA in the concentration regimes 50/20 μM, 100/20 μM and 200/20 μM.

2.5 Additional comparison of functional thermodynamics between FABPs

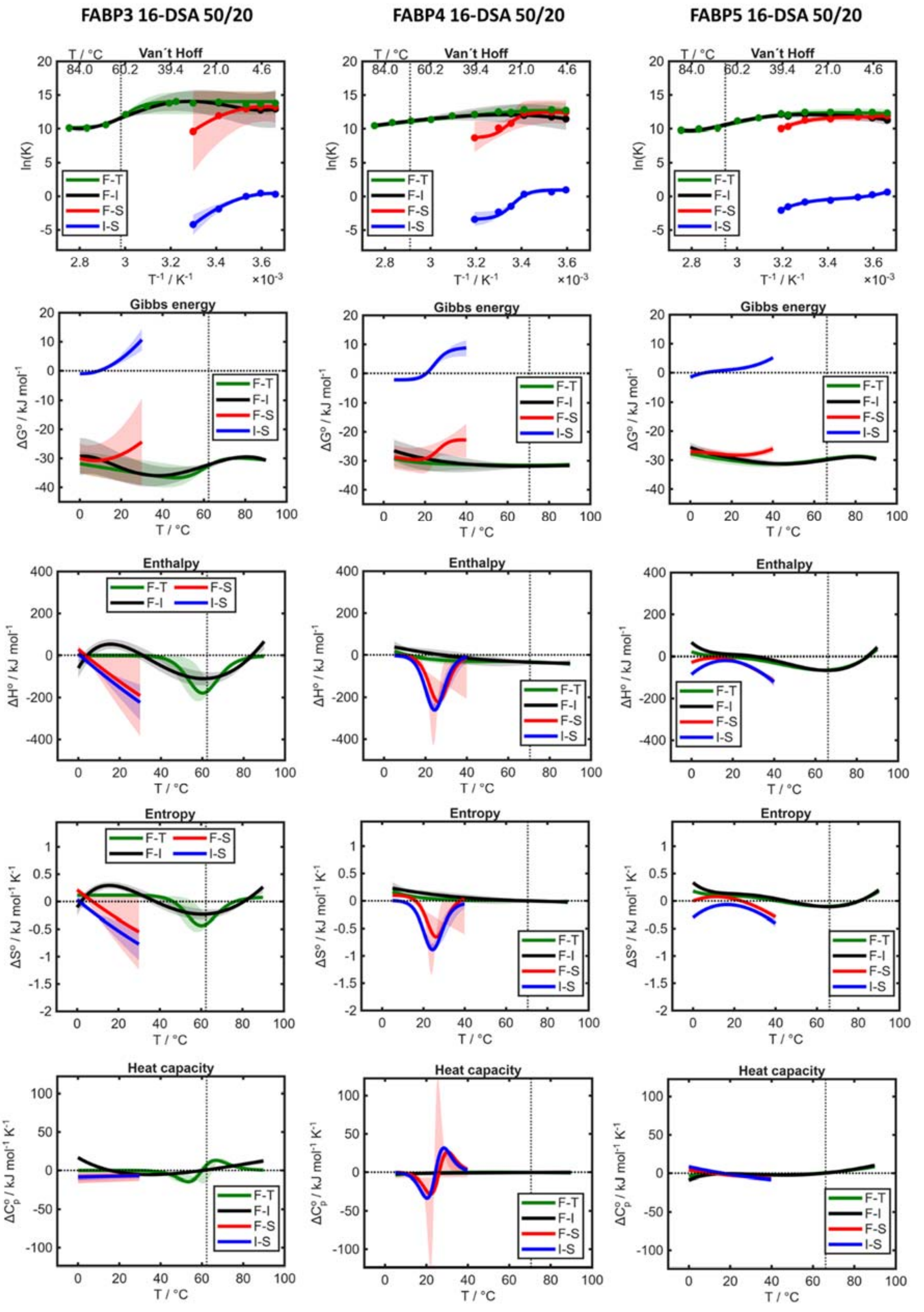

Figure S 29. FABP3/4/5 50 μM with 16-DSA 20 μM.

2.6 Sequence similarities of FABP3, FABP4 and FABP5

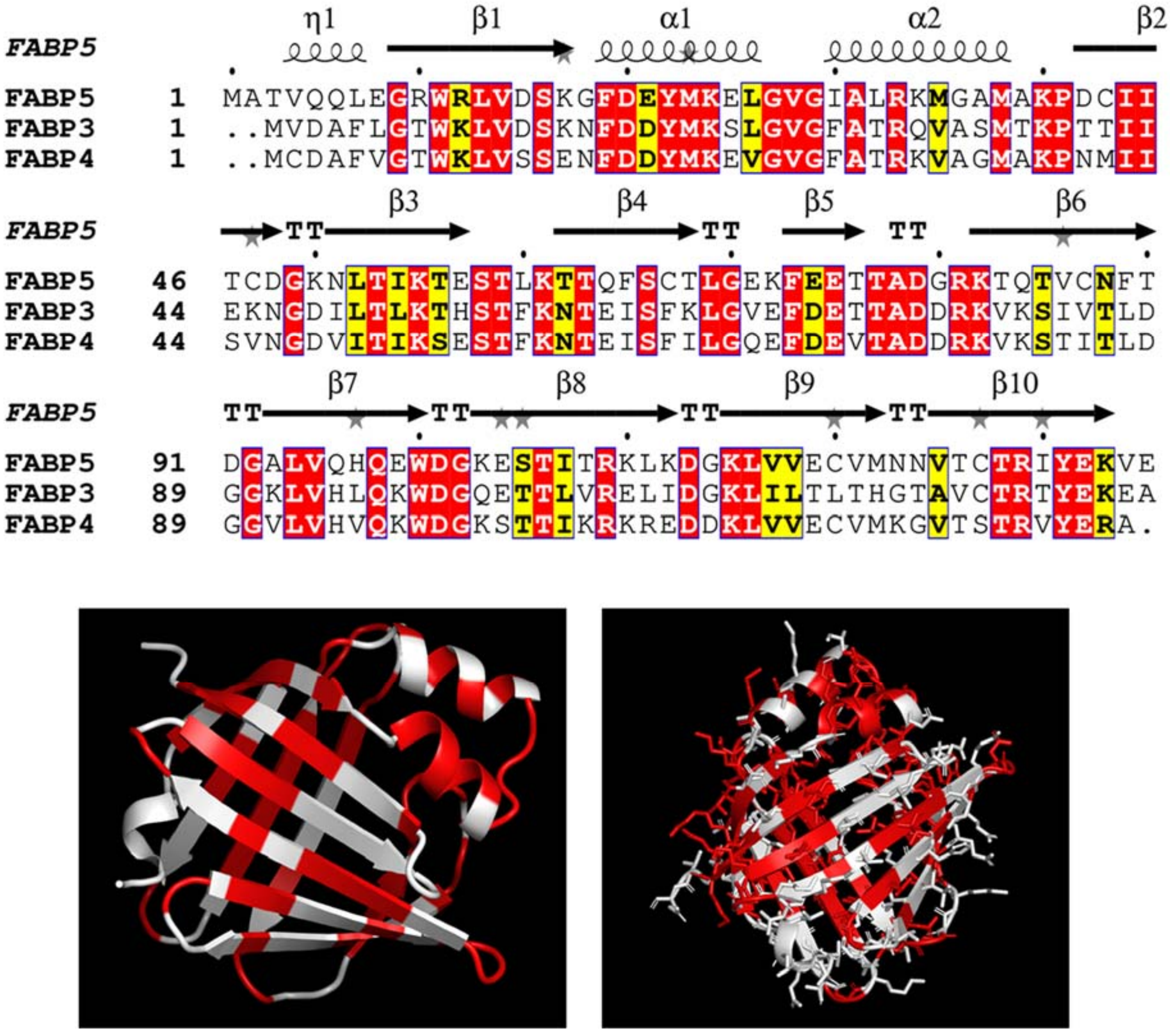

Figure S30. Amino acid sequence alignment and sequence similarities of FABP5, FABP3 and FABP4. Top: Alignment of all three FABPs, colored by similarity with: white font in red fields = identical amino acids, black font in yellow fields = similar properties of amino acids, black font in white fields = no similarity. Bottom: Crystal structure of FABP5 (Armstrong et al., 2014) with similar (red) and non-similar (white) amino acids from the alignment of all three FABPs. The crystal structure is shown from two different orientations, without and with amino acid residues as sticks.

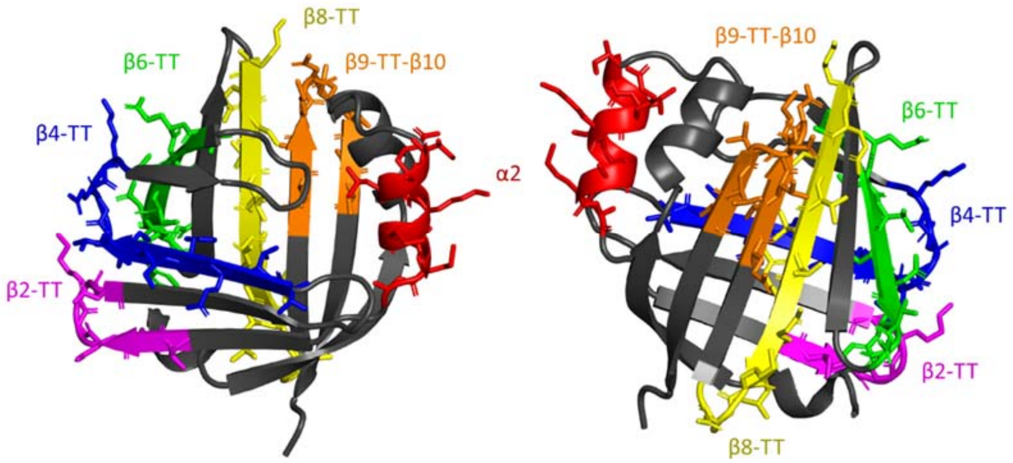

Figure S31. FABP5 crystal structure from two perspectives with colored domains of highest diversity among the three FABPs. (Armstrong et al., 2014) α2 (red) is most hydrophobic for FABP5 (red), β2-TT (magenta) is most hydrophobic for FABP4, β4-TT (blue) and β6-TT (green) are most hydrophilic for FABP5, β8-TT (yellow) and β9-TT-β10 (orange) are in the medium range for FABP5.

2.7 Linear and non-linear van't Hoff fitting

Classic linear van't Hoff fitting after plotting ln$K$ against $1/T$ assumes that $\Delta C_P°$ is equal to 0 and $\Delta H°$ and $\Delta S°$ are no functions of the temperature and therefore temperature-independent (constant).

$$\ln K = -\frac{\Delta G°}{RT} = -\frac{\Delta H° - T\Delta S°}{RT} = -\frac{\Delta H°}{RT} + \frac{\Delta S°}{R} \quad (20)$$

Most of our systems show a non-linear van't Hoff behavior instead, with strong deviations from the classic linearity in some cases. This circumstance opens the possibility to use the non-linear van't Hoff equation instead, assuming temperature-dependent $\Delta H°$ and $\Delta S°$ and constant $\Delta C_P°$. This equation has been already used in the literature to describe FABP binding thermodynamics (Richieri et al., 1996b).

$$\ln K = -\frac{\Delta H_0}{RT} + \frac{\Delta S_0}{R} + \frac{\Delta C_P°}{R} \cdot \left(1 - \frac{T_0}{T} + \frac{\Delta C_P°}{R} + \ln\frac{T}{T_0}\right) \quad (21)$$

T_0 is a reference value that could be chosen arbitrarily, best in the center of the dataset, $\Delta H°$ and the other standard values belong to this reference temperature, they should be set as free parameters in the fitting process. However, this strategy works only for constant $\Delta C_P°$ which we would not necessarily assume for FABP-FA ligand binding observed from the ligand's perspective. One possibility to take this aspect into account is to use a parametrized model for the heat capacity changes, such as a linear dependency:

$$\Delta C_P°(T) \approx a + b \cdot T \quad (22)$$

With: a - temperature independent part (J mol^{-1} K^{-1}); b - temperature coefficient (J mol^{-1} K^{-2})

However, van't Hoff equations with temperature-dependent ΔC_P contain many free parameters making the entire fitting process unstable. Hence, we decided to only apply the linear and the non-linear fitting with constant $\Delta C_P°$ as alternative, classic approach to the mathematical model fitting and plotted both with R^2 fit errors. Non-linear fitting was only performed successfully if the dataset included at least 5 datapoints.

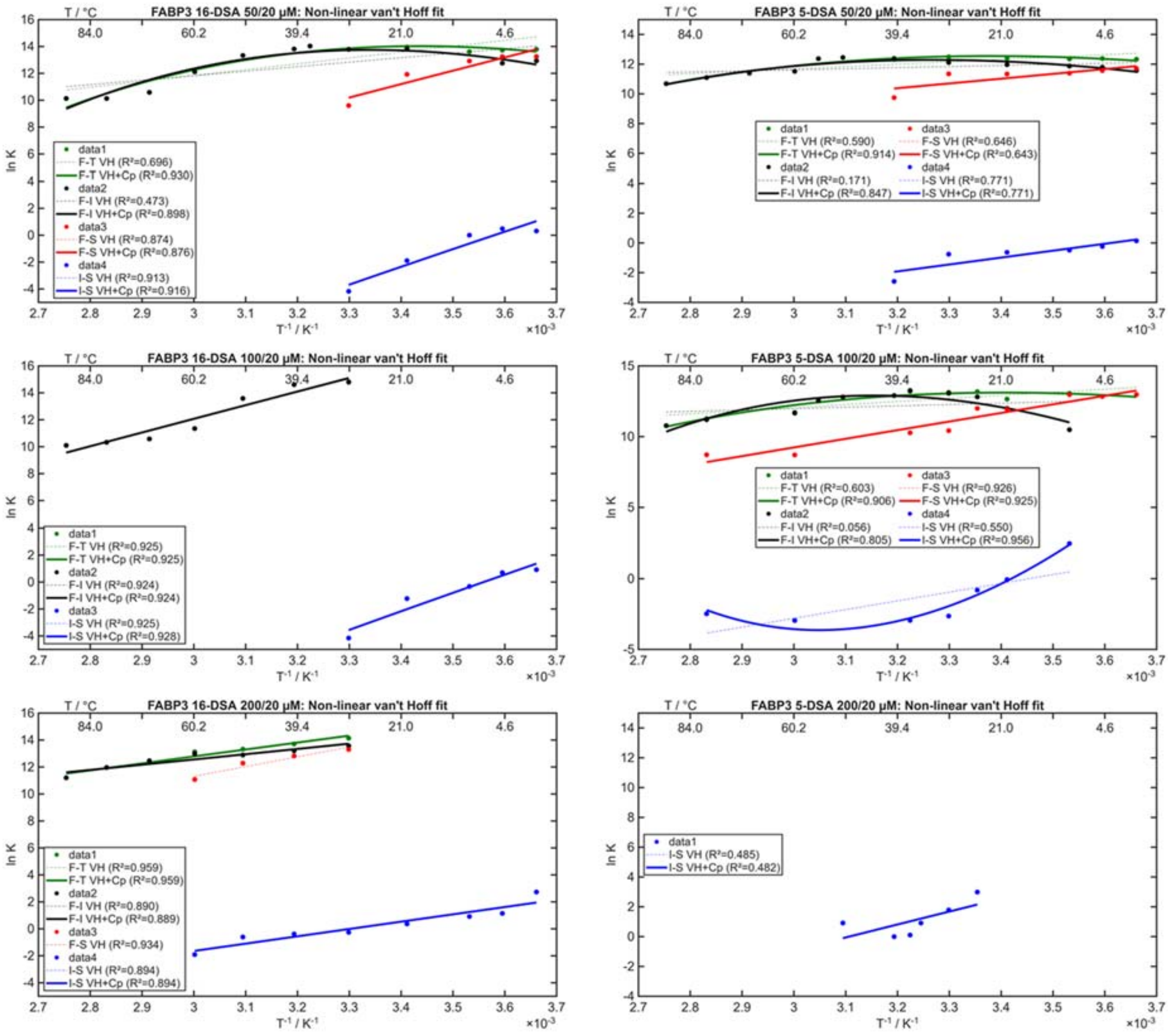

Figure S 32. Non-linear van't Hoff fitting of the data from FABP3 with 5/16-DSA. Non-linear fitting curves are drawn as solid lines, classic (linear) ones as dashed lines.

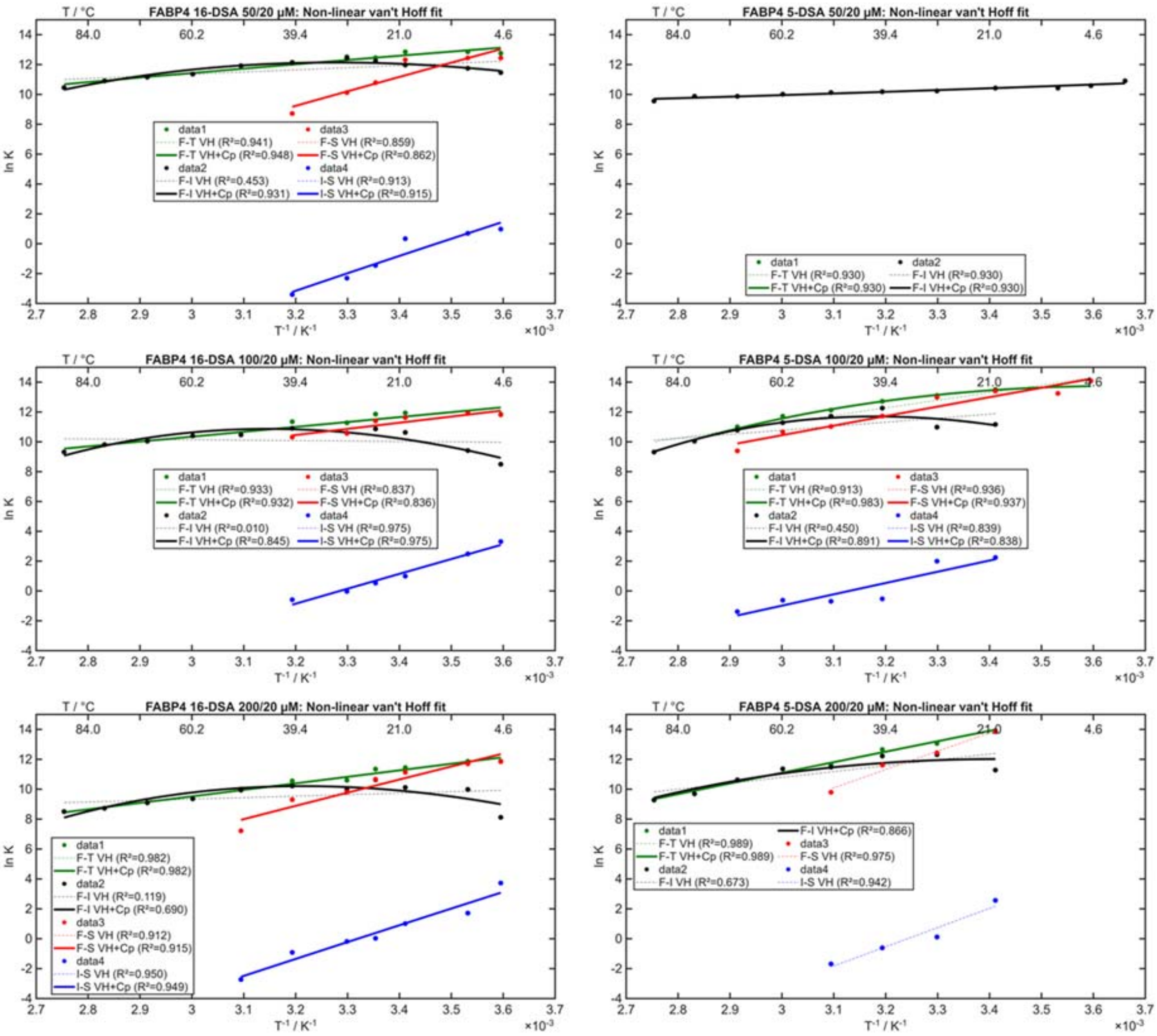

Figure S 33. Non-linear van't Hoff fitting of the data from FABP4 with 5/16-DSA. Non-linear fitting curves are drawn as solid lines, classic (linear) ones as dashed lines.

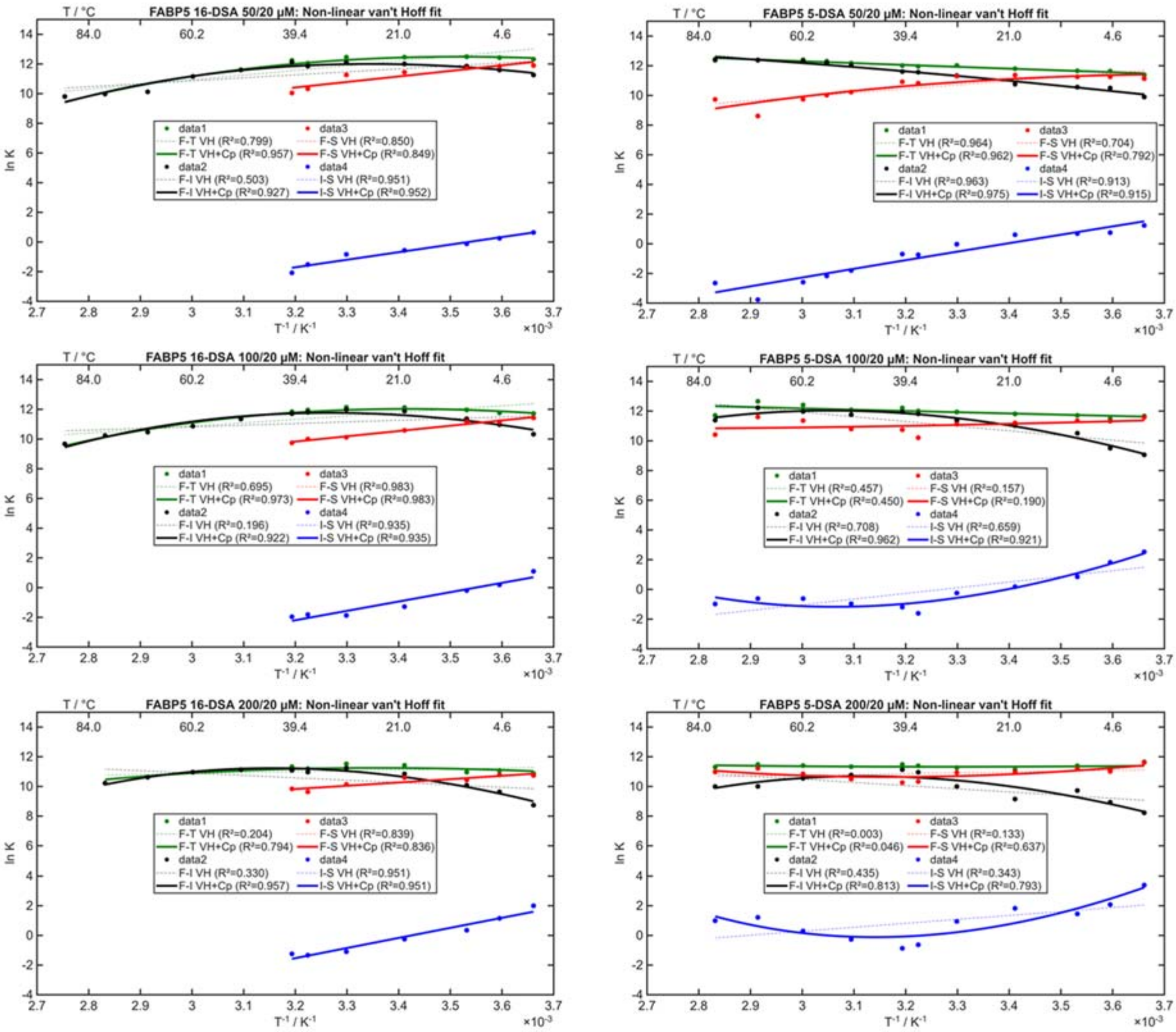

Figure S 34. Non-linear van't Hoff fitting of the data from FABP5 with 5/16-DSA. Non-linear fitting curves are drawn as solid lines, classic (linear) ones as dashed lines.

Comment: Extracted enthalpies, entropies and heat capacities from the linear and non-linear van't Hoff fitting functions are not given due the focus on the mathematical modelling strategy in this study and the large amount of data, but can be provided on request, while results for selected systems can be found in **Table S 16**.

2.8 ATR-IR spectroscopy on FABPs to determine their denaturation temperature

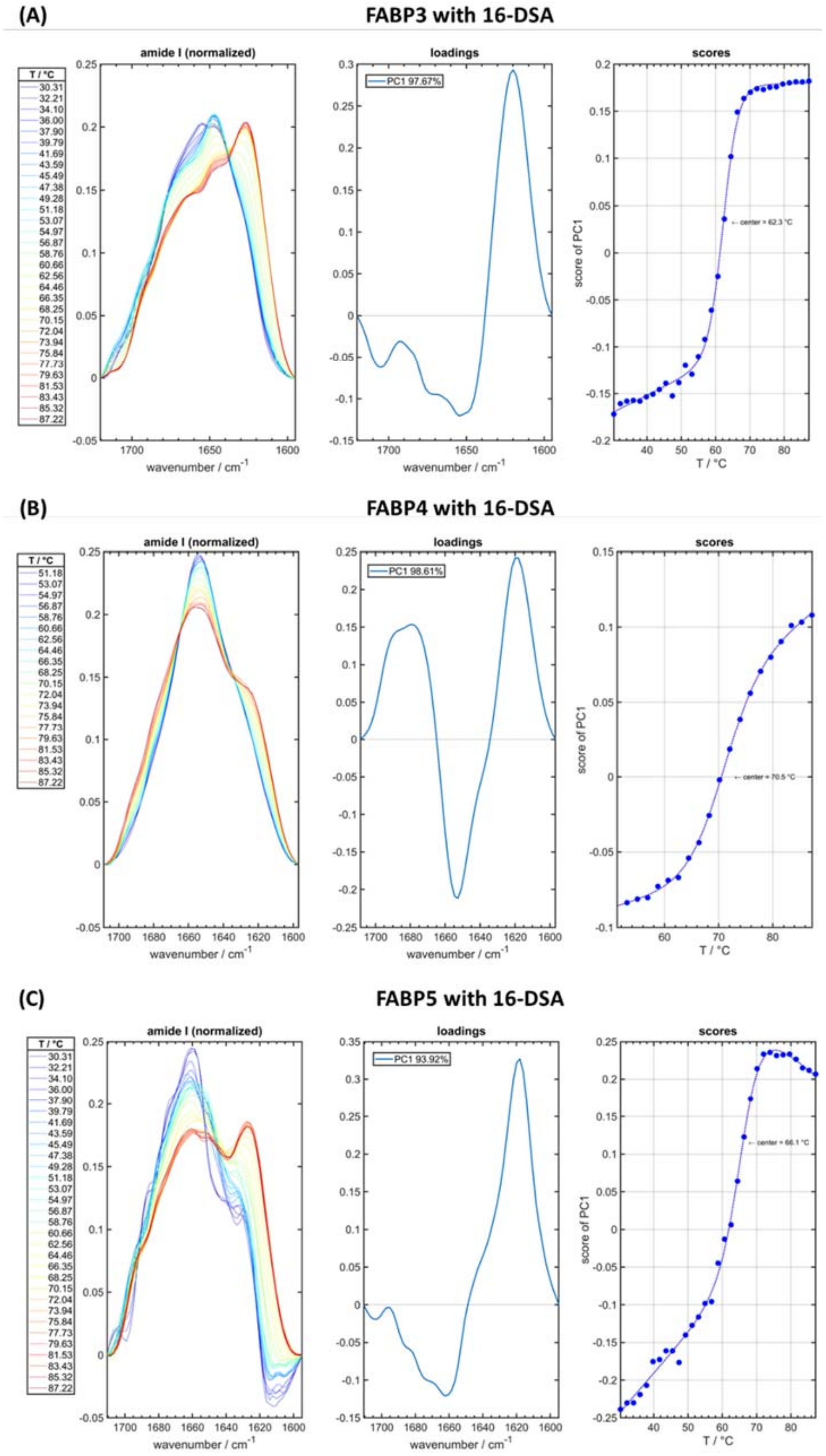

Figure S 35. Results of the principal component analysis applied on the amide I band of ATR-IR spectra from FABP3/4/5 with 16-DSA. (A) FABP3. (B) FABP4. (C) FABP5.

2.9 Application of the classic non-linear van't Hoff fitting on an example system

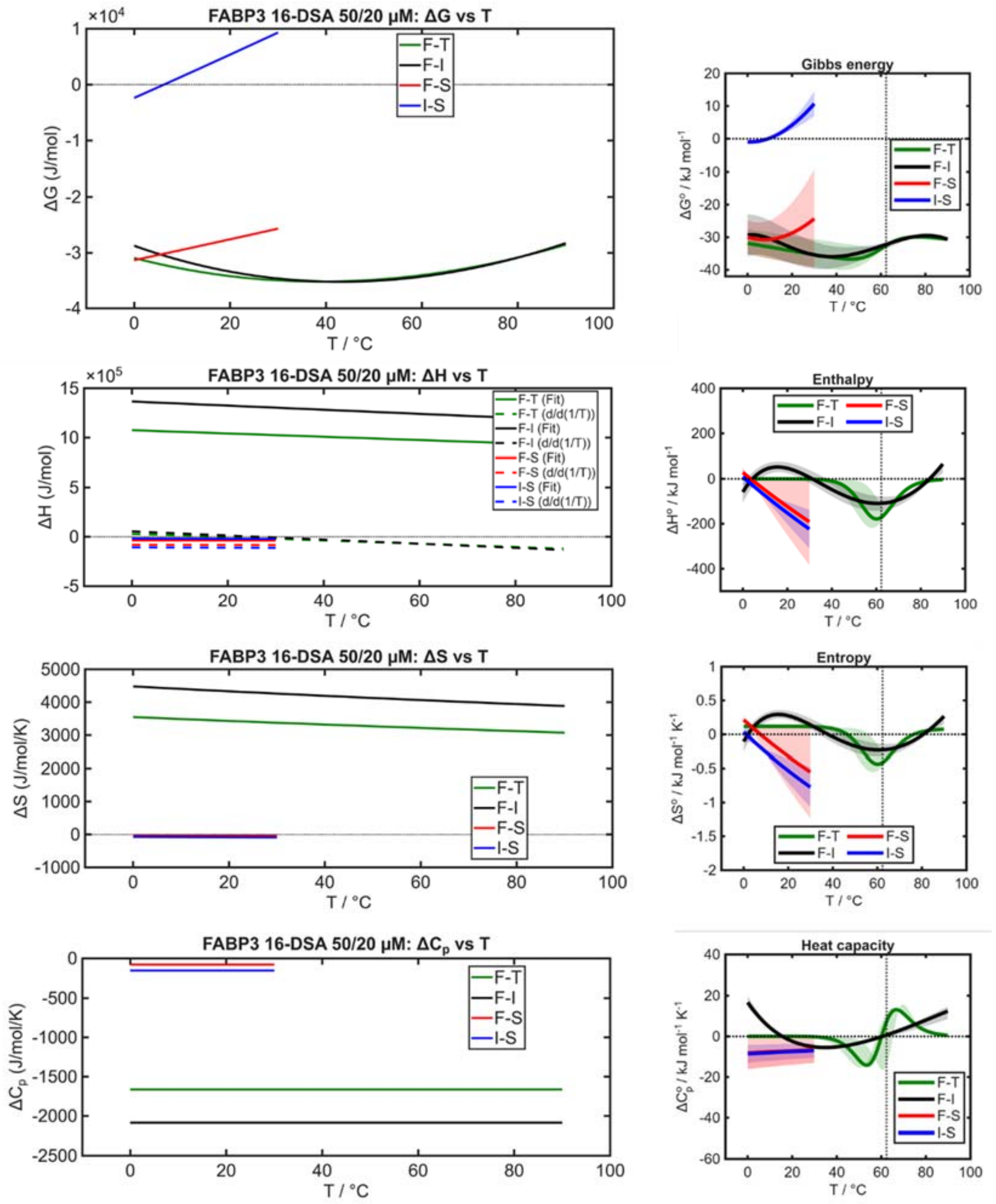

Figure S36. Example profile for FABP3/16-DSA 50/20 µM generated from the non-linearly fitted van't Hoff curves. The profile generated by our model fits is shown on the right side for comparison. Dashed fits in the enthalpy profile were calculated via differentiation.

Comment: Starting from the non-linearly fitted van't Hoff curves with constant C_p, the thermodynamic parameters were here calculated via the following equations:

$dH = dH_0 + C_p \cdot (T - T_0)$;

$dS = dH_0 + C_p \cdot \log(T/T_0)$;

$dG = -R \cdot T \cdot \ln K$ (fit)

C_p = constant

The resulting curves show partially strong differences to the curves calculated with our model functions, especially in case of enthalpies and entropies of F-I and F-T where no zero transition is found in the classic model. The calculation of dH via differentiation instead leads to similar curves as in our modelling strategy with zero transitions. The Gibbs energies of all transitions and the enthalpies/entropies of F-S and I-S show higher similarity to the results of the other strategy. The strong difference between dH calculation via the classic equation with constant C_p (see above) and the differentiation argues for a non-suitable or temperature-dependent C_p and argues rather for the application of our modelling approach instead of the non-linear van't Hoff fitting.

3. Supplementary Tables

3.1 Model fit functions for van't Hoff plots

Table S1. Van't Hoff curve fitting functions for all systems and transitions. PolyX stands for polynomial fitting function of the Xth order.

System	Transition	Fit function type	Fit function equation
**FABP3/ 5-DSA 200/20**	F-I	-	-
	F-S	-	-
	I-S	Poly2	$y = 956.7921 + (-602029.7474) \cdot x + 9.47186 \cdot 10^{7} \cdot x^{2}$
	F-T	-	-
**FABP3/ 5-DSA 100/20**	F-I	Poly5	$y = -144985.1813 + 2.3425 \cdot 10^{8} \cdot x + (-1.50998 \cdot 10^{11}) \cdot x^{2} + 4.85403 \cdot 10^{13} \cdot x^{3} + (-7.78091 \cdot 10^{15}) \cdot x^{4} + 4.97523 \cdot 10^{17} \cdot x^{5}$
	F-S	Poly3	$y = 941.72263 + (-872711.4733) \cdot x + 2.69533 \cdot 10^{8} \cdot x^{2} + (-2.74399 \cdot 10^{10}) \cdot x^{3}$
	I-S	Poly4	$y = (-13694.58444) + 1.73197 \cdot 10^{7} \cdot x + (-8.17904 \cdot 10^{9}) \cdot x^{2} + 1.70843 \cdot 10^{12} \cdot x^{3} + (-1.33134 \cdot 10^{14}) \cdot x^{4}$
	F-T	Poly2	$y = (-51.3818) + 37772.23358 \cdot x + (-5539779.7922) \cdot x^{2}$
**FABP3/ 5-DSA 50/20**	F-I	Poly5	$y = 31253.73191 + (-4.87575 \cdot 10^{7}) \cdot x + 3.03237 \cdot 10^{10} \cdot x^{2} + (-9.39656 \cdot 10^{12}) \cdot x^{3} + 1.45111 \cdot 10^{15} \cdot x^{4} + (-8.93644 \cdot 10^{16}) \cdot x^{5}$
	F-S	Poly3	$y = (-3872.587) + 3356376.45512 \cdot x + (-9.66386 \cdot 10^{8}) \cdot x^{2} + 9.27092 \cdot 10^{10} \cdot x^{3}$
	I-S	Poly3	$y = (-4572.20644) + 3958598.59795 \cdot x + (-1.14237 \cdot 10^{9}) \cdot x^{2} + 1.0987 \cdot 10^{11} \cdot x^{3}$
	F-T	Poly3	$y = (-134.03202) + 119378.90593 \cdot x + (-3.21658 \cdot 10^{7}) \cdot x^{2} + 2.85849 \cdot 10^{9} \cdot x^{3}$
**FABP3/ 16-DSA 200/20**	F-I	Poly3	$y = (-921.10852) + 901213.09551 \cdot x + (-2.90379 \cdot 10^{8}) \cdot x^{2} + 3.12467 \cdot 10^{10} \cdot x^{3}$
	F-S	Poly3	$y = (-210.80674) + 134578.41624 \cdot x + (-2.02165 \cdot 10^{7}) \cdot x^{2}$
	I-S	Poly3	$y = (-1810.89494) + 1640587.90071 \cdot x + (-4.95812 \cdot 10^{8}) \cdot x^{2} + 4.99841 \cdot 10^{10} \cdot x^{3}$
	F-T	Poly3	$y = (-508.62404) + 490662.25357 \cdot x + (-1.54741 \cdot 10^{8}) \cdot x^{2} + 1.63817 \cdot 10^{10} \cdot x^{3}$
**FABP3/ 16-DSA 100/20**	F-I	Dose Response	$y = 10.2297 + (14.8301 - 10.2297) / (1 + 10^{((0.00305 - x) \cdot 9409.27019)})$
	F-S	-	-
	I-S	Exp. Decay	$y = 1.59137 + (-5.5803) \cdot \exp(-(x - 0.0033) / 1.76757 \cdot 10^{(-4)})$
	F-T	Dose Response	$y = 10.2297 + (14.8301 - 10.2297) / (1 + 10^{((0.00305 - x) \cdot 9409.27019)})$
**FABP3/ 16-DSA 50/20**	F-I	Poly4	$y = 10097.18455 + (-1.27017) \cdot 10^{7} \cdot x + 5.96391 \cdot 10^{9} \cdot x^{2} + (-1.23762 \cdot 10^{12}) \cdot x^{3} + 9.58073 \cdot 10^{13} \cdot x^{4}$
	F-S	Poly2	$y = (-463.94849) + 264136.52185 \cdot x + (-3.65487 \cdot 10^{7}) \cdot x^{2}$
	I-S	Poly2	$y = (-513.50199) + 281849.0442 \cdot x + (-3.86422 \cdot 10^{7}) \cdot x^{2}$
	F-T	Boltzmann	$y = 14.02761 + (10.12663 - 14.02761) / (1 + \exp((x - 0.003) / 4.53655 \cdot 10^{(-5)}))$
**FABP4/ 5-DSA 200/20**	F-I	Poly4	$y = (-2471.85516) + 3542472.25973 \cdot x + (-1.89413 \cdot 10^{9}) \cdot x^{2} + 4.48864 \cdot 10^{11} \cdot x^{3} + (-3.97139 \cdot 10^{13}) \cdot x^{4}$
	F-S	Poly3	$y = (-8200.94066) + 7523041.28998 \cdot x + (-2.29901 \cdot 10^{9}) \cdot x^{2} + 2.34395 \cdot 10^{11} \cdot x^{3}$

	I-S	Poly3	y = (-9197.85136) + 8560219.35777 · x + (-2.65742 · 10^9) · x^2 + 2.75162 · 10^11 · x^3
	F-T	Line	y = (-10.84029) + 7294.47293 · x
**FABP4/ 5-DSA 100/20**	F-I	Poly5	y = (-479347.75376) + 7.85207 · 10^8 · x + (-5.13781 · 10^11) · x^2 + 1.67856 · 10^14 · x^3 + (-2.73806 · 10^16) · x^4 + 1.78393 · 10^18 · x^5
	F-S	Poly5	y = (-334423.57423) + 5.15755 · 10^8 · x + (-3.17696 · 10^11) · x^2 + 9.77031 · 10^13 · x^3 + (-1.50009 · 10^16) · x^4 + 9.19867 · 10^17 · x^5
	I-S	Poly4	y = -99741.31992 + 1.26802 · 10^8 · x + (-6.03778 · 10^10) · x^2 + 1.27613 · 10^13 · x^3 + (-1.0101 · 10^15) · x^4
	F-T	Poly3	y = (-5.66936) + (-17523.25599) · x + 1.48259 · 10^7 · x^2 + (-2.35589 · 10^9) · x^3
**FABP4/ 5-DSA 50/20**	F-I	Poly3	y = (-106.6005) + 108447.34157 · x + (-3.3759 · 10^7) · x^2 + 3.52279 · 10^9 · x^3
	F-S	-	-
	I-S	-	-
	F-T	Poly3	y = (-106.6005) + 108447.34157 · x + (-3.3759 · 10^7) * x^2 + 3.52279 · 10^9 · x^3
**FABP4/ 16-DSA 200/20**	F-I	Poly4	y = (-3930.22795) + 5157021.06855 · x + (-2.53204 · 10^9) · x^2 + 5.52321 · 10^11 · x^3 + (-4.51332 · 10^13) · x^4
	F-S	Poly3	y = (-931.19537) + 806631.94418 · x + (-2.32018 · 10^8) · x^2 + 2.24331 · 10^10 · x^3
	I-S	Poly3	y = (-4345.70234) + 3900996.89715 · x + (-1.16877 · 10^9) · x^2 + 1.16877 · 10^11 · x^3
	F-T	Poly3	y = 172.28231 + (-165383.80808) · x + 5.43631 +10^7 · x^2 + (-5.77937 · 10^9) · x^3
**FABP4/ 16-DSA 100/20**	F-I	Poly4	y = (-3746.42543) + 4858617.98693 · x + (-2.35963 · 10^9) · x^2 + 5.09855 · 10^11 · x^3 + (-4.13391 · 10^13) · x^4
	F-S	Dose Response	y = 10.319 + (11.9007 - 10.319) / (1 + 10^((0.00333 - x) · 12443.23893))
	I-S	Poly2	y = 113.40187 + (-76045.58171) · x + 1.26343 · 10^7 · x^2
	F-T	Poly4	y = (-2355.56236) + 3049736.44759 · x + (-1.47476 · 10^9) · x^2 + 3.16726 · 10^11 · x^3 + (-2.54585 · 10^13) · x^4
**FABP4/ 16-DSA 50/20**	F-I	Poly3	y = 57.11939 + (-63319.35396) · x + 2.58868 · 10^7 · x^2 + (-3.28378 · 10^9) · x^3
	F-S	Dose Response	y = 8.71633 + (12.44375 – 8.71633) / (1 + 10^((0.00334 - x) · 12443.23893))
	I-S	Dose Response	y = (-3.40119) + (0.98083 - (-3.40119)) / (1 + 10^((0.00336 - x) · 12443.23893))
	F-T	Poly4	y = (-879.89975) + 1144474.31085 · x + (-5.54552 · 10^8) · x^2 + 1.1986 · 10^11 · x^3 + (-9.72106 · 10^12) · x^4
**FABP5/ 5-DSA 200/20**	F-I	Poly5	y = 175902.0693 + (-2.73527 · 10^8) · x + 1.6975 · 10^11 · x^2 + (-5.25533 · 10^13) · x^3 + 8.11698 · 10^15 · x^4 + (-5.00393 · 10^17) · x^5
	F-S	Poly5	y = (-112131.34083) + 1.73413 · 10^8 · x + (-1.07016 · 10^11) · x^2 + 3.29458 · 10^13 · x^3 + (-5.0601 · 10^15) · x^4 + 3.10201 · 10^17 · x^5
	I-S	Poly5	y = (-280874.42289) + 4.35373 · 10^8 · x + (-2.69301 · 10^11) · x^2 + 8.30948 · 10^13 · x^3 + (-1.27906 · 10^16) · x^4 + 7.85793 · 10^17 · x^5
	F-T	Poly4	y = 1074.99874 + (-1394789.2829) · x + 6.82827 · 10^8 · x^2 + (-1.47909) · 10^11 · x^3 + 1.19602 · 10^13 · x^4
**FABP5/ 5-DSA 100/20**	F-I	Poly5	y = (-50567.13587) + 7.74362 · 10^7 · x + (-4.73283 · 10^10) · x^2 + 1.44338 · 10^13 · x^3 + (-2.19626 · 10^15) · x^4 + 1.3337 · 10^17 · x^5
	F-S	Poly5	y = (-139291.7737) + 2.13284 · 10^8 · x + (-1.30336 · 10^11) · x^2 + 3.97386 · 10^13 · x^3 + (-6.04535 · 10^15) · x^4 + 3.67118 · 10^17 · x^5
	I-S	Poly4	y = (-6091.78463) + 7529237.79741 · x + (-3.47316 · 10^9) · x^2 + 7.0831 · 10^11 · x^3 + (-5.38599 · 10^13) · x^4

	F-T	Poly4	y = (-3833.76996) + 4656660.29915 · x + (-2.10799 · 10^9) · x^2 + 4.22926 · 10^11 · x^3 + (-3.17405 · 10^13) · x^4
**FABP5/ 5-DSA 50/20**	F-I	Poly4	y = -2768.89158 + 3409352.30046 · x + (-1.56199 · 10^9) · x^2 + 3.17177 · 10^11 · x^3 + (-2.41089 · 10^13) · x^4
	F-S	Poly4	y = 8321.37805 + (-1.02124 · 10^7) · x + 4.68513 · 10^9 · x^2 + (-9.5133 · 10^11) · x^3 + 7.2162 · 10^13 · x^4
	I-S	Poly4	y = 11016.09597 + (-1.35311 · 10^7) · x + 6.20571 · 10^9 · x^2 + (-1.26013 · 10^12) · x^3 + 9.56386 · 10^13 · x^4
	F-T	Line	y = 16.06864 + (-1256.55916) · x
**FABP5/ 16-DSA 200/20**	F-I	Poly5	y = (-11431.47911) + 1.71318 · 10^7 · x + (-1.02265 · 10^10) · x^2 + 3.03974 · 10^12 · x^3 + (-4.49449 · 10^14) · x^4 + 2.64122 · 10^16 · x^5
	F-S	Poly4	y = 30529.14615 + (-3.57967 ·10^7) · x + 1.57254 · 10^10 · x^2 + (-3.06661 · 10^12) · x^3 + 2.24003 · 10^14 · x^4
	I-S	Poly4	y = 37238.45667 + (-4.35883 * 10^7) · x + 1.91227 · 10^10 · x^2 + (-3.72723 · 10^12) · x^3 + 2.7237 · 10^14 · x^4
	F-T	Poly3	y = (-84.31308) + 71783.86416 · x + (-1.71791 · 10^7) · x^2 + 1.27357 · 10^9 · x^3
**FABP5/ 16-DSA 100/20**	F-I	Poly2	y = (-78.34204) + 54978.46914 · x + (-8383668.00362) · x^2
	F-S	Line	y = (-2.16049) + 3741.78251 · x
	I-S	Poly3	y = 507.5733 + (-412708.49588) · x + 1.08927 · 10^8 · x^2 + (-9.2837 · 10^9) · x^3
	F-T	Poly3	y = 95.36143 + (-102376.62466) · x + 3.84983 · 10^7 · x^2 + (-4.58693 · 10^9) · x^3
**FABP5/ 16-DSA 50/20**	F-I	Poly5	y = 32831.47635 + (-5.07579 · 10^7) · x + 3.13013 · 10^10 · x^2 + (-9.62456 · 10^12) · x^3 + 1.47607 · 10^15 · x^4 + (-9.03568 · 10^16) · x^5
	F-S	Poly3	y = (-1857.54416) + 1593117.04214 · x + (-4.52794 · 10^8) · x^2 + 4.29167 · 10^10 · x^3
	I-S	Poly3	y = (-2542.81664) + 2204836.70492 · x + (-6.37985 · 10^8) · x^2 + 6.15973 · 10^10 · x^3
	F-T	Poly5	y = 22248.27267 + (-3.40057 · 10^7) · x + 2.07246 · 10^10 · x^2 + (-6.29504 · 10^12) · x^3 + 9.53403 · 10^14 · x^4 + (-5.76198 · 10^16) · x^5

3.2 Amino acid sequence parameters

Table S2. Comparison of hydropathicity and charge for non-identical residues of selected structural FABP domains after triple alignment. Structural domains with the strongest sequence deviations and the lowest similarities were analyzed via Protparam. The net charges and GRAVY values were extracted, highest GRAVY values are marked in red, lowest in blue for each structure domain. In the bottom lines, the values for the complete sequences of each FABP are given for comparison. #GRAVY = grand average of hydropathy – sum of hydropathy values divided by sequence length; increasing positive score = greater hydrophobicity. *The selected FABP shows stronger sequence deviation in this domain than the other two FABPs

Domain	FABP	Partial sequence	GRAVY#	Charge
α2	5	IALRKMGAMA*	0.87	+2
	3	FATRQVASMT	0.23	+1
	4	FATRKVAGMA	0.48	+2
β2-TT	5	TCDGKN	-1.583	0
	3	EKNGDI	-1.717	-1
	4	SVNGDV	0.033	-1
β4-TT	5	TTQFSCTLGEK*	-0.464	0
	3	NTEISFKLGVE	-0.091	-1
	4	NTEISFILGQE	-0.027	-2
β6-TT	5	TQTVCNFTD*	-0.344	-1
	3	VKSIVTLDG	0.822	0
	4	VKSTITLDG	0.278	0
β8-TT	5	KESTITRKLKDG	-1.458	+2
	3	QETTLVRELIDG	-0.333	-2
	4	KSTTIKRKREDD	-2.408	+2
β9-TT-β10	5	ECVMNNVTC	0.456	-1
	3	TLTHGTAVC*	0.733	0
	4	ECVMKGVTS	0.389	0
Binding pocket-related	5	GLACCFCGTCQGCMC	1.327	0
	3	LTATLTFIFVSVHTLLLHC	1.674	0
	4	CFVTAAMIISFIFVSILHTIVCS	2.065	0
Surface- exposed	5	various	-1.869	-6
	3	various	-0.847	-5
	4	various	-1.163	-4
Total protein with all residues	5	See (Michler et al., 2024) *	-0.458	0
	3	See (Michler et al., 2024)	-0.265	-1
	4	See (Michler et al., 2024)	-0.249	0

3.3 Parameters of the EPR simulations

Table S3. Simulation parameters of **free 5/16-DSA.**

Sys	$g_{xx}/g_{yy}/g_{zz}$	$A_{xx}/A_{yy}/A_{zz}$ [MHz]	$\tau_{cxx}/\tau_{cyy}/\tau_{czz}$ (LOG ns)	$Lw_{G/L}$
**(F) 5 °C**				
5-DSA	2.0072 2.0055 2.0026	15.155 12.927 104.189	[-9.8141 -10.2738 -10.2738] * 0.95	0.12/0
16-DSA	2.008 2.0054 2.0023	15.155 12.927 104.179	[-9.8141 -10.2738 -10.2738] * 0.96	0.13/0
**(F) 40 °C**				
5-DSA	2.0071 2.0055 2.0026	15.155 12.927 104.179	[-9.8141 -10.2738 -10.2738] * 0.985	0.12/0
16-DSA	2.0087 2.0055 2.0026	15.155 12.927 104.179	[-9.8141 -10.2738 -10.2738] * 0.999	0.14/0
**(F) 80 °C**				
5-DSA	2.007 2.0055 2.0026	15.155 12.927 104.179	-9.8141 -10.2738 -10.2738] * 1.05	0.13/0
16-DSA	2.0071 2.0055 2.0023	15.155 12.927 104.179	[-9.8141 -10.2738 -10.2738] * 1.06	0.14/0

Table S4. Simulation parameters of **intermediately bound FABP3/16-DSA.**

**Sys**	$g_{xx}/g_{yy}/g_{zz}$	$A_{xx}/A_{yy}/A_{zz}$ [MHz]	$\tau_{cxx}/\tau_{cyy}/\tau_{czz}$ (LOG ns)	$Lw_{G/L}$
**(I) 5 °C**				
50/20	2.01 2.00533 2.001	18.372 18.767 111.194	[-8.83868 -7.93029 -8.13273] *0.998	0/0
100/20	2.008 2.0057 2.0002	15.972 16.967 96.194	[-8.73868 -7.93029 -7.91273] *0.998	0/0
200/20	2.008 2.00543 2.0016	15.672 15.867 103.194	[-8.93868 -7.93029 -8.13273] *0.998	0/0
**(I) 40 °C**				
50/20	2.0102 2.0054 2.002	11.672 16.867 99.194	[-8.93868 -7.08029 -8.25273] *0.998	0/0
100/20	2.0092 2.0052 2.0013	15.972 16.967 95.194	[-8.93868 -7.93029 -7.91273] *0.998	0/0
200/20	2.0090 2.0054 2.0016	12.672 16.867 98.194	[-8.93868 -7.93029 -8.03273] *0.998	0/0
**(I) 80 °C**				
50/20	2.01 2.0053 2.0009	17.372 17.767 94.194	[-8.93868 -7.93029 -8.11273] *0.998	0/0
100/20	2.0092 2.0052 2.0013	16.972 16.967 96.194	[-9.13868 -7.93029 -8.11273] *0.998	0/0
200/20	2.0082 2.0054 2.0012	17.672 17.867 91.194	[-9.23868 -7.93029 -8.63273] *0.999	0/0

Table S5. Simulation parameters of **intermediately bound FABP3/5-DSA.**

**Sys**	$g_{xx}/g_{yy}/g_{zz}$	$A_{xx}/A_{yy}/A_{zz}$ [MHz]	$\tau_{cxx}/\tau_{cyy}/\tau_{czz}$ (LOG ns)	$Lw_{G/L}$
**(I) 5 °C**				
50/20	2.0066 2.0054 2.0022	14.672 14.867 110.194	[-8.73868 -7.93029 -8.33273] *0.998	0/0
100/20	2.0095 2.0054 2.0025	10.672 16.867 105.194	[-8.73868 -7.93029 -8.53273] *0.998	0/0
200/20	-	-	-	-
**(I) 40 °C**				
50/20	2.0082 2.0054 2.0022	15.672 15.867 91.194	[-8.73868 -7.93029 -8.33273] *0.999	0/0
100/20	2.0089 2.0054 2.0025	13.672 16.867 98.194	[-8.73868 -7.93029 -8.33273] *0.998	0/0
200/20	2.0093 2.005 2.0018	15.672 15.867 90.194	[-8.73868 -7.93029 -8.00273] *0.998	0/0
**(I) 80 °C**				
50/20	2.0089 2.0054 2.0025	15.672 15.867 91.194	[-8.73868 -7.93029 -8.33273] *0.999	0/0
100/20	2.0078 2.0054 2.0024	17.672 17.867 100.194	[-8.53868 -7.93029 -8.25273] *0.997	0/0
200/20	2.0089 2.0054 2.0025	10.672 16.867 100.194	[-8.73868 -7.93029 -8.53273] *0.998	0/0

Table S6. Simulation parameters of **strongly bound FABP3/16-DSA.**

**Sys**	$g_{xx}/g_{yy}/g_{zz}$	$A_{xx}/A_{yy}/A_{zz}$ [MHz]	$\tau_{cxx}/\tau_{cyy}/\tau_{czz}$ (LOG ns)	$Lw_{G/L}$
**(S) 5 °C**				
50/20	2.0088 2.0058 2.002	16 16 92	[-7.25527 -7.25527 -8.49815] *0.998	0/0.1
100/20	2.0085 2.0057 2.002	10 10 90	[-7.25527 -7.25527 -8.49815] *0.998	0.2 0.1
200/20	2.008 2.0058 2.0018	15 15 96	[-7.25527 -7.25527 -8.49815] *0.998	0/0.13
**(S) 40 °C**				
50/20	2.009 2.0058 2.0025	10 10 98	[-7.25527 -7.25527 -8.49815] *0.998	0/0.1

100/20	2.0088 2.0057 2.0015	10 10 96	[-7.25527 -7.25527 -8.49815] *0.998	0/0.1
200/20	2.009 2.0058 2.0018	10 10 96	[-7.25527 -7.25527 -8.69815] *0.998	0/0.1
**(S) 80 °C**				
50/20	-	-	-	-
100/20	-	-	-	-
200/20	-	-	-	-

Table S7. Simulation parameters of **strongly bound FABP3/5-DSA.**

**Sys**	$g_{xx}/g_{yy}/g_{zz}$	$A_{xx}/A_{yy}/A_{zz}$ [MHz]	$\tau_{cxx}/\tau_{cyy}/\tau_{czz}$ (LOG ns)	$Lw_{G/L}$
**(I) 5 °C**				
50/20	2.0089 2.0058 2.0025	16 16 98	[-7.25527 -7.25527 -8.77815] *0.998	0/0.1
100/20	2.0093 2.0058 2.0015	10 14 101	[-7.35527 -7.25527 -8.99815] *0.98	0/0.15
200/20	2.0065 2.0058 2.0003	10 10 103	[-7.15527 -7.25527 -8.99815] *0.96	0/0.17
**(I) 40 °C**				
50/20	2.0088 2.0058 2.002	15 15 92	[-7.25527-7.25527 -8.57815] *0.998	0/0.1
100/20	2.0084 2.0058 2.0021	13 13 98	[-7.25527 -7.25527 -8.77815] *0.998	0/0.1
200/20	2.0085 2.0058 2.0017	10 10 96	[-7.25527 -7.25527 -8.69815] *0.98	0/0.1
**(I) 80 °C**				
50/20	-	-	-	-
100/20	2.009 2.0058 2.0015	10 14 98	[-7.35527 -7.25527 -8.99815] *0.98	0/0.15
200/20	2.009 2.0058 2.0025	12 12 100	[-7.25527 -7.25527 -8.99815] *0.98	0/0.15

Table S8. Simulation parameters of **intermediately bound FABP4/16-DSA.**

**Sys**	$g_{xx}/g_{yy}/g_{zz}$	$A_{xx}/A_{yy}/A_{zz}$ [MHz]	$\tau_{cxx}/\tau_{cyy}/\tau_{czz}$ (LOG ns)	$Lw_{G/L}$
**(I) 5 °C**				
50/20	2.0081 2.0054 2.0023	13.672 14.867 102.194	[-8.73868 -7.93029 -8.33273] *0.998	0/0
100/20	2.0082 2.005 2.0013	13.672 14.867 100.194	[-8.73868 -7.93029 -9.13273] *0.998	0/0
200/20	2.008 2.005 2.0018	17.972 17.967 94.194	[-8.93868 -7.93029 -8.69273] *0.998	0/0
**(I) 40 °C**				
50/20	2.008 2.0054 2.0021	13.672 14.867 104.194	[-8.73868 -7.93029 -8.33273] *0.998	0/0
100/20	2.0075 2.0054 2.002	13.672 14.867 103.194	[-8.73868 -7.93029 -9.13273] *0.998	0/0.1
200/20	2.008 2.005 2.0018	15.972 15.967 101	[-8.93868 -7.93029 -8.69273] *0.99	0/0
**(I) 80 °C**				
50/20	2.0081 2.0054 2.0023	13.672 14.867 109.194	[-8.73868 -7.93029 -8.33273] *0.998	0/0
100/20	2.0075 2.0054 2.0023	13.672 14.867 102.194	[-8.73868 -7.93029 -9.14273] *0.99	0/0
200/20	2.0082 2.0055 2.0018	13.972 13.967 100	[-8.99868 -7.93029 -8.69273] *1	0/0

Table S9. Simulation parameters of **intermediately bound FABP4/5-DSA.**

**Sys**	$g_{xx}/g_{yy}/g_{zz}$	$A_{xx}/A_{yy}/A_{zz}$ [MHz]	$\tau_{cxx}/\tau_{cyy}/\tau_{czz}$ (LOG ns)	$Lw_{G/L}$
**(I) 5 °C**				
50/20	2.008 2.0055 2.0021	13.672 14.867 112.194	[-8.73868 -7.93029 -8.33273] *0.998	0/0
100/20	2.0075 2.0054 2.0021	13.672 14.867 110.194	[-8.73868 -7.93029 -8.33273] *0.998	0/0
200/20	-	-	-	-
**(I) 40 °C**				
50/20	2.008 2.0054 2.0021	13.672 14.867 112.194	[-8.73868 -7.93029 -8.33273] *0.998	0/0
100/20	2.0075 2.0054 2.0021	12.672 12.867 101.194	[-8.73868 -7.93029 -8.33273] *0.998	0/0
200/20	2.0078 2.0054 2.0021	13.672 13.867 100.194	[-8.73868 -7.93029 -8.33273] *0.998	0/0
**(I) 80 °C**				
50/20	2.0083 2.0054 2.0021	13.672 14.867 112.194	[-8.73868 -7.93029 -8.33273] *0.998	0/0
100/20	2.0075 2.0054 2.0021	12.672 12.867 101.194	[-8.73868 -7.93029 -8.33273] *0.998	0/0
200/20	2.0078 2.0054 2.0021	15.672 15.867 90.194	[-8.73868 -7.93029 -8.33273] *1	0/0

Table S10. Simulation parameters of **strongly bound FABP4/16-DSA.**

Sys	$g_{xx}/g_{yy}/g_{zz}$	$A_{xx}/A_{yy}/A_{zz}$ [MHz]	$\tau_{cxx}/\tau_{cyy}/\tau_{czz}$ (LOG ns)	$Lw_{G/L}$
**(I) 5 °C**				
50/20	2.0081 2.0058 2.0021	15 15 99	[-7.25527 -7.25527 -8.77815] *0.998	0/0.2
100/20	2.0068 2.0057 2.0015	18 18 101	[-7.25527 -7.25527 -8.77815] *0.998	0 0.1
200/20	2.008 2.0053 2.0025	12 12 97	[-7.25527 -7.25527 -8.49815] *0.998	0 0.1
**(I) 40 °C**				
50/20	2.008 2.0058 2.0021	18 18 93	[-7.25527 -7.25527 -8.77815] *0.998	0/0.1
100/20	2.0075 2.0058 2.002	12 12 94	[-7.25527 -7.25527 -8.77815] *0.998	0/0.1
200/20	2.007 2.0053 2.0025	13 13 93	[-7.25527 -7.25527 -8.49815] *0.998	0/0.1
**(I) 80 °C**				
50/20	-	-	-	-
100/20	-	-	-	-
200/20	-	-	-	-

Table S11. Simulation parameters of **strongly bound FABP4/5-DSA.**

Sys	$g_{xx}/g_{yy}/g_{zz}$	$A_{xx}/A_{yy}/A_{zz}$ [MHz]	$\tau_{cxx}/\tau_{cyy}/\tau_{czz}$ (LOG ns)	$Lw_{G/L}$
**(I) 5 °C**				
50/20	-	-	-	-
100/20	2.0087 2.0058 2.0019	13 14 97	[-7.25527 -7.25527 -8.77815] *0.97	0/0.14
200/20	2.0082 2.0058 2.0021	13 13 97	[-7.24227 -7.24527 -8.77815] *0.985	0.13/0.18
**(I) 40 °C**				
50/20	-	-	-	-
100/20	2.0075 2.0058 2.0021	18 18 97	[-7.25527 -7.25527 -8.77815] *0.998	0/0.13
200/20	2.008 2.0058 2.0021	14 14 97	[-7.25527 -7.25527 -8.77815] *0.997	0/0
**(I) 80 °C**				
50/20	-	-	-	-
100/20	-	-	-	-
200/20	-	-	-	-

Table S12. Simulation parameters of **intermediately bound FABP5/16-DSA.**

Sys	$g_{xx}/g_{yy}/g_{zz}$	$A_{xx}/A_{yy}/A_{zz}$ [MHz]	$\tau_{cxx}/\tau_{cyy}/\tau_{czz}$ (LOG ns)	$Lw_{G/L}$
**(I) 5 °C**				
50/20	2.0083 2.0054 2.0013	16.872 16.867 114.19	[-8.93868 -7.93029 -8.11273] *0.998	0/0
100/20	2.008 2.0054 2.0012	16.872 16.867 114.194	[-8.93868 -7.93029 -8.11273] *0.998	0/0
200/20	2.0098 2.0052 2.0008	13.872 13.867 112.194	[-8.93868 -7.93029 -7.9273] *0.998	0/0
**(I) 40 °C**				
50/20	2.0088 2.0054 2.0011	15.872 15.867 90.19	[-8.93868 -7.93029 -8.11273] *0.998	0/0
100/20	2.0092 2.0054 2.001	13.872 13.867 98.194	[-8.93868 -7.93029 -8.11273] *0.998	0/0
200/20	2.0094 2.0054 2.0015	16.872 16.867 92.194	[-8.93868 -7.93029 -7.91273] *0.998	0/0
**(I) 80 °C**				
50/20	2.0087 2.0054 2.0011	15.872 16.867 88.194	[-8.93868 -7.93029 -8.11273] *0.998	0/0
100/20	2.0092 2.0054 2.001	17.872 17.867 88.194	[-8.93868 -7.93029 -8.31273] *0.999	0/0
200/20	2.0089 2.0054 2.0011	15.872 15.867 91.194	[-8.93868 -7.93029 -8.71273] *0.999	0/0

Table S13. Simulation parameters of **intermediately bound FABP5/5-DSA.**

Sys	$g_{xx}/g_{yy}/g_{zz}$	$A_{xx}/A_{yy}/A_{zz}$ [MHz]	$\tau_{cxx}/\tau_{cyy}/\tau_{czz}$ (LOG ns)	$Lw_{G/L}$
**(I) 5 °C**				
50/20	2.008 2.00543 2.0025	13.672 14.867 108.194	[-8.73868 -7.93029 -8.33273] *0.998	0/0
100/20	2.0082 2.0054 2.0025	15.672 15.867 110.194	[-8.73868 -7.93029 -8.33273] *0.998	0/0
200/20	2.0097 2.0052 2.0012	15.872 16.867 102.194	[-8.93868 -7.93029 -7.91273] *0.998	0/0
**(I) 40 °C**				

50/20	2.008 2.0054 2.0025	13.672 14.867 108.194	[-8.73868 -7.93029 -8.33273] *0.998	0/0
100/20	2.0081 2.0054 2.0025	13.672 14.867 112.194	[-8.73868 -7.93029 -8.33273] *0.998	0/0
200/20	2.0088 2.00543 2.0025	13.672 14.867 108.394	[-8.73868 -7.93029 -8.33273] *0.998	0/0
**(I) 80 °C**				
50/20	2.008 2.0054 2.0025	13.672 14.867 108.194	[-8.73868 -7.93029 -8.33273] *0.998	0/0
100/20	2.009 2.0054 2.002	13.672 14.867 112.194	[-8.73868 -7.93029 -8.53273] *0.998	0/0
200/20	2.0076 2.0058 2.0031	20 20 84	[-7.25527 -7.25527 -9.09915] *0.999	0/0.1

Table S14. Simulation parameters of **strongly bound FABP5/16-DSA.**

**Sys**	$g_{xx}/g_{yy}/g_{zz}$	$A_{xx}/A_{yy}/A_{zz}$ [MHz]	$\tau_{cxx}/\tau_{cyy}/\tau_{czz}$ (LOG ns)	$Lw_{G/L}$
**(S) 5 °C**				
50/20	2.0083 2.0058 2.002	12 12 97	[-7.25527 -7.25527 -8.89815] *0.998	0/0.2
100/20	2.0085 2.0058 2.0019	12 12 97	[-7.25527 -7.25527 -8.89815] *0.998	0/0.2
200/20	2.0093 2.0048 2.002	10 10 98	[-7.85527 -7.25527 -8.79815] *0.998	0/0.1
**(S) 40 °C**				
50/20	2.008 2.0058 2.002	12 12 91	[-7.25527 -7.25527 -8.89815] *0.998	0/0.2
100/20	2.0075 2.0058 2.0025	10 10 91	[-7.25527 -7.25527 -8.49815] *0.998	0/0.1
200/20	2.0075 2.0058 2.0025	10 10 91	[-7.25527 -7.25527 -8.49815] *0.998	0/0.1
**(S) 80 °C**				
50/20	-	-	-	-
100/20	-	-	-	-
200/20	-	-	-	-

Table S15. Simulation parameters of **strongly bound FABP5/5-DSA.**

**Sys**	$g_{xx}/g_{yy}/g_{zz}$	$A_{xx}/A_{yy}/A_{zz}$ [MHz]	$\tau_{cxx}/\tau_{cyy}/\tau_{czz}$ (LOG ns)	$Lw_{G/L}$
**(S) 5 °C**				
50/20	2.0088 2.0058 2.0021	15 15 101	[-7.25527 -7.25527 -8.77815] *0.998	0/0.1
100/20	2.009 2.0058 2.0017	13 13 102	[-7.25527 -7.25527 -8.77815] *0.998	0/0.1
200/20	2.0069 2.0078 2.0021	10 10 102	[-7.95527 -7.35527 -8.69815] *0.997	0/0.1
**(S) 40 °C**				
50/20	2.0088 2.0058 2.0021	15 15 95	[-7.25527 -7.25527 -8.77815] *0.998	0/0.1
100/20	2.0085 2.0058 2.0021	17 17 94	[-7.25527 -7.25527 -8.77815] *0.998	0/0.1
200/20	2.0089 2.0058 2.0031	18 18 87	[-7.25527 -7.25527 -8.77815] *0.998	0/0.1
**(S) 80 °C**				
50/20	2.0075 2.0058 2.0021	15 15 94	[-7.25527 -7.25527 -8.77815] *0.998	0/0.1
100/20	2.009 2.0058 2.0032	17 17 90	[-7.15527 -7.15527 -9.87815] *0.998	0/0.1
200/20	2.0076 2.0058 2.0031	20 20 84	[-7.25527 -7.25527 -9.09915] *0.999	0/0.1

Comment: Due to partially strong component weighting changes between different temperatures, systems and concentrations, as well as noisy spectra in several cases, the simulated parameters can vary to a certain degree although similar environments and g values would be generally expected. It should be notified that g values were not calibrated and B_0 values artificially centered since g tensors were not evaluated in this study. In some cases, component percentages become very small and the simulated parameters become more error-prone. To reduce the RMSD values, g- and A-tensors as well as linewidths (G… Gaussian, L… Lorentzian) were fine-tuned, leading to deviating tensor values. We therefore recommend to focus on the rotational correlation times as component-determining parameter. These are given in vectors as decadic log-values with a weighting factor (*). Regarding the component percentage errors, it can still be expected that they show rather systematic errors than random outliers within a series due to the systematic simulation process. For a more compact comparison and discussion the of A-tensors at consistent, physiological conditions or some general temperature-dependent trends of A-tensors we refer to our previous publication (Michler et al., 2024).

3.4 Results of linear/non-linear van't Hoff fitting for selected cases

Table S16. Results of linear (L) and non-linear (NL) van't Hoff fits for selected samples.

	**FABP3/16-DSA 50/20**	**FABP3/16-DSA 100/20**	**FABP3/16-DSA 200/20**	**FABP5/16-DSA 50/20**	**FABP5/16-DSA 100/20**
**F-T**					
ΔH (L) J/mol	-36222.41± 7578.85	-84561.95 ± 10776.8	-42713.55± 3969.95	-26052.29± 4131.06	-19060.55 ± 3988.25
ΔS (L) J/mol/K	-10.25±24.42	-153.35 ± 32.52	-21.87 ± 11.98	12.77 ± 13.31	33.12 ± 12.85
ΔC_p (NL) J/mol/K	-1663.75 ± 303.82	-5.45 ± 1395.51	-6.03 ± 511.06	-915.99± 159.93	-952.17± 99.98
R^2 (L)	0.6955	0.9249	0.9586	0.7991	0.6955
R^2 (NL)	0.9297	0.9243	0.9590	0.9567	0.9725
**F-I**					
ΔH (L) J/mol	-27780.86 ± 9281.20	-84402.09± 10802.67	-32262.83± 5070.49	-16658.57± 5233.86	-9179.38± 5881.09
ΔS (L) J/mol/K	14.99 ± 29.91	-152.89 ± 32.60	7.64± 15.30	40.38 ± 16.86	62.44 ± 18.95
ΔC_p (NL) J/mol/K	-2086.74 ± 340.36	-36.03 ± 1403.19	7.75 ± 659.36	-1209.41 ± 167.87	-1399.22 ± 152.47
R^2 (L)	0.4726	0.9243	0.8901	0.5032	0.1959
R^2 (NL)	0.8981	0.9237	0.8890	0.9266	0.9224
**F-S**					
ΔH (L) J/mol	-82482.33± 18097.62	-	-60315.93 ± 11374.38	-30923.88± 5806.93	-29566.23± 1737.69
ΔS (L) J/mol/K	-187.33 ± 63.38	-	-86.96 ± 35.82	-12.35± 19.86	-12.89± 5.94
ΔC_p (NL) J/mol/K	-78.59 ± 5236.01	-	-	11.18± 1321.24	15.85± 396.74
R^2 (L)	0.8738	-	0.9336	0.8501	0.9830
R^2 (NL)	0.8764	-	-	0.8490	0.9828
**I-S**					
ΔH (L) J/mol	-108682.40 ± 19403.62	-113026.27± 18579.00	-45184.65 ± 6362.83	-42277.93± 4271.34	-52332.66± 6156.05
ΔS (L) J/mol/K	-389.11 ± 67.95	-402.36 ± 65.06	-149.22 ± 21.35	-149.46± 14.61	-185.73± 21.06
ΔC_p (NL) J/mol/K	-155.39 ± 5566.69	-162.89 ± 5332.65	-4.57 ± 873.70	-5.82± 966.92	-12.45± 1401.54
R^2 (L)	0.9127	0.925	0.8937	0.9514	0.9353
R^2 (NL)	0.9159	0.9277	0.8935	0.9516	0.9347
